# The chromosome-level genome assembly of an endangered herb *Bergenia scopulosa* provides insights into local adaptation and genomic vulnerability under climate change

**DOI:** 10.1093/gigascience/giae091

**Published:** 2024-11-28

**Authors:** Yi-Xin Yang, Meng Wang, Xuan-Ye Wu, Ya-Ni Zhou, Jie Qiu, Xia Cai, Zhong-Hu Li

**Affiliations:** Key Laboratory of Resource Biology and Biotechnology in Western China, Ministry of Education, Provincial Key Laboratory of Biotechnology, College of Life Sciences, Northwest University, Xi'an 710069, China; Medical Experiment Center, Shaanxi University of Chinese Medicine, Xianyang 712046, China; Key Laboratory of Resource Biology and Biotechnology in Western China, Ministry of Education, Provincial Key Laboratory of Biotechnology, College of Life Sciences, Northwest University, Xi'an 710069, China; Key Laboratory of Resource Biology and Biotechnology in Western China, Ministry of Education, Provincial Key Laboratory of Biotechnology, College of Life Sciences, Northwest University, Xi'an 710069, China; Key Laboratory of Resource Biology and Biotechnology in Western China, Ministry of Education, Provincial Key Laboratory of Biotechnology, College of Life Sciences, Northwest University, Xi'an 710069, China; Key Laboratory of Resource Biology and Biotechnology in Western China, Ministry of Education, Provincial Key Laboratory of Biotechnology, College of Life Sciences, Northwest University, Xi'an 710069, China; Key Laboratory of Resource Biology and Biotechnology in Western China, Ministry of Education, Provincial Key Laboratory of Biotechnology, College of Life Sciences, Northwest University, Xi'an 710069, China; Key Laboratory of Resource Biology and Biotechnology in Western China, Ministry of Education, Provincial Key Laboratory of Biotechnology, College of Life Sciences, Northwest University, Xi'an 710069, China

**Keywords:** *Bergenia scopulosa*, genome assembly, local adaptation, genomic vulnerability, conservation

## Abstract

**Background:**

Global climate change poses severe threats to biodiversity and ecosystem stability. Rapid climate oscillations potentially lead to species geographic range shifts, population declines, and even extinctions. The rare and endangered species, being critical components of regional biodiversity, hold the key to understanding local adaptation and evolutionary processes shaping species distributions. Therefore, assessing the evolutionary mechanisms of local adaptation and population vulnerability under climate change is crucial for developing conservation strategies of endangered species.

**Results:**

In this study, we assembled a high-quality, chromosome-level genome of the rare and endangered herb *Bergenia scopulosa* in the Qinling Mountains in East Asia and resequenced 37 individual genomes spanning its entire geographic distributional ranges. By integrating population genetics, landscape genomics, and climate datasets, a substantial number of adaptive single-nucleotide polymorphism loci associated with climate variables were identified. The genotype–environment association analysis showed that some cold-tolerant genes have played pivotal roles in cold environmental adaptation of *B. scopulosa*. These findings are further corroborated through evolutionary analysis of gene family and quantitative PCR validation. Population genomic analysis revealed 2 distinct genetic lineages in *B. scopulosa*. The western lineage showed higher genomic vulnerability and more rare cold-tolerance alleles, suggesting its heightened sensitivity to impending climate shifts, and should be given priority conservation in the management practices.

**Conclusions:**

These findings provide novel insights into local adaptation and genomic vulnerability of *B. scopulosa* under climate change in the Qinling Mountains in East Asia. Additionally, the study also offers valuable guidance for formulating conservation strategies for the rare and endangered plants.

## Introduction

Biodiversity is the material foundation for the survival of all life on Earth. It is a crucial guarantee for maintaining good operation of ecosystems and serves as a source of materials for human life and production, which is closely related to human survival and development [1]. However, since the onset of the Industrial Revolution in the 18th century, and particularly during the Anthropocene era, human activities have an ever-increasing impact on the natural world. The ensuing global climate fluctuations have led to the fragmentation of habitats and a decline in the population of most organisms. One of the consequences is a decrease in gene flow and/or genetic exchange between populations, which leads to a reduction in the sharing of adaptive alleles. In extreme cases, this has led to the localized extinction of certain species, which poses a significant threat to both biodiversity and the stability of ecosystems [2–4]. When the rate of climate change surpasses the species’ ability to adapt its own environments, it becomes challenging for most plants to adapt to rapidly shifting climates through migration or dispersion [5–7]. Therefore, it is particularly important to evaluate how plant species adapt to complex and ever-changing environments and predict their response mechanisms to future climate changes.

In 2018, Bay et al. [8] proposed the concept of genomic vulnerability as a genotype–environment relationship modeled on contemporary population data to predict the mismatch between current and future genetic variations in the genome of species under changing climate conditions. The concept aims to pinpoint the most vulnerable species to the effects of climate change. A lower degree of match indicates a population’s lesser ability to adapt quickly to future climate change. Thus, genomic vulnerability can be used as an indicator to assess the decline in population size and adaptive capacity. It aids in comprehending and predicting the dynamic changes in population sizes and has garnered growing interest among researchers [9–11]. However, previous studies have frequently emphasized the ecological adaptability of species’ distribution ranges under various climatic scenarios. These analyses primarily rely on species distribution data and environmental variables, neglecting the influence of genetic factors on biological adaptability [12–15]. In recent years, the integration of high-throughput genetic data with environmental factors through landscape genomics analysis methods has gained increasing interest. The evaluation of endangered populations’ response capacity to climate change through this approach has become a prominent and widely discussed topic [16–19]. Traditional methods for obtaining adaptive phenotypic data involve “common garden experiments” or “reciprocal transplant experiments.” Subsequently, genetic and phenotypic data are correlated through methods such as genome-wide association studies (GWAS) or quantitative trait locus (QTL) mapping, providing insights into the intricate relationship between genotype and phenotype. However, these techniques are not practical for studying nonmodel species in their natural habitats due to long experimental cycles and the challenges of obtaining adaptive phenotypic traits [5]. As next-generation sequencing (NGS) and whole-genome sequencing (WGS) technologies continue to advance rapidly, the availability of genetic markers for analysis has gradually increased, enabling more comprehensive and accurate genetic studies. Leveraging landscape genomics approaches, genome-wide scans can now be conducted to identify loci associated with adaptive evolution. By linking these genetic signals with environmental data, genetic loci involved in climate adaptation can be precisely screened out, genetic offset measured, genetic variation integrated with spatial models, and the molecular mechanisms behind local adaptation revealed through genotype–environment associations (GEAs) by researchers. This approach offers insights into the genetic basis of species’ adaptability to their environmental and addresses how much genetic variation is necessary for populations to cope with environmental changes. Moreover, it aids in determining priority conservation efforts for vulnerable populations and holds significant scientific value in managing and formulating conservation strategies for species facing threats under changing climatic conditions. The integration of landscape genomics with advanced sequencing technologies offers immense potential in understanding the intricate relationship between genetics, environment, and adaptation. By applying this knowledge, informed decisions can be made to protect and conserve species in the face of ongoing climate change [9, 20–24].

Researching the genomic vulnerability of species typically involves conducting whole-genome sequencing or resequencing of all individuals within a population to obtain an extensive set of single-nucleotide polymorphism (SNP) markers. By investigating nucleotide variation sites spanning the entire genome, the genetic variation level of the population is comprehensively assessed. Subsequently, association analysis with environmental data is performed to identify specific gene sites under selection and evaluate genetic offset. This comprehensive approach enables the assessment of genomic vulnerability, offering insights into populations that are particularly susceptible to the effects of climate change [11, 19, 25, 26]. *Bergenia scopulosa* T. P. Wang (NCBI:txid1397840) is an endangered perennial herb belonging to the Saxifragaceae family. The dried rhizomes of *B. scopulosa* have been used as traditional medicine in China, particularly in the Qinling area in East Asia, where it is known as Pan Long Qi [27]. This species is sporadically distributed in the damp undergrowth of forests or in the crevices of cliff faces, exhibiting strong cold resistance. In recent years, most studies have primarily concentrated on the genomic vulnerability of woody plants, while herbaceous plants have received limited attention. Herbaceous plants possess a shorter generation cycle and are highly susceptible to the effects of climate change. Therefore, conducting research on the population history and local adaptation of herbaceous species with relatively narrow habitats can comprehensively help one to understand the interaction between geographical and environmental heterogeneity and can also address the vulnerability of small- and medium-sized populations in biodiversity hotspots, which is helpful for the protection and management of threatened species. In this study, our focus is on the rare and endangered medicinal plant *B. scopulosa*, which is endemic to the Qinling Mountains region in China. Through preliminary research and extensive population sampling, we successfully sequenced the genome of *B. scopulosa* at the chromosome level. Subsequently, we conducted resequencing analysis on 37 individuals from 9 populations covering its natural geographic distributions. Using a combination of population genetics, landscape genomics, and environmental modeling methods, we first investigated the genetic structure and population demographic history of this species. Additionally, we assessed the capability of different populations to adapt to climate change based on environmental data. Furthermore, we explored the molecular mechanisms underlying adaptation to diverse climatic conditions. Finally, we evaluated the genomic vulnerability of various geographic populations of *B. scopulosa* in the face of rapid climate change expected in the future.

## Results

### Genome sequencing, assembly, and annotation

**Figure 1:**
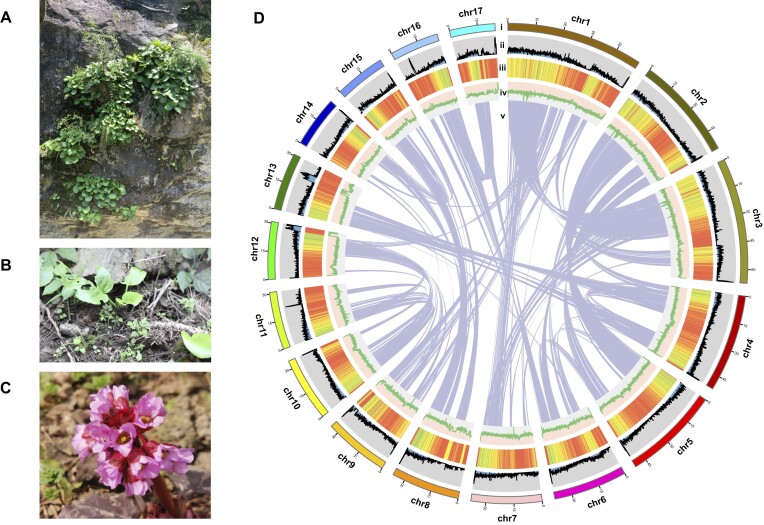
Habitat, morphological, and genomic characteristics of *B. scopulosa*. (A) Typical habitat of *B. scopulosa* growing in the crevices of cliffs and rocks in the TYP population. (B) Individual wild *B. scopulosa* in the TBS population. (C) Flowers of *B. scopulosa*. (D) Overview of the *B. scopulosa* draft genome assembly. (i) The 17 assembled *B. scopulosa* chromosomes. (ii) The gene count along the genome. (iii) The repetitive sequence density along the genome. (iv) The GC content along the genome. (v) Syntenic relationships among different chromosomes of *B. scopulosa*.

A total of 26,925,281,224 *k*-mers of length 17 were generated and the peak depth was 36. The genome size of *B. scopulosa* was estimated to be 737.09 Mb with a heterozygosity of 0.87% and a repeat sequence ratio of 63.04% (Supplementary Table S1; Supplementary Fig. S1). After trimming and quality control, 94.92 Gb (∼129× coverage) of short reads, 22.73 Gb (∼31× coverage) of long reads, and 113.30 Gb (∼154× coverage) of raw Hi-C data were generated (Supplementary Table S2). The final assembly captured 733.32 Mb of the genome sequence, with contig N50 of 20.84 Mb and a chromosome‐size scaffold N50 of 37.96 Mb (Supplementary Table S3). Over 92.86% of the contig sequences (∼680.95 Mb) were successfully anchored to 17 pseudo-chromosomes (Fig. 1; Supplementary Table S4; Supplementary Fig. S2). We evaluated the completeness of the *B. scopulosa* genome using BUSCO. Evaluation against 3 databases indicated that the genome is between 98.7% and 99.5% complete, highlighting the high quality of our assembled genome (Table 1; Supplementary Table S5; Supplementary Fig. S3).

**Table 1:** Statistics of *B. scopulosa* genome assembly and annotation

Feature	Statistic
Assembled genome size (bp)	733,357,027
GC content (%)	36
Contig number	1,083
Contig N50 (bp)	20,841,689
Scaffold number	757
Scaffold N50 (bp)	37,958,852
Minimum len (bp)	17,547
Maximum len (bp)	73,384,106
Mean len (bp)	968,768
Median len (bp)	36,832
Number of annotated genes	45,222
Repeats in genome (%)	67.36
Average BUSCO (complete) (%)	99.1

We predicted 45,222 protein‐coding genes in the *B. scopulosa* genome using *ab initio*, homology‐based, and transcriptome‐based gene prediction methods. To initiate a functional exploration of the *B. scopulosa* genome, we submitted all gene models to the NR, Swissport, EggNOG, COG, TrEMBL, Gene Ontology (GO), and KEGG databases. Of all genes, 42,119 (92.18%) were annotated in these databases (Supplementary Table S6). Concurrently, our investigation led to the identification of a set of noncoding RNAs (Supplementary Table S7). Further analysis revealed that 67.36% of the assembled genome is composed of repetitive sequences, with a predominant composition of 33.58% retroelements and 0.96% DNA transposons. Long terminal repeat (LTR) retrotransposons constituted a significant portion, encompassing 25.19% of the genome, with Ty1/copia (8.84%) and gypsy/DIRS1 (10.74%) being notable contributors (Supplementary Table S8; Supplementary Fig. S4).

### Evolution and phylogeny of the *B. scopulosa* genome

**Figure 2:**
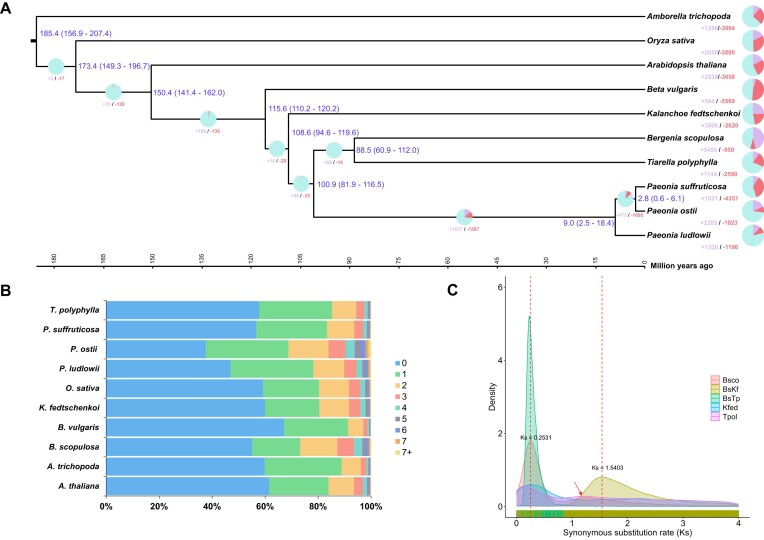
Comparative genomic analysis of *B. scopulosa* and its related species. (A) Phylogenetic tree representing the number of gene families that have expanded or contracted among 10 species. The pie charts show the percentage of expanded (purple), contracted (red), and conserved (blue) gene families across all gene families. The estimated divergence time (in millions of years) is shown beside the branch nodes in blue, and the numbers enclosed in parentheses indicate the confidence interval for the estimated divergence time. The scale on the x-axis shows the estimated divergence time for nodes. “+” indicates that gene families expanded, and “−” indicates that gene families contracted. (B) Number of paralogous gene families among 10 species. (C) Kernel-density estimates of Ks distributions for one-to-one orthologs (reciprocal best hits) among *B. scopulosa, K. fedtschenkoi*, and *T. polyphylla*. Bsco: *B. scopulosa*; BsKf: *B. scopulosa*–*K. fedtschenkoi*; BsTp: *B. scopulosa*–*T. polyphylla*; Kfed: *K. fedtschenkoi*; Tpol: *T. polyphylla*.

A comparative genomic analysis of *B. scopulosa* was performed with 9 other plant genomes. These 10 species shared 31,623 gene families (orthogroups), with 691 gene families comprising 5,658 species-specific genes unique to *B. scopulosa*. Phylogenetic inference based on 299 single-copy orthologous genes showed that *B. scopulosa* diverged approximately 108.6 million years ago (Mya) from *Kalanchoe fedtschenkoi* (Fig. 2A, B). The Ks value of *B. scopulosa*–*K. fedtschenkoi* (BsKf) was 1.5403. Using the formula (T = Ks/2r), we determined that the recent whole-genome duplication (WGD) event in *B. scopulosa* (Ks = 0.2531) occurred approximately 17.85 Mya. In the vicinity of this Ks value, we discerned pronounced peaks indicative of recent WGD events in *B. scopulosa*. Notably, our analysis revealed that both *Tiarella polyphylla* and *B. scopulosa*, members of the Saxifragaceae family, exhibit a distinct ancient peak at approximately Ks = 1.2. After adding *Vitis vinifera* for analysis, we found that this peak appeared before the divergence peak between *B. scopulosa* and *V. vinifera*, suggesting that *B. scopulosa* experienced the same gamma WGD event as *V. vinifera* and *T. polyphyllac*. Moreover, the microsynteny patterns further support 2 WGDs on the lineages leading to *B. scopulosa*. Specifically, the microsynteny pattern reflects a 4:1 gene copy ratio between the *B. scopulosa* genome and the *V. vinifera* genome (Fig. 2C; Supplementary Fig. S5). Furthermore, gene family evolution analysis revealed that 5,495 gene families had expanded, constituting 46.20% of all gene families, while 950 gene families had contracted (7.99% of the total). Notably, 329 expanded and 5 contracted gene families exhibited statistical significance (*P* < 0.05) in *B. scopulosa*.

To understand their biological functions, we conducted KEGG and GO analyses. GO analysis highlighted that the significantly expanded gene families were enriched in processes such as response to salicylic acid (GO:0009751), regulation of flavonoid biosynthetic process (GO:0009962), response to gibberellin (GO:0009739), regulation of seed development (GO:0080050), regulation of translation in response to stress (GO:0043555), response to jasmonic acid (GO:0009753), and response to light intensity (GO:0009642) (Supplementary Table S9). In KEGG analysis, most of the expanded genes were clustered in oxidative phosphorylation (ko00190), photosynthesis (ko00195), flavonoid biosynthesis (ko00941), biosynthesis of various plant secondary metabolites (ko00999), and plant hormone signal transduction (ko04075) (Supplementary Table S10). The contracted gene families were associated with GO terms linked to hydroxyjasmonate sulfotransferase activity, monocarboxylic acid metabolic process, organic acid metabolic process, response to desiccation, small-molecule metabolic process, and so on (Supplementary Table S11). Furthermore, KEGG pathways analysis for the contracted genes indicated their involvement in ubiquitin-mediated proteolysis; phenylalanine, tyrosine, and tryptophan biosynthesis; and biosynthesis of various plant secondary metabolites (Supplementary Table S12). We posited that the expansion and contraction of these genes might enhance the adaptability of *B. scopulosa* in complex environments, allowing it to better cope with stress and regulate its growth, ensuring survival under abiotic stress conditions [28–32].

### Population structure, genetic diversity, and demographic history

**Figure 3:**
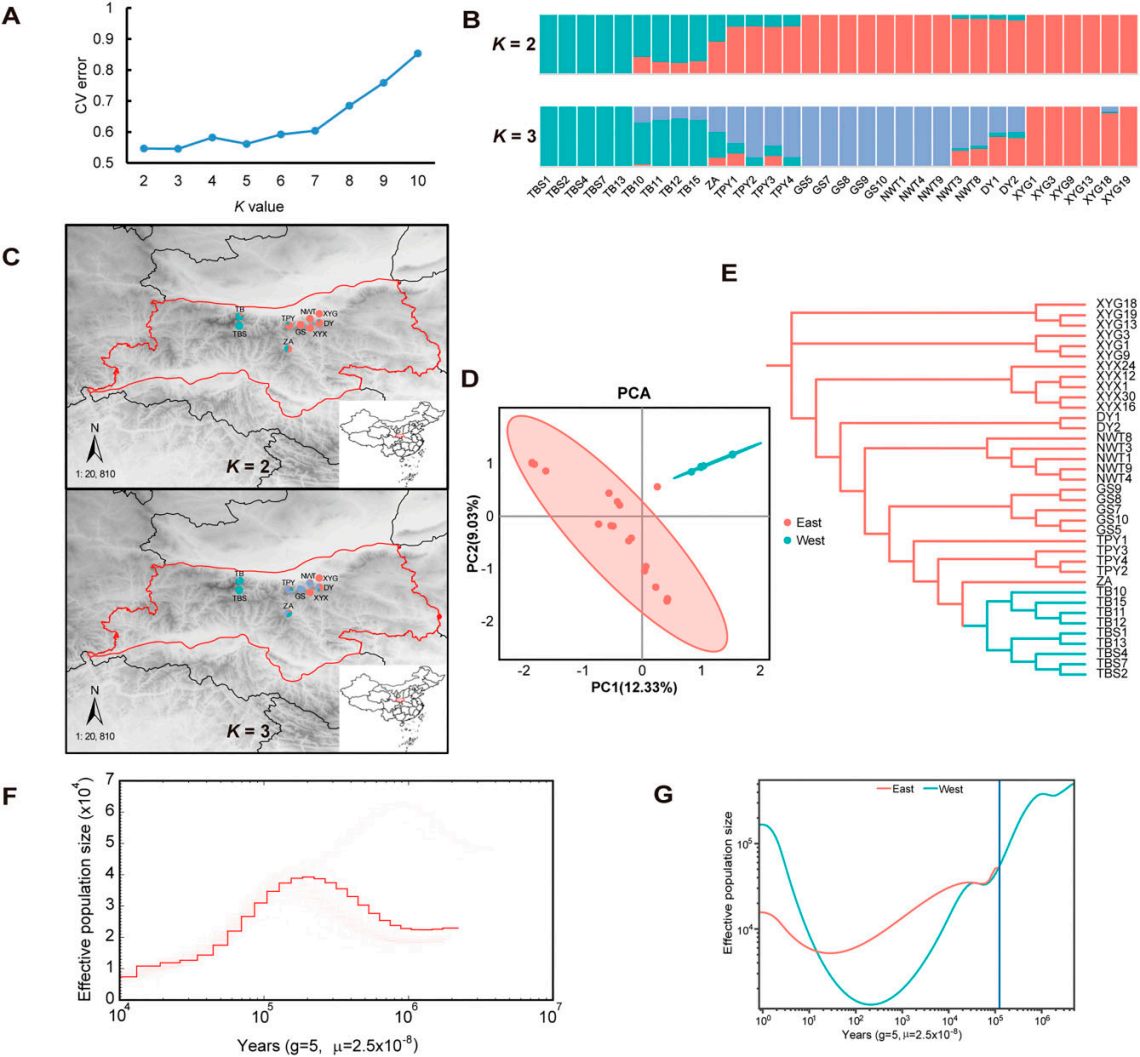
Population structure, genetic diversity, and demographic history of *B. scopulosa*. (A) ADMIXTURE cross-validation errors for each *K* value (2–10). The *K* = 3 is optimal. (B) Admixture analysis with individual ancestry coefficients *K* = 2, 3. (C) Spatial genetic structure of *B. scopulosa* based on 9 populations within the natural species range in China. The pie chart colors indicate the probability of sample assignment based on SNPs analyzed using ADMIXTURE at *K* = 2 and 3. (D) PCA of SNPs identified from the resequenced individuals. The first 2 principal components (PC1, 12.33% vs. PC2, 9.03%) are shown. (E) Nonrooted ML phylogenetic tree based on SNPs in 37 individuals. Color coding of the branches reflects the structure of genetic groups at *K* = 2. (F) Past effective population size history of *B. scopulosa* assessed by PSMC. (G) Demographic changes on recent time scales established for west and east lineages by using SMC++.

To explore genetic variation in *B. scopulosa*, we resequenced 37 individuals from 9 wild populations across the entire range of its distribution in the Qinling Mountain areas with an average depth of ∼25× (Supplementary Fig. S6; Supplementary Table S13). Based on our high-quality genome as a reference, 13,044,067 SNPs were obtained. In population genetic analysis, although the cross-validation (CV) error value is minimized at *K* = 3 (cv_error = 0.5457) (Fig. 3A), the 9 populations of *B. scopulosa* exhibit a clear division into 2 distinct lineages (West and East) when *K* = 2 (cv_error = 0.5466). These lineages correspond to the geographical locations on the east and west sides of the Qinling Mountains (Fig. 3B, C) in East Asia, which is further reinforced by the findings obtained from the principal component analysis (PCA) (Fig.   3D) and nonrooted branching maximum likelihood (ML) phylogenetic tree (Fig. 3E). Notably, when *K* = 3, the eastern lineage underwent further genetic differentiation within, whereas the western lineage remains relatively stable (Fig. 3C). Given this observation, we have decided to select the geographically distinct eastern and western lineages for subsequent in-depth analysis. Analysis of nucleotide diversity (*π*) revealed that the eastern lineage exhibited higher genetic diversity compared to the western lineage (Supplementary Fig. S7). Notably, regions of the genome with elevated differentiation and reduced diversity were pinpointed between the eastern and western lineages (Supplementary Fig. S8). Further investigation into the functional analysis of genes within these regions revealed a significant correlation with plant stress resistance (Supplementary Fig. S9; Supplementary Table S14). This finding underscores the potential role of these genomic regions and associated genes in shaping the adaptive responses of *B. scopulosa* populations to their environment [33–39].

To investigate the demographic history of this species, we used the pairwise sequential Markovian coalescent (PSMC) analysis over a substantial time scale. It unveiled a decline in the effective population size, plummeting from approximately 3.9 × 10^4^ to 0.75 × 10^4^ individuals around 0.19 Mya (Fig. 3F). For a more precise understanding of recent population changes in the 2 lineages, the SMC++ was used for population statistics modeling. Our results showed a rapid population growth in 2 lineages over the past 25–200 years and suggested their divergence occurred ∼130,000 years ago (Fig. 3G).

### Genome–environment association revealed genetic loci associated with local adaptation

Two genome–environment association methods—latent factor mixed models (LFMMs) and redundancy analysis (RDA)—were utilized to delve into the genetic mechanisms underlying local adaptation of *B. scopulosa*. Leveraging high-quality variant SNPs identified in the earlier genetic analysis, we conducted machine learning regression analysis using the “gradient Forest” package in R to execute gradient forest analysis. After considering variable importance and correlations, we identified 5 climate variables most strongly linked to genetic variation (i.e., BIO3: Isothermality, BIO4: Temperature Seasonality, BIO15: Precipitation Seasonality, BIO18: Precipitation of Warmest Quarter, BIO19: Precipitation of Coldest Quarter) (Supplementary Fig. S10). Subsequently, we utilized LFMMs to examine the correlation between predictive variables and allele frequencies. A total of 40,529 SNPs colocalized with 1,702 genes were identified across 5 environmental factors. The terms with the significant enrichment levels were plant hormone signal transduction (ko04075, *P* = 9.06 × 10^−3^) and circadian rhythm—plant (ko04712, *P* = 4.25 × 10^−2^) (Supplementary Fig. S11). Within the realm of plant hormone signal transduction, our investigation has unveiled several key genes that orchestrate the plant’s adaptive response to adverse conditions. Specifically, *BsSAUR14* (*Bsco_041147*) was colocalized with 1 SNP chr14_25686183 (*P* = 6.19 × 10^−4^), which was identified using LFMMs for BIO4, hinting at its role in stress tolerance [40]. Furthermore, delving into the circadian rhythm—plant pathway revealed a profound connection between diurnal regulation and plant resilience, with notable genes including *BsCRY1* (*Bsco_034373*, chr4_14735339, *P* = 1.79 × 10^−5^) and *BsPhyA* (*Bsco_006033*, chr6_8489173, *P* = 4.69 × 10^−4^), both implicated in the plant’s ability to withstand stressful environments [41, 42]. Additionally, we conducted a multivariate regression analysis combined with RDA on the allele frequency data of *B. scopulosa* with 5 climate variables selected and found that environmental factors can explain 44.11% of the genomic variation. RDA1 and RDA2 accounted for 24.98% and 7.07% of the genetic variation (Supplementary Fig. S12), respectively, indicating that environmental factors play a significant role in the genetic diversity and local adaptation of *B. scopulosa*. Specifically, 11,163 outlier SNPs were detected in 6 RDAs, and 1,440 adaptive genes were identified in the 100-kb interval around these outliers. Remarkably, our study also identified a cohort of genes implicated in plant stress tolerance, despite their failure to reach statistical significance within the KEGG pathway analysis framework. Among these genes, notable mentions include *BsHSP70* (*Bsco_031051*, located at chr16_15031245, with an RDA score of −0.3340 in RDA5) associated with the endocytosis pathway (ko04144, *P* = 0.057) and *BsPhyA* (*Bsco_006033*, located at chr6_8493265, with an RDA score of 0.2235 in RDA5) in the circadian rhythm—plant pathway (ko04712, *P* = 0.064), which was also selected by the LFMM method, highlighting its potential significance [43] (Supplementary Fig. S13). Collectively, these genes underscored the pivotal roles they play in orchestrating the adaptability and resilience of plants, particularly those belonging to rare and endangered species capable of enduring cold conditions. Their ability to thrive in harsh environments is intimately tied to the intricate regulation of these crucial genetic factors. In summary, our analysis has pinpointed 931 core SNPs, represented by overlapping markers, that are consistently identified by both the LFMM and RDA methods. Notably, a discrepancy of 272 genes emerges between the 2 approaches, highlighting the significance of employing diverse algorithms to achieve a more comprehensive and robust identification of adaptive genes.

### Freezing tolerance–related genes involved in the local adaptation of *B. scopulosa*

**Figure 4:**
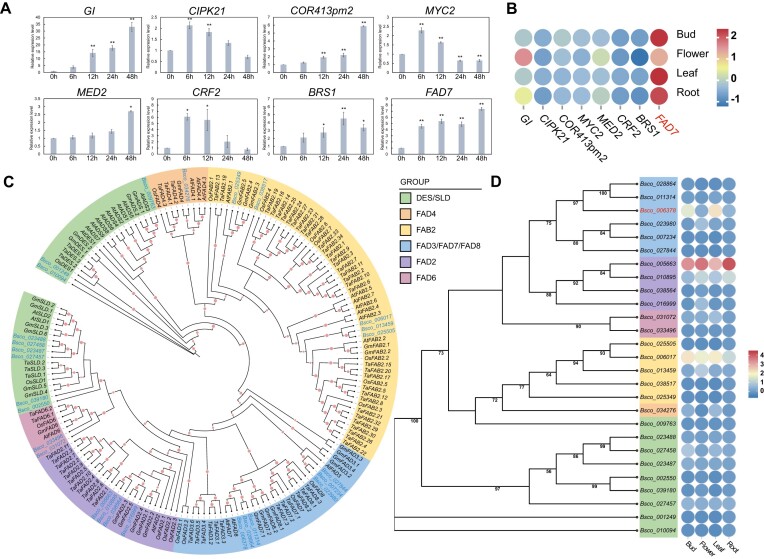
Genome-wide screening of the freezing tolerance–related loci with local adaptation. (A) The mRNA relative expression levels at 0, 6, 12, 24, and 48 hours under cold treatment in 8 selected genes from a sterile seedling of *B. scopulosa*. Data are presented as mean ± SE (*n* = 3). “*” and “**” indicate a significant difference from that of 0 hours at *P* ≤ 0.05 and *P* ≤ 0.01, respectively, by Student’s *t*-test. (B) The log_10_ (FPKM) expression values of 8 candidate genes are represented by a color heatmap ranging from blue to red across 4 tissues: bud, flower, leaf, and root. The color gradient ranging from blue to red indicates low to high expression levels. (C) Phylogenetic relationships of *FAD* genes from *B. scopulosa* (Bs), *Arabidopsis* (At), wheat (Ta), rice (Os), and soybean (Gm). The colored branch shows a different subfamily. The tree was constructed using IQ-tree software by the ML method with 1,000 bootstraps, and the pale red dots in the figure indicate the bootstrap value. (D) Phylogenetic relationships and expression heatmap in different tissues of 27 *FAD* genes from *B. scopulosa*. The *BsFAD7* (*Bsco_006378*) gene, highlighted in red, has been validated in this study.

In this study, cold-related adaptive genes in the 100-kb interval around outlier SNPs were successfully identified using both LFMM and RDA algorithms (Supplementary Table S15), including *GI* (*Bsco_012979*), *CIPK21* (*Bsco_033313*), *COR413pm2* (*Bsco_041002*), *MYC2* (*Bsco_038291*), *MED2* (*Bsco_041019*), *CRF2* (*Bsco_036510*), *BRS1* (*Bsco_031057*), and *FAD7* (*Bsco_006378*). These genes are known to play crucial roles in cold tolerance and adaptation [44–48], highlighting the significance in *B. scopulosa*’s ability to thrive under challenging cold conditions. To validate the expression of these genes, we subjected sterile seedlings of *B. scopulosa* to cold (4°C) treatment and conducted quantitative PCR (qPCR) analysis at 5 time periods (0, 6, 12, 24, and 48 hours). Compared to the untreated group (0 hours), significant differences in gene expression were observed (Fig.   4A), indicating their involvement in the response to cold stress. By examining their expression patterns at different time points, we have gained a deeper understanding of the regulatory mechanisms underlying cold adaptation in *B. scopulosa*. We further conducted an analysis of the expression patterns of these 8 genes across various tissues and found that the *FAD7* (omega-3 fatty acid desaturase 7, *Bsco_006378*) gene was particularly prominent in all tissues (Fig. 4B). Consequently, we delved into the evolutionary relationships within the *FAD* gene family of *B. scopulosa*. To this end, a phylogenetic tree was constructed using 27 predicted FAD proteins from *B. scopulosa* and 143 FAD proteins from *Arabidopsis*, wheat, rice, and soybean. The results indicated that the 27 FAD members of *B. scopulosa* can be categorized into 6 distinct groups, including DES/SLD, FAB2, FAD2, FAD4, FAD3/FAD7/FAD8, and FAD6 (Fig. 4C). Notably, *BsFAD7* (*Bsco_006378*) exhibited the highest expression across almost all tissues within the FAD3/FAD7/FAD8 group (Fig. 4D), strongly suggesting its significant role in cold resistance.

### Genomic offset prediction for future climate change

**Figure 5:**
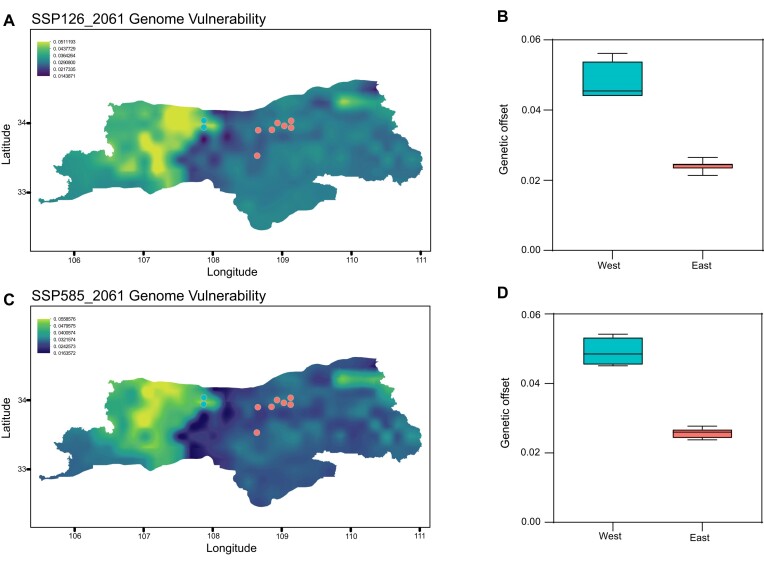
Predicted genetic offset of SNPs across *B. scopulosa* distribution in the future. (A, C) ssp_126 and ssp_585 scenarios in 2061–2080; lighter hues indicate higher genetic offset (higher expected vulnerability to climate change). The circle signifies each population, and the hue denotes whether they pertain to the chilly western lineage (depicted in blue) or the warm eastern lineage (depicted in red). The mapped area is the Qinling Mountains region within Shaanxi Province, China. (B, D) Estimated genetic offsets combined across all populations in 2 lineages.

To elucidate the population-level vulnerability to climate change, we employed a visualization technique known as “genetic offset” that incorporates different climate scenarios within geographic space. By mapping the genetic offset, we can identify areas where certain populations exhibit greater or lesser resilience to changing climatic conditions. This visualization method helps us understand the extent of vulnerability and informs targeted interventions and mitigation strategies. The GF modeling analysis revealed that the western lineage of *B. scopulosa* in the Taibai Mountains is projected to have high genetic offset values in response to future climate scenarios (ssp_126 and ssp_585) during the periods 2061–2080. It suggests that the west lineage is comparatively more susceptible to future environmental changes. Given the rich diversity of genetic resources adaptable to various climatic conditions in the west lineage, prioritizing and enhancing conservation efforts, along with targeted protection measures, for the populations in this region is essential (Fig. 5).

## Discussion

Global climate change is indeed a significant concern as it poses threats to biodiversity and ecosystem stability [49–52]. Rare and endangered species are crucial for regional biodiversity, especially when the populations are extremely small and at risk of extinction. These species are prone to genetic drift and mutation loads, which can hinder their ability to adapt to the local environment and ultimately threaten their survival. Such challenges are closely linked to climate fluctuations [53, 54]. Therefore, comprehending local adaptation mechanisms and genomic vulnerabilities of species in the face of climate change is crucial for crafting conservation policies protecting rare and endangered species. *B. scopulosa*, an endangered herb species in the Qinling Mountains in East Asia, offers valuable medicinal properties and survives even during severe winters (Fig. 1). This species is an excellent candidate for exploring the genetic mechanisms of local adaptation. However, limited high-quality genome data have hindered the present research. To address this issue, we utilized advanced sequencing and chromosomal mapping techniques to generate a high-quality chromosome-level genome for this endangered species. Further analysis was conducted on population genomics and landscape genomics. The results of this study will deepen our understanding of genomic evolution and population history of *Bergenia* and provide valuable insights for studying the local adaptation mechanisms and conservation strategies. Moreover, our assembled high-quality genome still contains gaps. In recent years, with the development of a multiplatform sequencing technology, an increasing number of telomere-to-telomere (T2T) gap-free genomes have been assembled. T2T genomes play a crucial fundamental role in understanding the structure of new genes, centromeric regions, whole-genome methylation levels, repetitive sequence variations, transposon movement, centromere evolution, and the identification of quantitative trait loci (QTLs) related to important traits [55–58]. Therefore, the quality of our assembled genome still needs to be further improved in future research to provide more references for exploring the functional and regulatory mechanisms of the genome in this species.

Natural selection is the primary force behind species evolution and differentiation. It acts on genetic variation within populations and favors traits that enhance an individual’s fitness in a given environment. As a result, the same species can diverge in different environments through local adaptation, thus reflecting their potential for responding to environmental changes. Comparative genomics suggests that the recent whole-genome duplication event in this species occurred approximately 17.85 Mya (Fig. 2C), corresponding to the transition from the middle Miocene to the Mid-Miocene Climate Optimum (MMCO, approximately 16.9–14.7 Mya). This epochal period marked a remarkable transition from an “ice house” to a “hothouse” climate, during which the MMCO made the climate in most parts of the world warmer, about 5–10°C higher than the current average temperature. Consequently, organisms inhabiting this transitional era faced unprecedented challenges [59, 60]. Furthermore, another older WGD event of this species occurred approximately 85 Mya, marking a crucial turning point from the typical greenhouse climate phase of the Late Cretaceous to a period of global cooling [61]. During this time, the terrestrial climate underwent significant fluctuations, exerting considerable influence on plant species. So, we postulated that the whole-genome duplication event during this period provided the species with additional genetic material, enhancing its evolvability and adaptability to challenging environments. Furthermore, analysis of the expansion and contraction of gene families within the GO/KEGG pathways suggests that these genetic alterations have strengthened the species’ capacity to respond to stress and regulate its growth effectively. Besides, during our initial field investigation, it was intriguing to discover that the wild *B. scopulosa* population extends from the west to the Taibai Mountains (the highest peak of the Qinling Mountains) and to the east to Chang’an district of Xi’an city. This remarkable distribution is further highlighted by the Qinling Mountains in East Asia, which divide northern and southern China and host diverse habitats, including many rare and endangered plant species. The Qinling Mountains’ barrier effect on water vapor creates distinct climates on its northern and southern slopes. While previous studies concentrated on the central region, recent research reveals a temperature pattern over the past 60 years characterized by lower temperatures in the middle and higher temperatures around the periphery, with a predominance of warmer temperatures in the east and south compared to the west and north. As global warming continues, the western and middle sections of the Qinling Mountains have shown the strongest response to rising temperatures [62]. Additionally, research has demonstrated that during the onset of contemporary warming periods, the impact of altitude on vegetation’s response to climate becomes increasingly pronounced [63, 64]. Our population genetics analysis, based on resequencing data, identified 2 distinct geographic groups (West and East) (Fig. 3A–E). This suggests that the population in the Taibai Mountains may be more vulnerable to the effects of climate change. SMC++ analysis backs our hypothesis, revealing that roughly 130,000 years ago, the 2 lineages started to diverge (Fig. 3G), which coincides with the interglacial stage of the late middle Pleistocene epoch, characterized by a frigid climate and widespread glaciation. Notably, the western lineage showed a steeper decline in effective population size than the eastern lineage (Fig. 3G), indicating its greater vulnerability to climatic shifts.

Low temperature, a critical environmental factor, restricts plant growth and distribution [65]. Plants adapt to cold temperatures through a process called cold acclimation, which involves physiological and biochemical adjustments that enhance their tolerance. However, due to the greenhouse effect, global winter temperatures are rising, leading to more frequent temperature fluctuations. This disrupts the cold acclimation process in plants, potentially resulting in earlier deacclimation and increased risk of winter freeze damage [66–69]. As a species resilient to cold temperatures, we have narrowed our research focus to genes linked to cold tolerance. In our study, we utilized 2 distinct methods, LFMMs and RDA, to identify genes involved in genome–environment interactions. Although previous studies have delved into the strengths and limitations of these methods [70, 71], some studies even have suggested that RDA is more effective in detecting adaptive loci compared to LFMMs [71, 72]. While our findings indeed align with this conclusion, highlighting the RDA method’s ability to pinpoint adaptive loci, we also observed that both methods were effective in detecting cold adaptation loci in *B. scopulosa* (Supplementary Table S15). Therefore, we recommend utilizing both LFMMs and RDA to obtain a more comprehensive understanding of core loci.

To be more specific, the GIGANTEA (*GI*) gene is a key regulator, not only in cold adaptation [73] but also in controlling the timing of plant flowering. The qPCR results demonstrated a significant upregulation of *GI* gene expression as the duration of cold acclimation increases. Research has shown that the *GI* gene upregulates CO transcription, leading to a faster flowering process [74, 75]. Based on these findings, we proposed the following scenario: as winter approaches and temperatures drop, the *GI* gene of *B. scopulosa* is significantly upregulated, enabling the species to continue flowering during the cold winter and facilitating a faster completion of its life cycle. Additionally, the membrane-bound *FAD* genes play a pivotal role in maintaining normal plant growth under low-temperature stress [76, 77]. In rice, the expression of *OsFAD8* is significantly induced by low temperatures [78], while the overexpression of the *FAD7* gene enhances cold resistance in transgenic tobacco materials [79]. Our qPCR results for the *BsFAD7* gene align with these findings, highlighting its crucial role of this gene in the adaptation of *B. scopulosa* to low-temperature environments. Furthermore, the qPCR analysis of the core gene *Bsco_038285* from the UDP-glycosyltransferase (UGT) gene family revealed a significant reduction in its expression level under cold treatment (Supplementary Fig. S14). This gene shares homology with *UGT74E2* in *Arabidopsis thaliana*, which is a glucosyltransferase enzyme involved in the glycosylation modification of the hormone auxin Indole-3-butyric Acid (IBA) with an important role in maintaining the dynamic balance of plant hormones [80]. For a deeper exploration of its function (Supplementary Fig. S15), it was integrated into the genome of wild-type *Arabidopsis*, and 3 transgenic lines with high expression levels were chosen for further experimental analysis (Supplementary Figs. S16 and S17). Remarkably, after 2 weeks of cold acclimation, the transgenic *Arabidopsis* lines exhibited significantly longer roots compared to the wild type (WT). However, it is noteworthy that under normal conditions (22°C), there were no significant morphological differences between the WT and *BsUGT74E2*-OEs *Arabidopsis* seedlings (Supplementary Fig. S18). This phenomenon aligns with previous studies [81–83], leading us to speculate that the ectopic expression of this gene in *Arabidopsis* might have a limited impact on regulating IBA homeostasis. When plants encounter stressful conditions, their metabolic resources are redistributed among various physiological pathways, often resulting in stress symptoms such as growth retardation and reduced metabolism. Previous studies have demonstrated that auxin plays a pivotal role in this adaptive response [84]. Upon exposure to cold stress, numerous genes are activated, triggering an increase in various metabolites and protein levels, some of which contribute to a certain degree of cold tolerance. Therefore, we hypothesize that this gene may serve as a crucial component in the cold response mechanism and play a significant role in the adaptive evolution of the species. Future research requires more extensive and profound experiments to further explore its underlying mechanisms. Meanwhile, several studies have indicated that the cold-responsive (*COR*) gene plays a crucial role in enhancing plant cold resistance [85–87]. In our study, we observed a significant increase in the expression level of the *COR* gene as the duration of cold treatment increased, clearly demonstrating its importance in facilitating the adaptation of plants to frigid environments. Furthermore, to gain a deeper understanding of this topic, we opted to utilize several core genes as exemplary representatives for investigating the intricate distribution patterns of allele frequencies within 9 populations (Supplementary Fig. S19). Our comprehensive analysis unveiled that a unique set of rare alleles is predominantly present in the Taibai Mountain populations, belonging to the western lineage, where climatic conditions, characterized by notably lower temperatures, contrast sharply with those of the Guanzhong area in the eastern lineage. This revelation underscores the pivotal role these rare alleles play within the selected genes in facilitating adaptation to the challenging high-altitude environments of the western region. Additionally, in *FAD7*, the varying homozygous and heterozygous states of these differential alleles between the 2 lineages may also be a factor contributing to the increased vulnerability of the western lineage. In summary, a series of core cold-adaptation genes collectively shape the local adaptation pattern of this species. However, with the frequent occurrence of extreme climate events, this endangered cold-tolerant species is also facing challenges brought about by climate change. Consequently, safeguarding the rare alleles within more core genes and identifying those related to cold tolerance will provide invaluable genetic resources for the future conservation efforts of this species.

As historical climate fluctuations intertwine with human influence on the environment, global climate changes fragment species habitats, reducing gene flow and adaptive genetic variation, potentially leading to local extinctions, especially in small, endangered populations [88]. Our research aims not only to clarify the connection between local adaptation mechanisms and environmental factors but also to utilize genomic adaptive information to predict population vulnerability in the face of climate change. Wild *B. scopulosa*, rich in bergenin and highly valued in traditional medicine, is currently overexploited by local farmers, leading to a significant population decline. In our study, we observed a notable increase in the effective population since the past 25 years in the eastern lineage, possibly due to the increased awareness of the ecological diversity present in the Qinling Mountains. This heightened awareness has prompted the implementation of government-led ecological conservation plans, strengthening the protection of rare and endangered species in the region. Furthermore, our research has revealed that the Taibai Mountain population in the western lineage exhibits a higher genetic offset (Fig. 5), indicating a heightened vulnerability to future environmental shifts. Climate change poses a global challenge, and the Chinese government has taken a series of actions to conserve biodiversity, including establishing a network of nature reserves, reinforcing species protection, and restoring habitats. Given the increased vulnerability of the Taibai Mountain populations and their valuable genetic resources adapted to cold climates, it is imperative to prioritize and implement targeted conservation strategies for these populations. For the entire population, the primary objective is to expand the population size while maintaining current numbers. First, it is essential to enhance the protection and monitoring of its native habitat and plants by establishing conservation sites dedicated to this species and preserving its genetic diversity *in situ*. Additionally, based on our previous seed germination experiments, we have found that this species exhibits higher germination rates in laboratory conditions compared to the lower rates observed in the wild. Therefore, it is advisable to collect seeds of this species and engage in artificial cultivation for *ex situ* conservation. This will help maintain sufficient genetic diversity and maximize the environmental adaptability of this species. Finally, considering the overwhelming demand for traditional Chinese medicine, it is urgent to establish a swift propagation system for this rare and endangered species to ensure its sustainable utilization.

## Materials and Methods

### Plant materials and genome sequencing

The natural plants of *B. scopulosa* were collected from a single plant in the Qinling Mountains, Shaanxi Province, China (N33°57′42″, E109°3′37″), to ensure genetic diversity and representativeness. Total genomic DNA was obtained using a modified method of SDS-CTAB and sequenced on an Illumina NovaSeq 6000 sequencing system (RRID:SCR_016387) for short-read sequencing and the PacBio SEQUEL2 platform (RRID:SCR_017990; Pacific Biosciences) for long-read sequencing. A fresh young plant was used to create the Hi-C libraries. For each library, the chromatin was fixed with formaldehyde in the nucleus, and the cross-linked DNA was digested using the restriction enzyme DpnII. Hi-C sequencing libraries were amplified by PCR (12–14 cycles) and sequenced on an Illumina NovaSeq 6000 sequencing system (RRID:SCR_016387).

### Genome size estimate and assembly

To estimate the genome size of *B. scopulosa*, clean Illumina reads were used to calculate the 17-*k*-mer distribution using SOAPdenovo (RRID:SCR_010752) [89]. The estimation of genome size was computed using the following formula: G = *k*-mer_number/*k*-mer_depth [90]. Long clean reads of PacBio were assembled using Hifiasm v0.15.4-r343 (RRID:SCR_021069) [91]. The primary contigs were filtered and error-corrected with Nextpolish (RRID:SCR_025232) [92] using Illumina short reads. For Hi-C library preparation, we employed the alignment strategy of HiC-Pro (RRID:SCR_017643) [93] and utilized bowtie2 (RRID:SCR_016368) [94] for alignment. We applied the LACHESIS (RRID:SCR_017644) [95] algorithm with a bottom-up hierarchical clustering method to cluster scaffolds into 17 chromosome groups. After Hi-C–assisted assembly, the sketch contigs/scaffolds were anchored into pseudo-chromosomes. Ultimately, we evaluated the integrity of the *B. scopulosa* genome using the BUSCO v5.1.2 (RRID:SCR_015008) [96].

### Genome annotation

Homology-based annotation of the *B. scopulosa* genome was performed using a collection of protein-coding genes from 7 plant species: *A. thaliana, Beta vulgaris, Salvia bowleyana, K. fedtschenkoi, Oryza sativa, Rhodiola crenulate*, and *Solanum lycopersicum*. Repeat elements were annotated using RepeatModeler v2.0.1 (RRID:SCR_015027) [97], which primarily utilizes 2 programs, Recon (RRID:SCR_021170) [98] and RepeatScout (RRID:SCR_014653) [99]. For genome structure annotation, we used the Augustus package (RRID:SCR_008417) [100] within the Braker v2.1.5 (RRID:SCR_018964) [101] for *de novo* gene prediction. Additionally, the MAKER v3.01.03 pipeline (RRID:SCR_005309) [102] was chosen to predict protein-coding gene models in *B. scopulosa*. For gene function annotation, Blastp (RRID:SCR_001010) was employed with an E-value threshold of ≤1e^−5^ to align the annotated genes with eggNOG, GO, COG, and KEGG databases [103–105]. For noncoding RNA annotation, tRNAscan-SE v2.0 (RRID:SCR_008637) [106] was used to identify transfer RNA sequences, while Blastn (RRID:SCR_001598) was employed to retrieve specific ribosomal RNA sequences. INFERNAL v1.1.3 (RRID:SCR_011809) [107] based on the Rfam (RRID:SCR_007891) [108] covariance models was used to predict microRNA and small nuclear RNA sequences in the genome.

### Comparative genomic and evolutionary analyses

Nine high-quality genomic data were selected from *A. thaliana, O. sativa, Amborella trichopoda, K. fedtschenkoi, B. vulgari, Paeonia ostii, T. polyphylla, P. ludlowii*, and *P. suffruticosa* using OrthoFinder v 2.5.2 (RRID:SCR_017118) [109] to perform gene family clustering. CAFE5 (RRID:SCR_005983) [110] was used to estimate gene family expansion and contraction. To construct the phylogenetic tree, Muscle v3.8.1551 (RRID:SCR_011812) [111] was used to align single-copy genes, followed by RAxML v8.2.12 (RRID:SCR_006086) [112] to build the phylogenetic tree based on the maximum likelihood method. With the assistance of fossil records to determine the evolutionary time scale, we extracted the 4-fold degenerate sites (4DTv) of each gene family and calculated the divergence time between species using the MCMCtree module in PAML v4.9 (RRID:SCR_014932) [113]. Additionally, Ks values between paralogous gene pairs of *B. scopulosa* were calculated using the yn00 model. MCscanX (RRID:SCR_022067) [114] was used to identify collinear regions among species, and the HKY model was used to estimate the substitution rate between species, aiding in the inference of WGD that may have occurred in the *B. scopulosa* genome.

### Genome resequencing and variant calling

A total of 37 individuals were collected from 9 natural populations spanning the entire distribution of the species. To ensure high-quality and completeness of sequencing, all samples were extracted with the Plant DNA Kit for DNA extraction (Omega Bio-tek), and the whole-genome paired-end sequencing was generated using the DNBSEQ-T7 platform (RRID:SCR_017981), with an average sequencing depth of at least 20×. To improve data quality, the initial processing involved filtering low-quality bases, adapter sequences, duplicates, and contaminated reads using fastp v0.23.1 (RRID:SCR_016962) [115]. The clean reads were subsequently aligned to the assembled *B. scopulosa* genome via BWA v0.7.17-r1188 (RRID:SCR_010910) [116] and subsequently were sorted and converted to BAM format using SAMtools v1.16 (RRID:SCR_002105) [117]. The duplicate reads were removed with Sambamba v0.8.2 (RRID:SCR_024328) [118]. SNPs were called from the HaplotypeCaller program in GATK v4.1.4.1 (RRID:SCR_001876) [119]. Raw SNPs were filtered using VariantFiltration with filters “QD < 2.0 || MQ < 40.0 || FS > 60.0 || SOR > 3.0 || MQRankSum < -12.5 || ReadPosRankSum < -8.0.” The final filtering of SNPs was accomplished using VCFtools v0.1.16 (RRID:SCR_001235) [120], with parameters “maf 0.05 max-alleles 2 min-alleles 2 minGQ 20 min-meanDP 5 max-missing 0.8.”

### Population genomics analyses

An SNP-based ML phylogenetic tree was constructed using RAxML-NG v1.1 (RRID:SCR_022066) [121] with the GTRGAMMA substitution model and visualized by Figtree v1.4.3 (RRID:SCR_008515). The population genetic structure of *B. scopulosa* was inferred by applying the block relaxation algorithm in ADMIXTURE v1.3.0 (RRID:SCR_001263) [122]. The parameter *K* ranged from 2 to 10. PCA calculations were performed on the bed file generated by PLINK v1.90b6.4 (RRID:SCR_001757) [123], and the results were visualized using the ggplot2 package (RRID:SCR_014601) in R. Following population-based SNP detection in *B. scopulosa*, we employed a sliding-window strategy, utilizing 100-kb windows sliding in 10-kb increments. This allowed us to assess nucleotide diversity (*π*) and genetic differentiation (*F*_ST_) between different lineages using VCFtools. Selected candidate regions for the western and eastern groups of *B. scopulosa* were identified by taking the intersection of 5% left and right tails of the empirical log_10_*_π_*_-ratio_ (*π_*east/*π_*west) distribution and the 5% right tail of the empirical *F*_ST_ distribution. Subsequently, KEGG and GO annotations were performed on the genes located in these regions. Finally, the effective population size was estimated through demographic history inference using PSMC (RRID:SCR_017229) [124] and SMC++ [125], assuming a per-generation mutation rate of 2.5e-8 and a generation time of 5 years.

### Identification of environmental-related genetic variations in *B. scopulosa*

To minimize false-positive results, we selected 8,813,357 SNPs with a minor allele frequency greater than 0.1 for further analysis. To evaluate the influence of environmental variables on population differentiation of *B. scopulosa* and gain insights into the patterns of allele frequency variation along environmental gradients, we obtained 19 climate variables from the Worldclim in raster files (.asc) with a spatial resolution of 2.5 arcmin using ArcGIS v10.8 (RRID:SCR_011081; ESRI). After evaluating the importance ranking of 19 climate variables using the GF function in the R package “gradientForest” [126], we selected 5 variables (BIO3, BIO4, BIO15, BIO18, and BIO19) with correlation coefficients | *r* | ≤ 0.75 for further analysis. We then employed 2 approaches to identify the SNPs associated with climate factors. Initially, we used the “lfmm” function in R package LEA (RRID:SCR_022020) [127] to execute a univariate LFMM [128] for detecting allele frequency associations with major environmental variables. Based on the optimal genetic grouping inferred by ADMIXTURE v1.3.0, we conducted 5 independent Markov chain Monte Carlo runs using 500 iterations as burn-in followed by 1,000 iterations and kept SNPs with a false discovery rate correction of *P* < 0.05. Additionally, we used a multivariate landscape genomics method known as RDA [129] to explore the correlation with climate factors. Outlier SNPs, defined as those with at least 3 times as many putative explanatory variables as examined, were excluded. This helped us pinpoint genetic variations tightly linked to the multivariate environmental axis. The overlapping results from both methods were considered core “adaptive loci.”

### Real-time qPCR validation

To investigate the genes associated with the potential core sites for cold adaptation, we utilized sterile seedlings of *B. scopulosa* and subjected them to a cold acclimation treatment at 4°C for varying durations of 0, 6, 12, 24, and 48 hours. The total RNA was extracted using a FastPure Universal Plant Total RNA Isolation Kit (Vazyme). Complementary DNA was obtained using Hifair Ⅲ 1st Strand cDNA Synthesis SuperMix, and the qPCR reactions were carried out using a Hieff qPCR SYBR Green Master Mix (No Rox) (Yeasen Biotechnology) performed on the FQD-96C real-time detection system (Boer Technology). The *Bsco_actin* gene served as an internal reference, and gene-specific primers were utilized for the reactions (Supplementary Table S16). Each reaction was technically repeated 3 times for accuracy and reproducibility.

### Analysis of gene families

To identify candidate *BsFAD* genes in the *B. scopulosa* genome, we used protein sequences of the FAD from *Arabidopsis*, wheat, rice, and soybean as queries in BLAST searches (RRID:SCR_004870). The obtained sequences were then analyzed using Pfam (RRID:SCR_004726) and the SMART website (RRID:SCR_005026) for structural predictions. The phylogenetic tree was constructed using the LG+G model in IQ-TREE (RRID:SCR_017254) [130] through the ML method with 1,000 bootstraps for inferring evolutionary relationships.

### Transcriptome sequencing and analysis

Total RNA was extracted from the roots, leaves, flowers, and buds from 11 fresh samples of *B. scopulosa* from the XYX population in the Qinling Mountains, Shaanxi Province, China (N33°57′42″, E109°3′37″). RNA libraries were constructed for each sample and sequenced on an Illumina NovaSeq 6000 sequencing system (RRID:SCR_016387). Clean reads were aligned to the reference genome using HISAT2 v2.2.1 (RRID:SCR_015530) [131]. The R script “FeatureCounts” (RRID:SCR_012919) was used to calculate the reads counts matrix, which was then converted into FPKM and TPM values. The differential gene expression analysis was conducted using run_DE_analysis.pl script in Trinity (RRID:SCR_013048) [132].

### Genomic offset of *B. scopulosa*

We used the current genotype–climate relationship and identified climate-associated genetic loci to forecast the vulnerability (genetic offset) of the *B. scopulosa* based on 19 future climatic variables (2061–2080) from the WorldClim CMIP6 dataset [133] with a resolution of 2.5 arcmin of 4 climate models (CMCC-ESM2, EC-Earth3-Veg, GISS-E2-1-G, and MIROC6). Each future environmental dataset contains 2 shared socioeconomic pathways: ssp126 and ssp585. In addition, the analysis “gradientForests” [126] in R predicts genetic offset under future climate conditions across the range of *B. scopulosa*. Euclidean distance was calculated between the current and each future climate scenario to represent genetic disparity. We then averaged the values across the 4 future climate scenarios. A higher value indicates greater genomic vulnerability of *B. scopulosa* [134].

## Additional Files

**Supplementary Fig. S1**. *K*-mer analysis of the *B. scopulosa* genome based on Illumina clean data.

**Supplementary Fig. S2**. Hi-C assisted assembly of *B. scopulosa* pseudochromosomes. Heatmap showing Hi-C interactions under a resolution of 500 kb.

**Supplementary Fig. S3**. Genome assembly completeness evaluated based on different BUSCO groups.

**Supplementary Fig. S4**. Kimura distance-based copy divergence analysis of transposable elements in the *B. scopulosa* genome.

**Supplementary Fig. S5**. Genome duplication in *B. scopulosa*.

**Supplementary Fig. S6**. Sample geographic distribution for *B. scopulosa*.

**Supplementary Fig. S7**. The distribution of π along the chromosomes among the lineages of east and west, respectively.

**Supplementary Fig. S8**. The distribution of *F*_ST_ values (a) and the log_10_π ratios (*π*_east/*π*_west) along the chromosomes in *B. scopulosa* (b). Data points located to the left and right of the left and right vertical dashed lines, respectively (corresponding to the 5% left and right tails of the empirical log_10_π ratio distribution), and above the horizontal dashed line (the 5% right tail of the empirical *F*_ST_ distribution) were identified as selected regions for lineages of east (red points) and west (blue points) (c).

**Supplementary Fig. S9**. KEGG analysis of top 5% genes under selection. Overrepresented gene ontology terms were identified using *P* < 0.05.

**Supplementary Fig. S10**. The graphs show the importance ranking of 19 environmental variables based on gradient forest analysis at SNPs (below the diagonal) and the Pearson correlation coefficient between these variables (above the diagonal). The asterisk (*) represents 5 highly ranked and unrelated environmental variables (Pearson’s | *r* | ≤ 0.75).

**Supplementary Fig. S11**. KEGG enrichment analysis of genes underlying the outliers using a latent factor mixed model. Overrepresented gene ontology terms were identified using *P* < 0.05.

**Supplementary Fig. S12**. Redundancy analysis of 5 selected environmental factor response patterns in genetic variation of *B. scopulosa*.

**Supplementary Fig. S13**. KEGG enrichment analysis of genes underlying the outliers from RDA. Overrepresented gene ontology terms were identified using *P* < 0.05.

**Supplementary Fig. S14**. The mRNA relative expression levels at 0, 6, 12, 24, and 48 hours under cold treatment in *BsUGT74E2* from sterile seedling of *B. scopulosa*.

**Supplementary Fig. S15**. Subcellular localization of *BsUGT74E2* (*Bsco_038285*) protein in tobacco epidermal cells.

**Supplementary Fig. S16**. Identification of transgenic *Arabidopsis* positive seedlings by PCR. 1–10: L1, L3, L7, L9, L11, L12, L19, L20, L24, L28 transgenic *Arabidopsis*.

**Supplementary Fig. S17**. The relative expression of *BsUGT74E2* in a transgenic *A. thaliana* strain.

**Supplementary Fig. S18**. Effects of *BsUGT74E2* overexpression on *Arabidopsis* seedling root length.

**Supplementary Fig. S19**. Allele frequencies of candidate adaptive SNPs: (a) *FAD7*, chr6_12100635, (b) *COR413pm2*, chr14_24427933, (c) *MYC2*, chr15_22548565, and (d) *CRF2*, chr14_21366166 associated with BIO3 and BIO4 across the 9 populations. Colors mean different alleles. N means missing alleles at leading SNP.

**Supplementary Table S1**. Estimation of genome size of *B. scopulosa*.

**Supplementary Table S2**. Summary of sequencing data of *B. scopulosa*.

**Supplementary Table S3**. Statistic of *B. scopulosa* genome assembly.

**Supplementary Table S4**. Chromosomes length of *B. scopulosa* using Hi-C reads.

**Supplementary Table S5**. Validation of genome assembly using BUSCO method with 3 databases.

**Supplementary Table S6**. The number of genes annotated for function using various methods.

**Supplementary Table S7**. Statistical analysis of noncoding RNAs in *B. scopulosa*.

**Supplementary Table S8**. Repetitive element annotations in the *B. scopulosa*.

**Supplementary Table S9**. Gene Ontology (GO) enrichment analysis of the significant expanded genes.

**Supplementary Table S10**. KEGG enrichment analysis of the significant expanded genes.

**Supplementary Table S11**. Gene Ontology (GO) enrichment analysis of the significant contracted genes.

**Supplementary Table S12**. KEGG enrichment analysis of the contracted genes.

**Supplementary Table S13**. Sample information and genome sequencing characteristics of *B. scopulosa*.

**Supplementary Table S14**. KEGG analysis of genomic regions exhibited high differentiation and reduced diversity between east lineage and west lineage.

**Supplementary Table S15**. Candidates under the outliers from genome–environment associations.

**Supplementary Table S16**. Sequence of primers used for the qRT-PCR test under cold acclimation.

## Abbreviations

BUSCO: Benchmarking Universal Single‐Copy Orthologs; CV: cross-validation; GEA: genotype–environment association; GO: Gene Ontology; GWAS: genome-wide association studies (GWAS); KEGG: Kyoto Encyclopedia of Genes and Genomes; LFMM: latent factor mixed model; LTR: long terminal repeat; ML: maximum likelihood; MMCO: Mid-Miocene Climate Optimum; Mya: million years ago; NGS: next-generation sequencing; PCA: principal component analysis; PSMC: pairwise sequential Markovian coalescent; qPCR: quantitative PCR; QTL: quantitative trait loci; QTL: quantitative trait locus; RDA: redundancy analysis; SNP: single-nucleotide polymorphism; T2T: telomere-to-telomere; UGT: UDP-glycosyltransferase; WGD: whole-genome duplication; WGS: whole-genome sequencing; WT: wild type.

## Supplementary Material

giae091_Supplementary_Files

giae091_GIGA-D-24-00141_Original_Submission

giae091_GIGA-D-24-00141_Revision_1

giae091_GIGA-D-24-00141_Revision_2

giae091_Response_to_Reviewer_Comments_Original_Submission

giae091_Response_to_Reviewer_Comments_Revision_1

giae091_Reviewer_1_Report_Original_Submission Shengxiong Huang -- 6/2/2024

giae091_Reviewer_1_Report_Revision_1 Shengxiong Huang -- 9/5/2024

giae091_Reviewer_2_Report_Original_Submission Yongfeng Zhou -- 6/11/2024

## Data Availability

The raw genomic sequence and RNA-seq data of *B. scopulosa* generated by this study were deposited into the NGDC (National Genomics Data Center) database under accession number PRJCA025818 and NCBI under BioProject ID PRJNA1110036. The genome assembly, annotations, and other supporting data are available via the *GigaScience* database, GigaDB [135].
