# Supplementary material for: The chromosome-level genome assembly of an endangered herb Bergenia scopulosa provides insights into local adaptation and genomic vulnerability under climate change
Source: Gigascience. 2024 Nov 28;13:giae091. doi: 10.1093/gigascience/giae091 (PMC11604060; doi:10.1093/gigascience/giae091)
Supplement: giae091_Supplementary_Files [file giae091_supplementary_files.zip › supplementary_material_figures_GigaScience-revised2.docx]

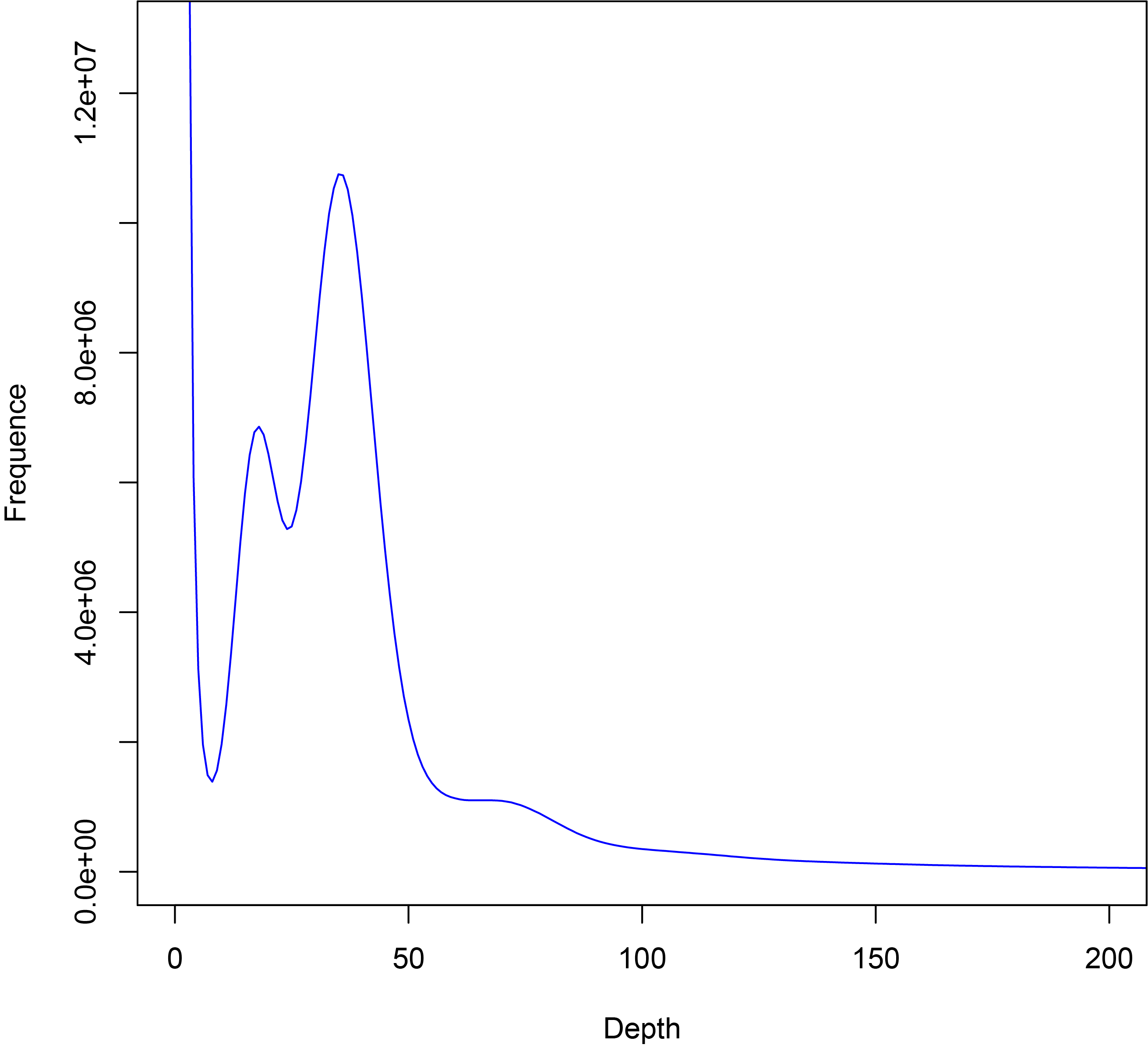


Fig. S1. K-mer analysis of the *B. scopulosa* genome based on Illumina clean data.


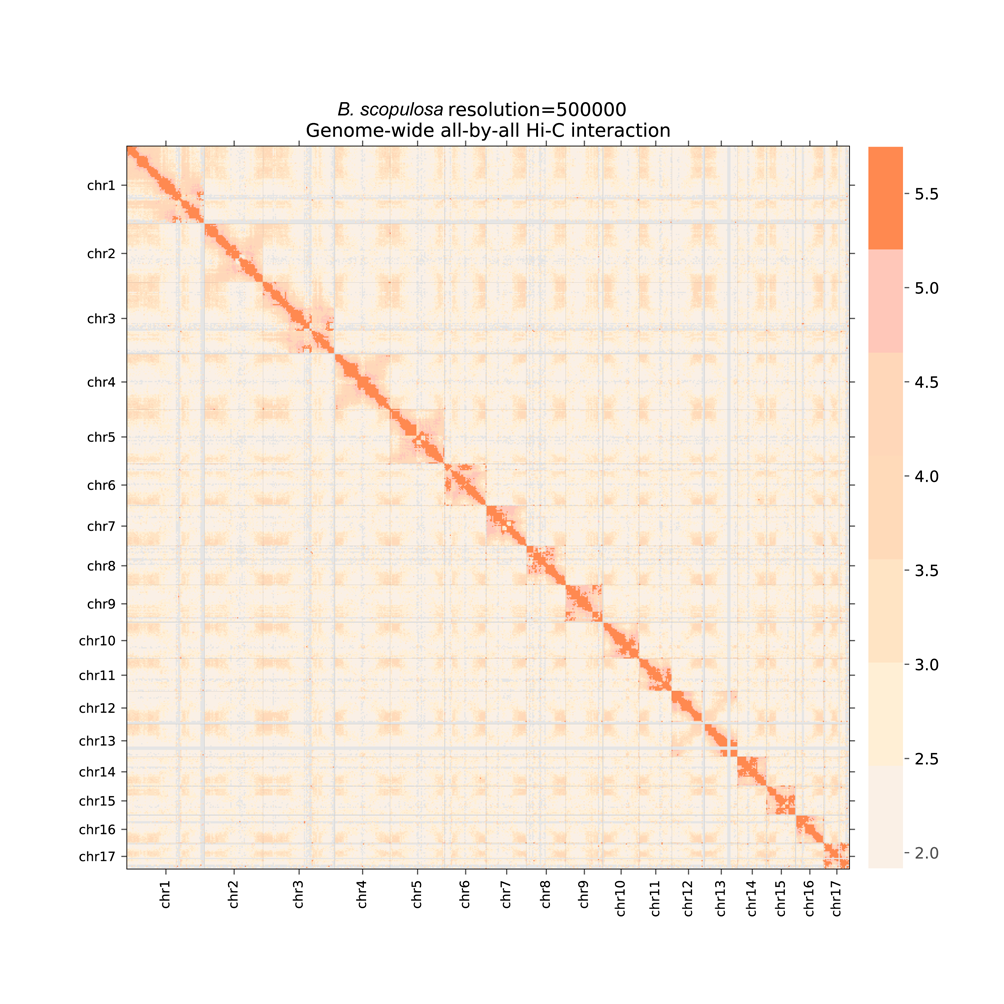


Fig. S2. Hi-C assisted assembly of *B. scopulosa* pseudochromosomes. Heatmap showing Hi-C interactions under a resolution of 500 kb.


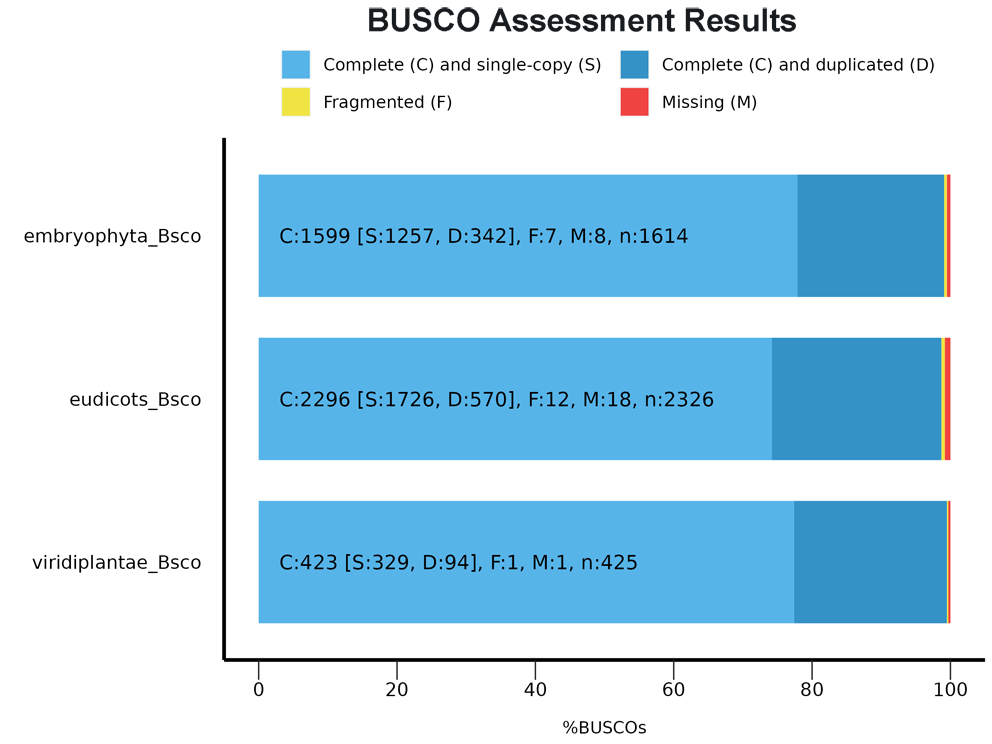


Fig. S3. Genome assembly completeness evaluated based on different BUSCO groups.


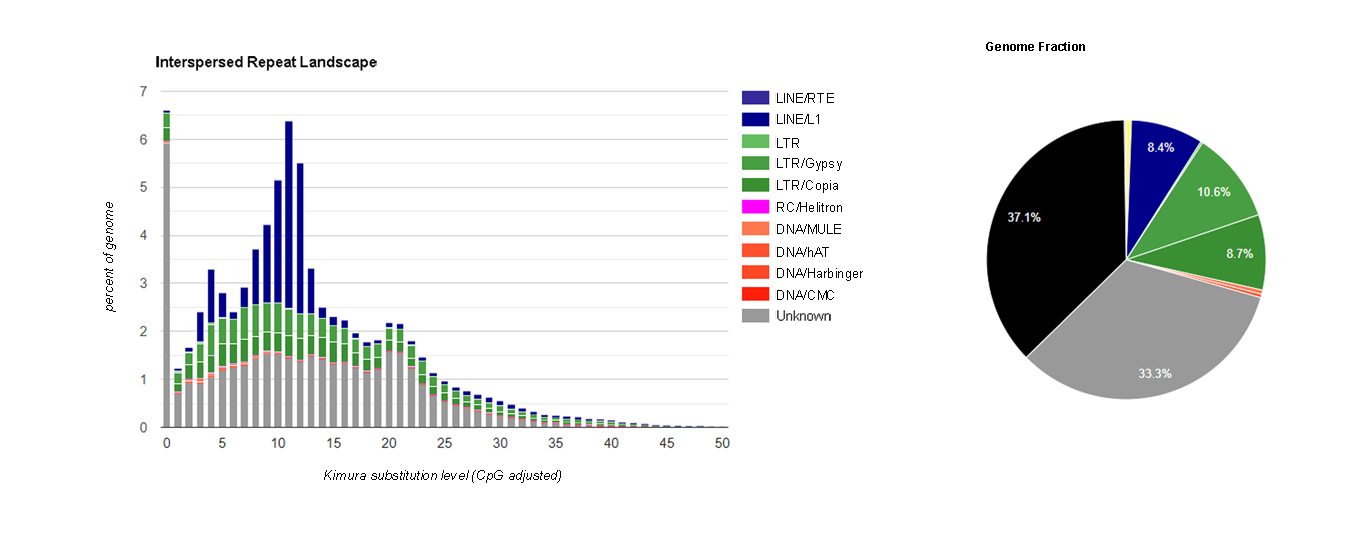


Fig. S4. Kimura distance-based copy divergence analysis of transposable elements in *B. scopulosa* genome.


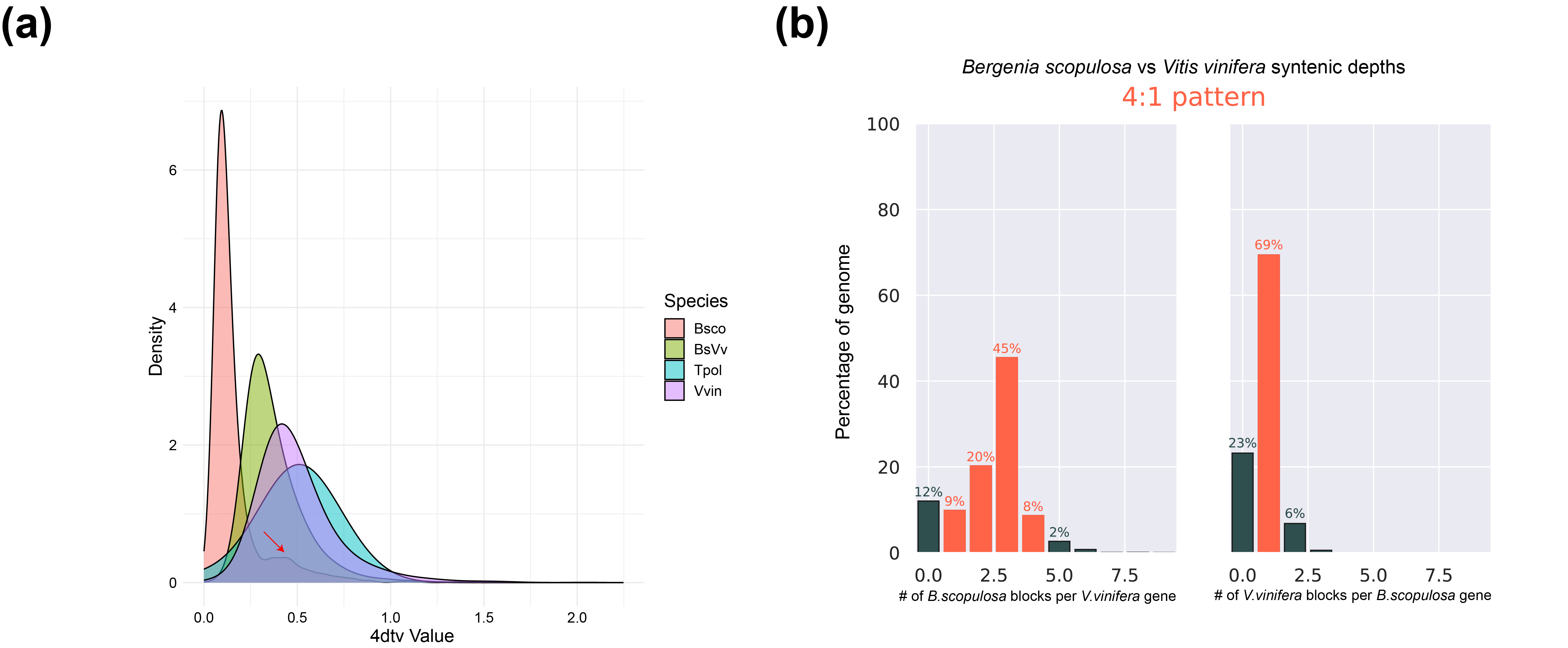


Fig. S5 Genome duplication in *B. scopulosa*. **(a)** Distribution of the four-fold synonymous third-codon transversion rate (4DTv) for paralogous genes within the genomes of *B. scopulosa*, *Tiarella polyphylla* and *Vitis vinifera* and orthologous genes between those of *B. scopulosa* and *V. vinifera*. The peak of the gamma WGD event shared by *B. scopulosa*, *V. vinifera* and *T. polyphylla* is indicated by the red arrow. Bsco stands for *B. scopulosa*, BsVv stands for *B. scopulosa***-***V. vinifera*, Tpol stands for *T. polyphylla* and Vvin stands for *V. vinifera*. **(b)** Syntenic depths in *B. scopulosa* vs. *V. vinifera* genome comparison**.**


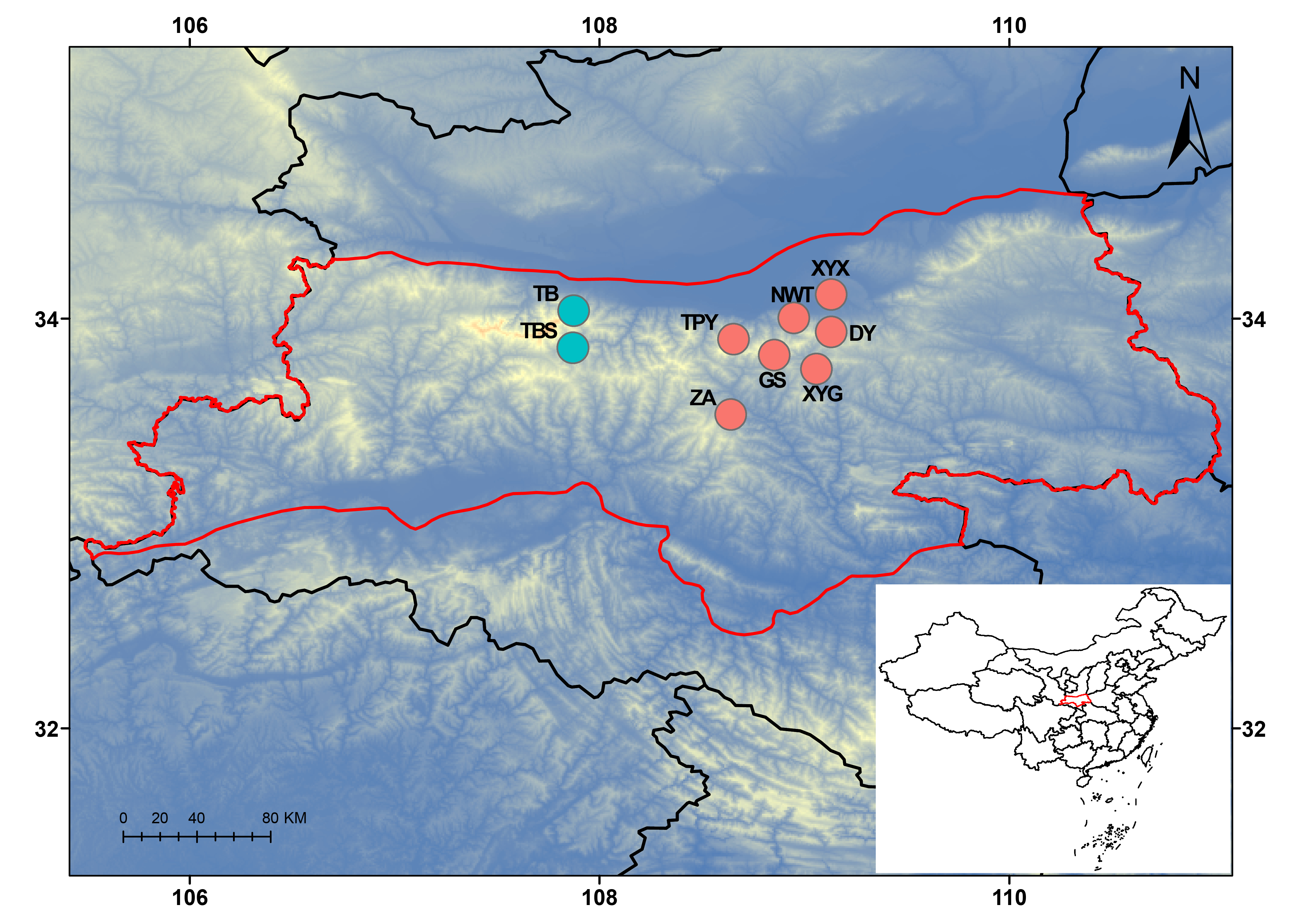


Fig. S6. Samples geographic distribution for *B. scopulosa*. The red circle shows the Qinling Mountains region within Shaanxi Province, China. Blue dot stands for West group, red dots stand for East group.


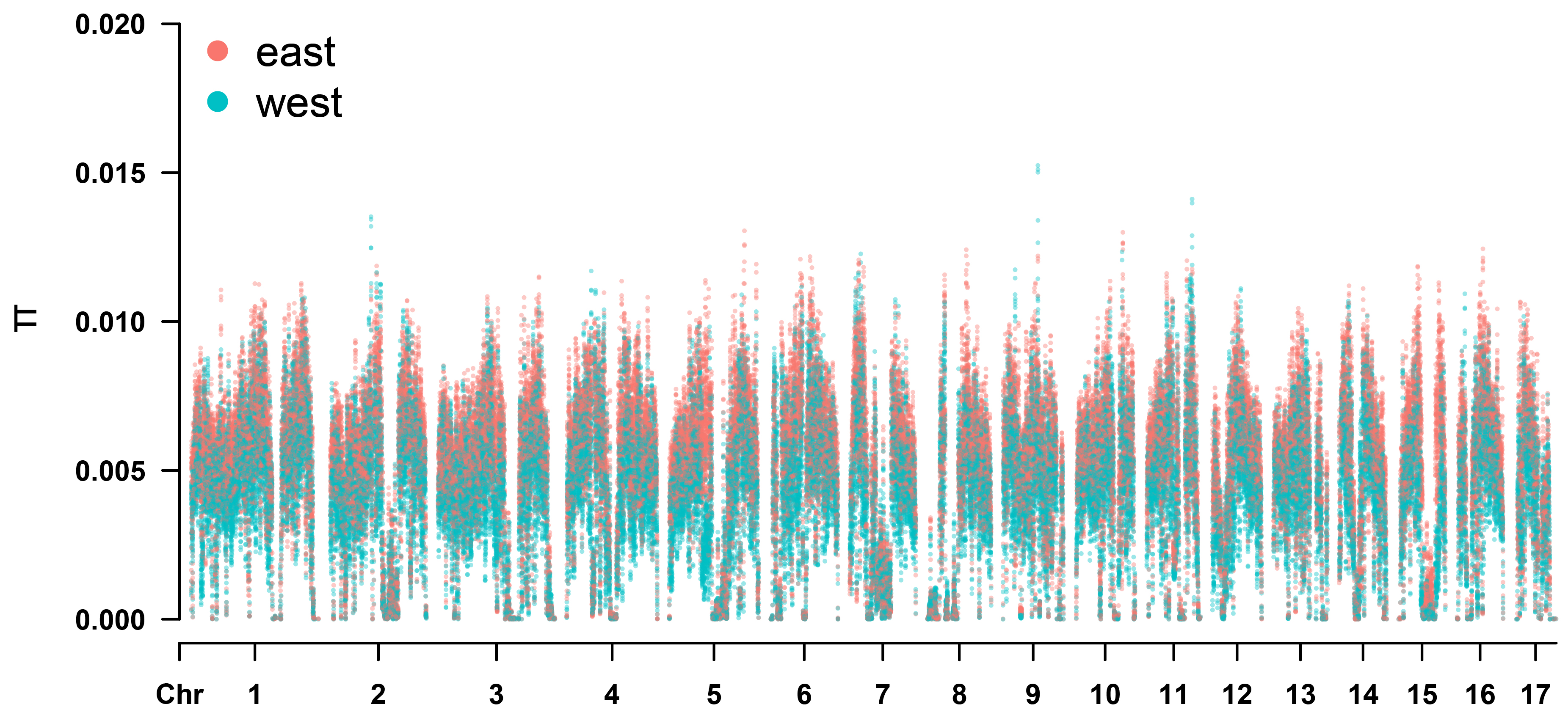


Fig. S7. The distribution of *π* along the chromosomes among the lineages of east and west, respectively.


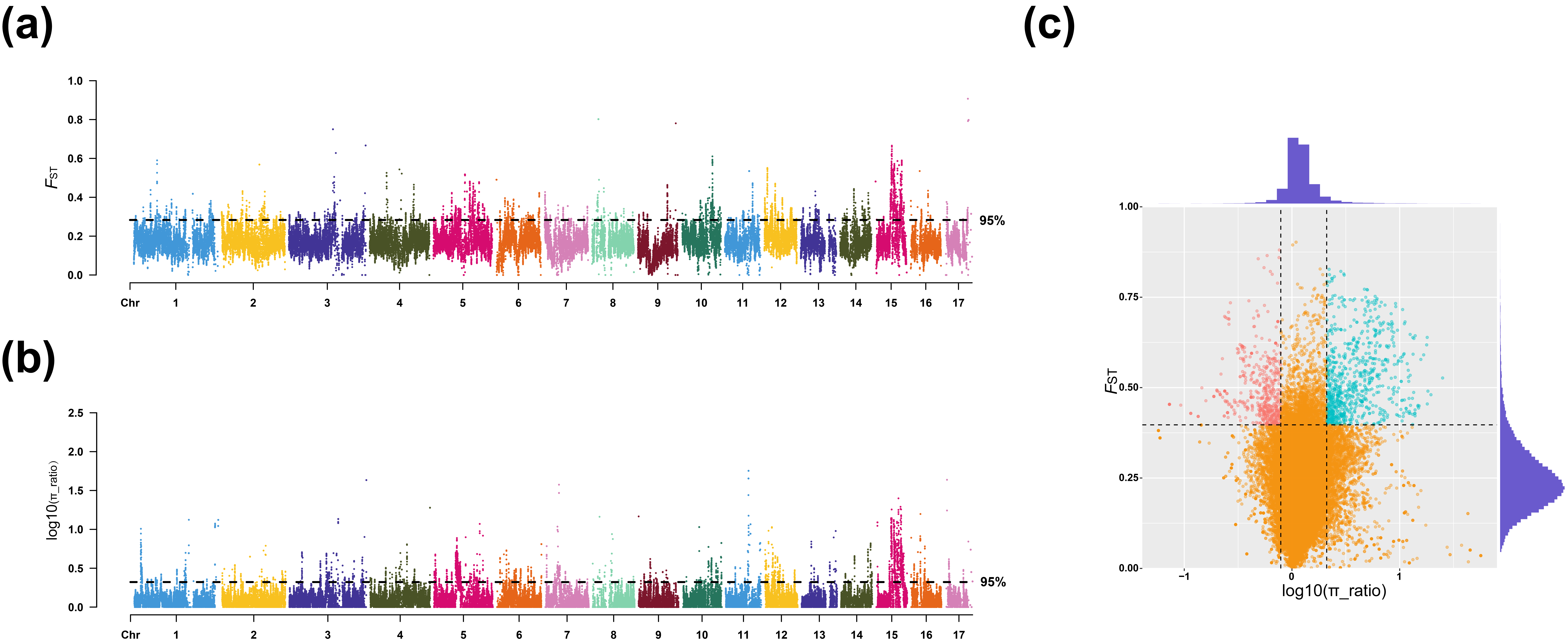


Fig. S8. The distribution of *F*_ST_ values **(a)** and the log10π ratios (*π*_east/*π*_west) along the chromosomes in *B. scopulosa* **(b)**. Data points located to the left and right of the left and right vertical dashed lines, respectively (corresponding to the 5% left and right tails of the empirical log10π ratios distribution), and above the horizontal dashed line (the 5% right tail of the empirical *F*_ST_ distribution) were identified as selected regions for lineages of east (red points) and west (blue points) **(c)**.


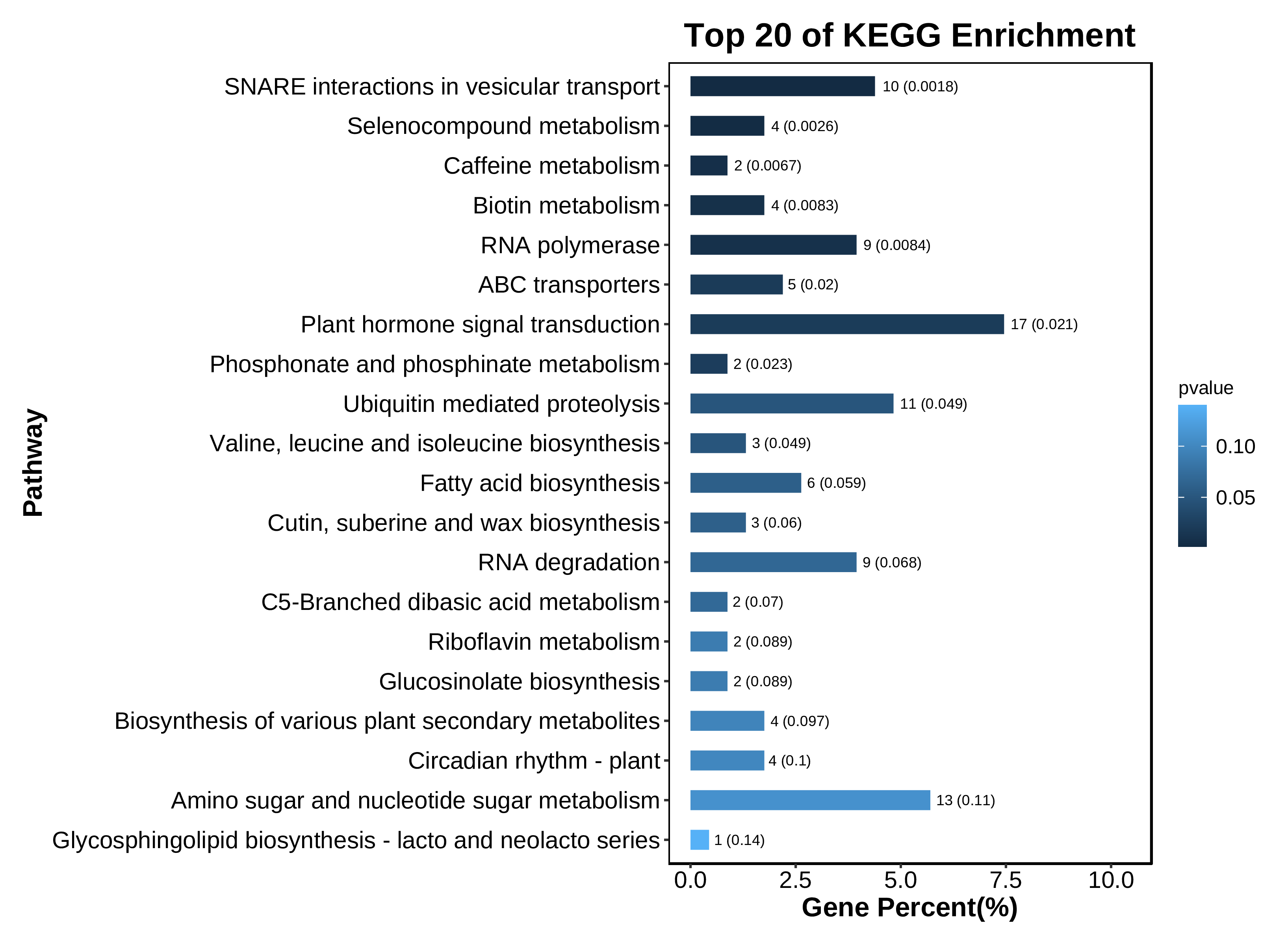


Fig. S9. KEGG analysis of top 5% genes under selection. Overrepresented gene ontology terms were identified using a *P*value < 0.05.


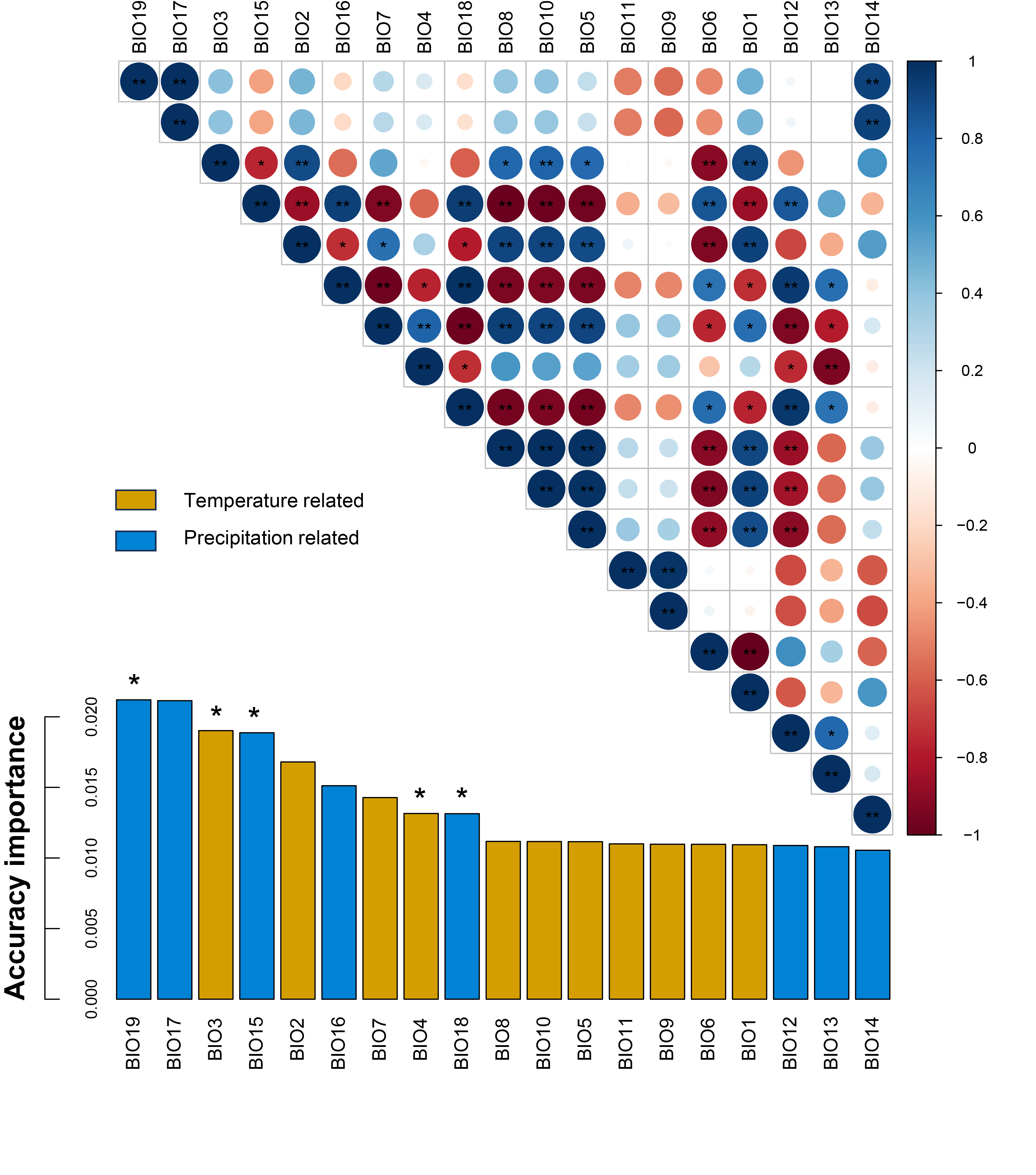


Fig. S10. The graphs show the importance ranking of 19 environmental variables based on gradient forest analysis at SNPs (below the diagonal), and the Pearson correlation coefficient between these variables (above the diagonal). The asterisk (*) represents five highly ranked and unrelated environmental variables (Pearson’s | *r* | ≤ 0.75).


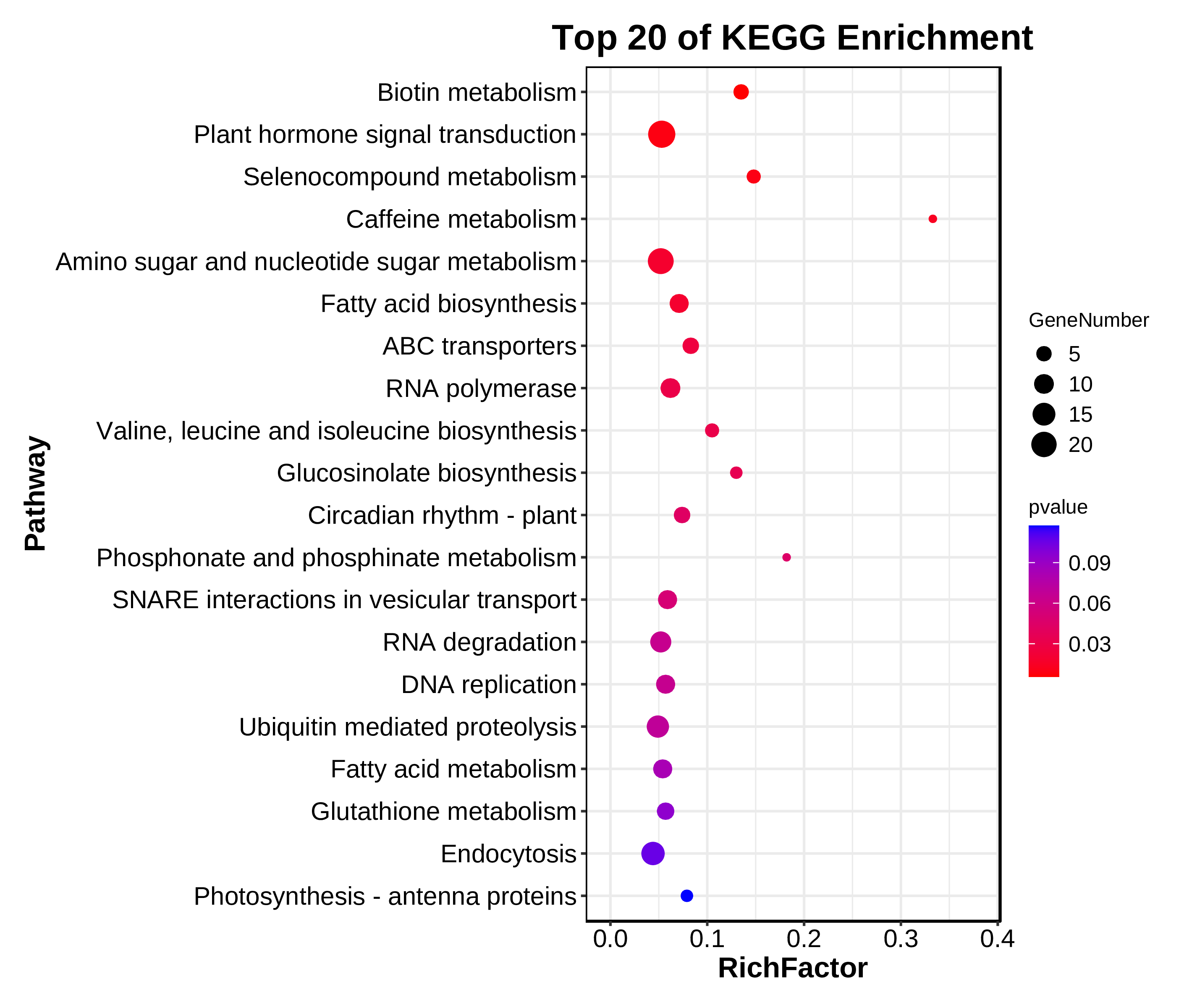


Fig. S11. KEGG enrichment analysis of genes underlying the outliers using latent factor mixed model. Overrepresented gene ontology terms were identified using a *P*value < 0.05.


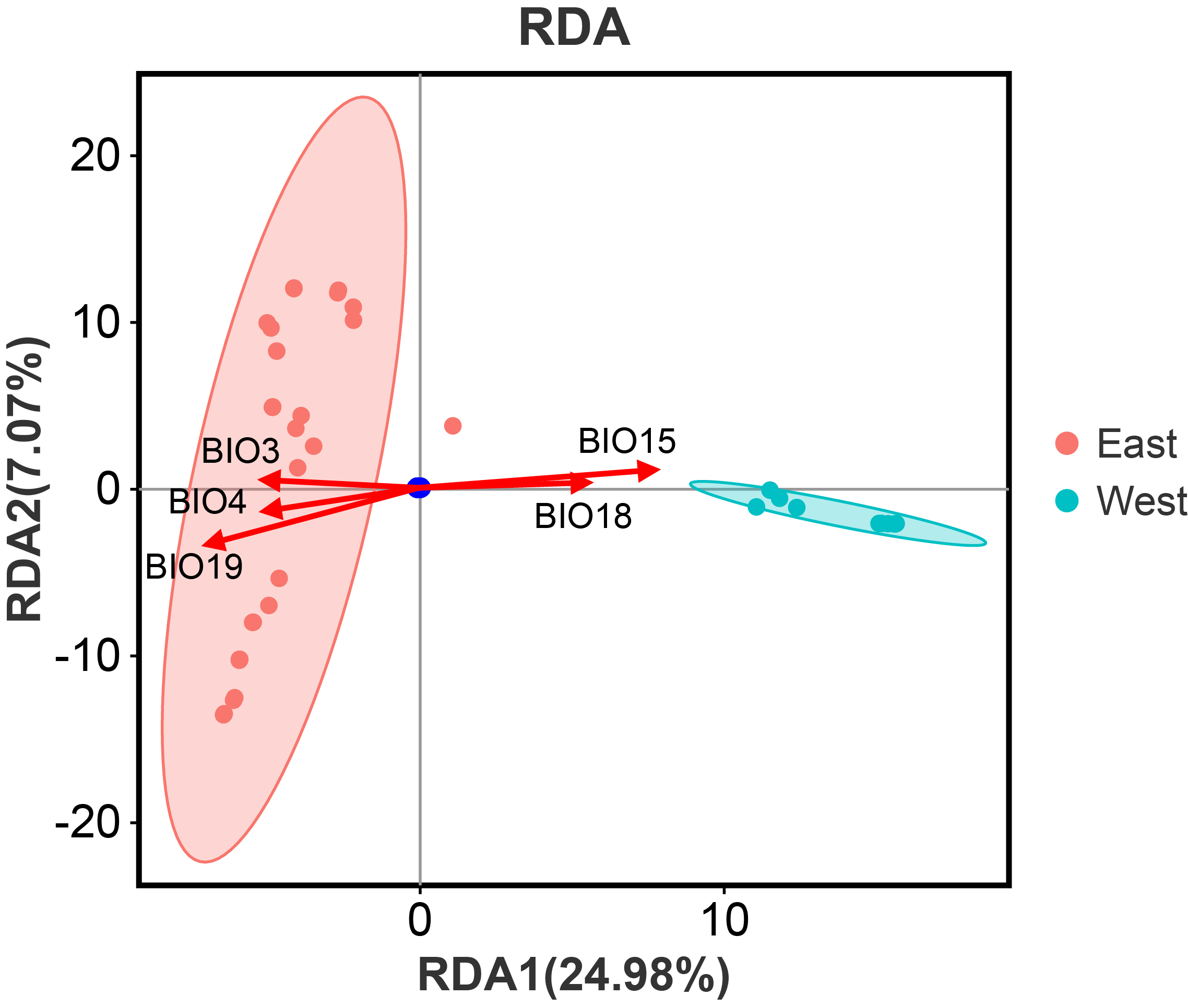


Fig. S12. Redundancy analysis of five selected environmental factors response patterns in genetic variation of *B. scopulosa*.


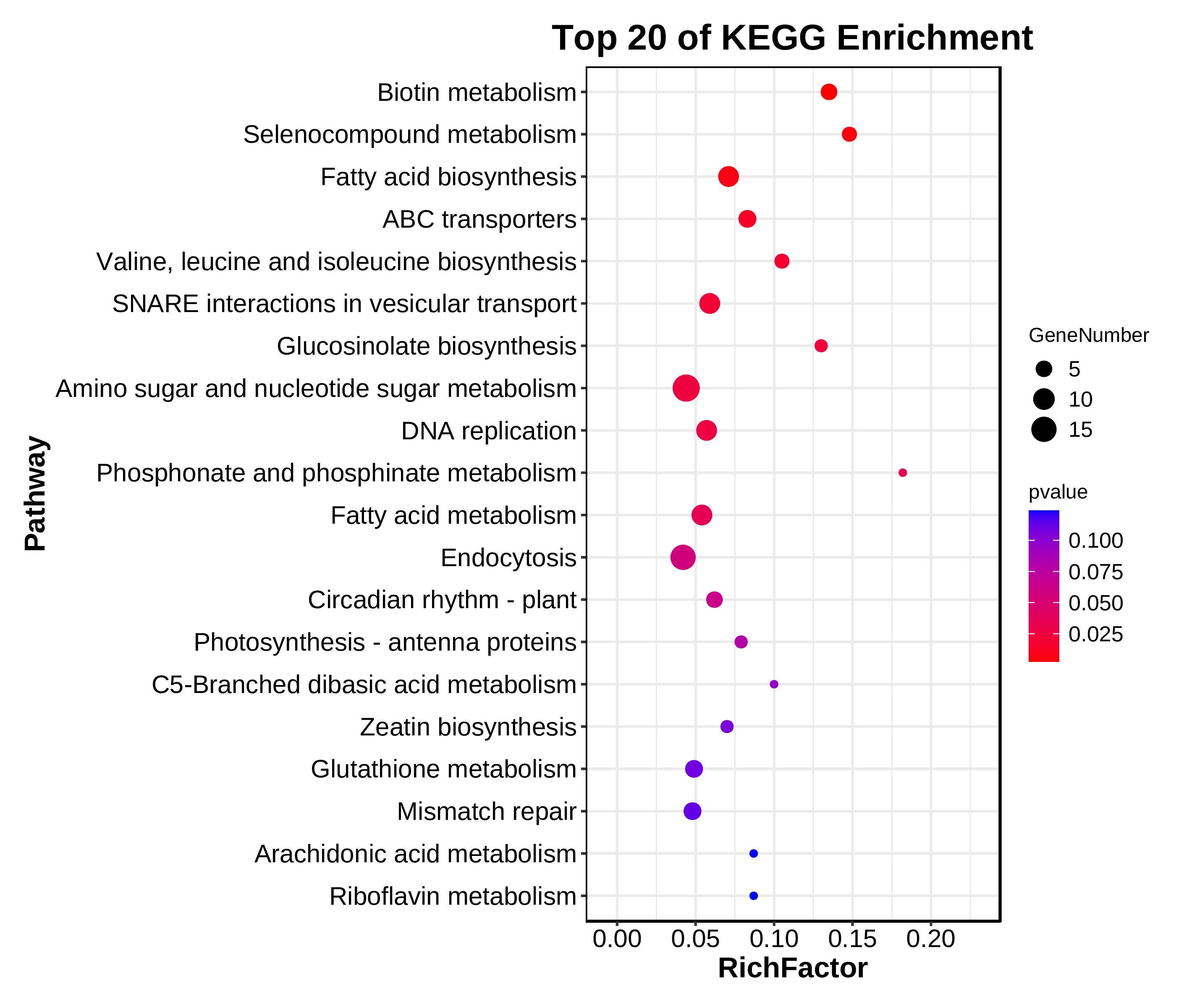


Fig. S13. KEGG enrichment analysis of genes underlying the outliers from RDA. Overrepresented gene ontology terms were identified using a *P*value < 0.05.


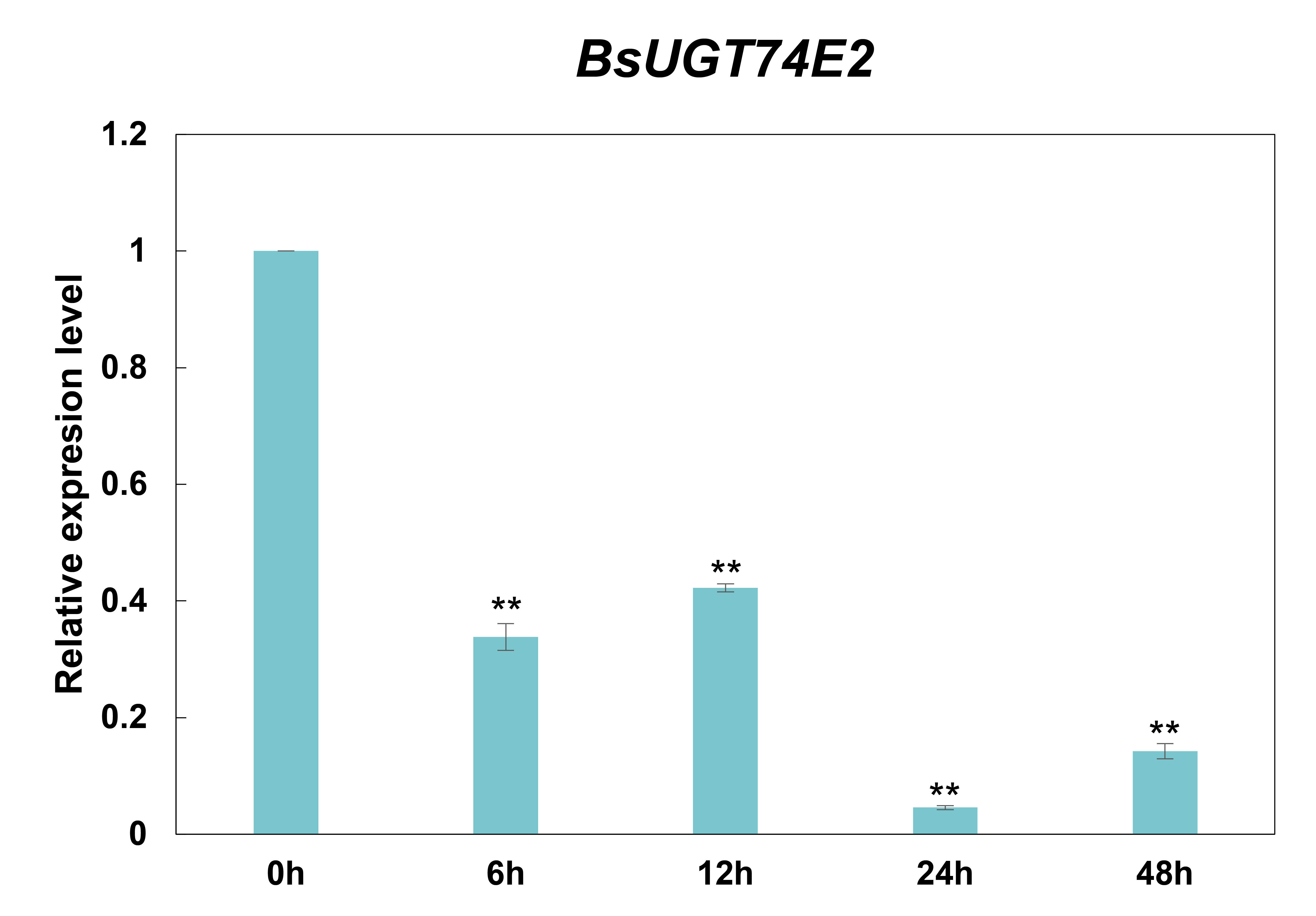


Fig. S14. The mRNA relative expression levels at 0 h, 6 h, 12 h, 24 h and 48 h under cold treatment in *BsUGT74E2* from sterile seedling of *B. scopulosa*. Data are presented as mean ± SE (n = 3). “*” and “**” indicate a significant difference from that of 0 h at *P* ≤ 0.05 and *P* ≤ 0.01, respectively, by student’s *t*-test.





Fig. S15. Subcellular localization of *BsUGT74E2* (*Bsco_038285*) protein in tobacco epidermal cells. Control: 1301 empty vector transformed in tobacco epidermal cells. Bar = 29.8 µm.


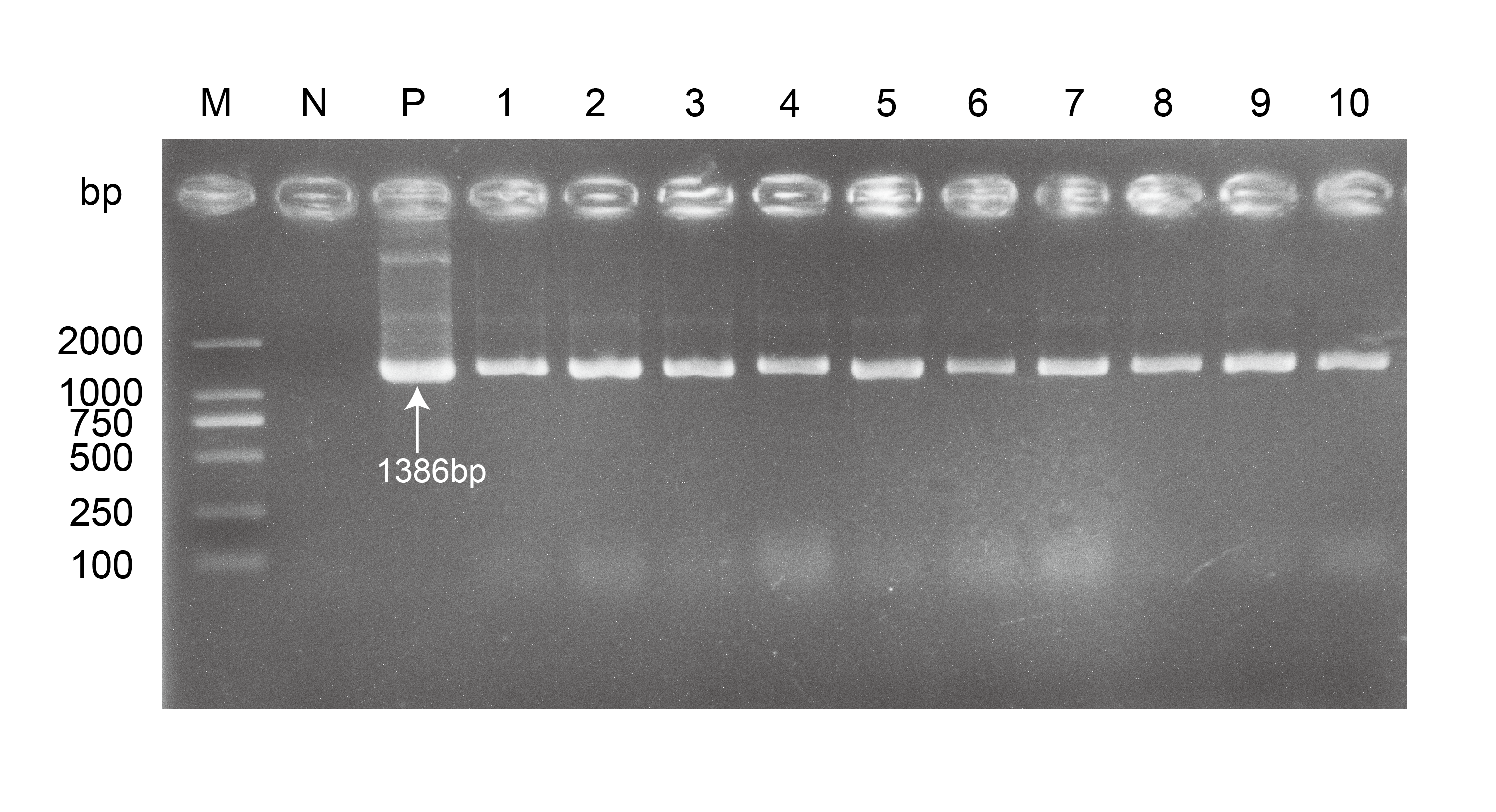


Fig. S16. Identification of transgenic *Arabidopsis* positive seedlings by PCR. 1–10: L1, L3, L7, L9, L11, L12, L19, L20, L24, L28 transgenic *Arabidopsis*; M: D_2000_ marker; N: negative control (WT-type); P: positive control (pCAMBIA1301::*35S-BsUGT74E2-GFP*).


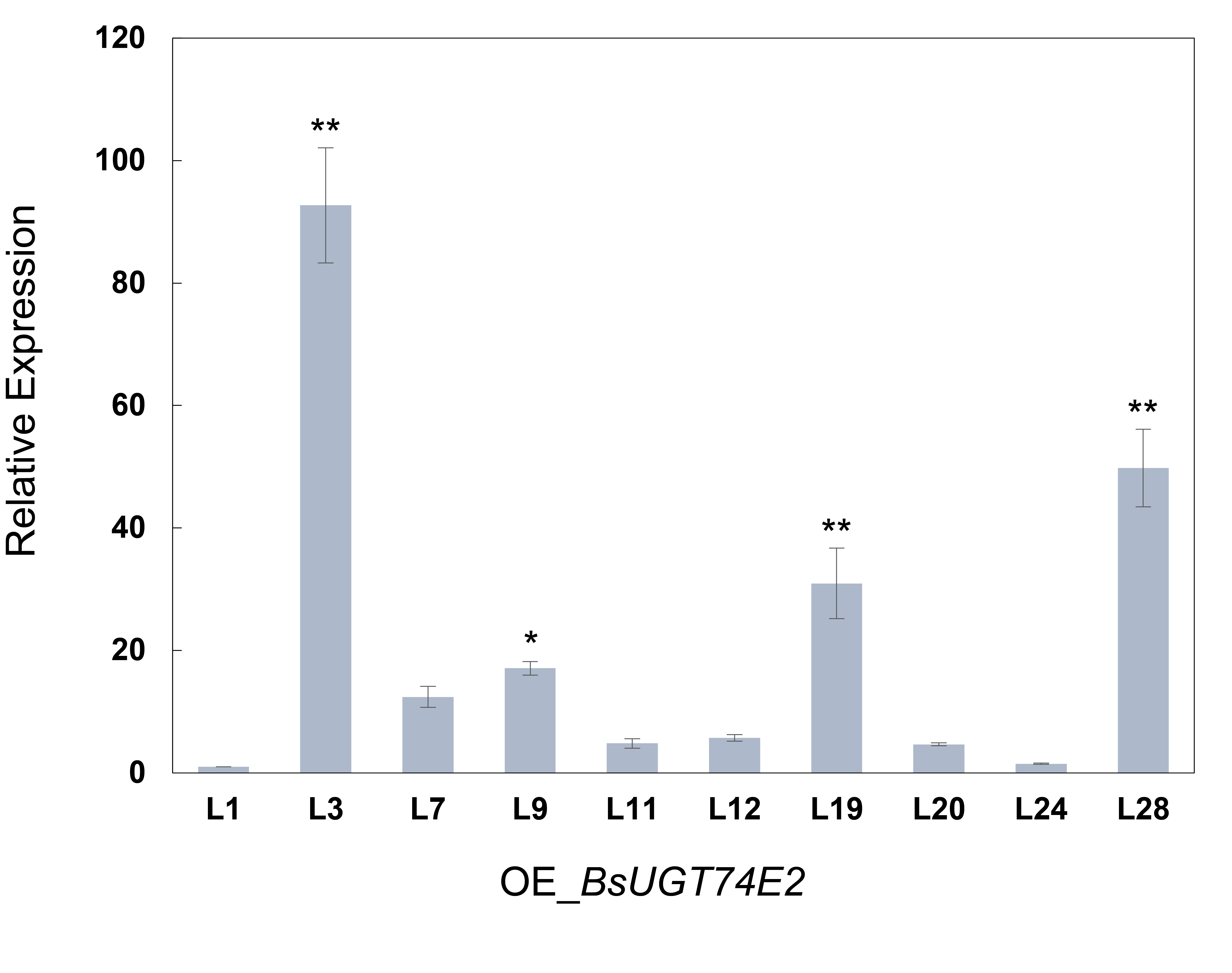


Fig. S17. The relative expression of *BsUGT74E2* in transgenic Arabidopsis thaliana strain. Data are presented as mean ± SE (n = 3). “*” and “**” indicate a significant difference from that of L1 at *P* ≤ 0.05 and ≤ 0.01, respectively, by student’s *t*-test.


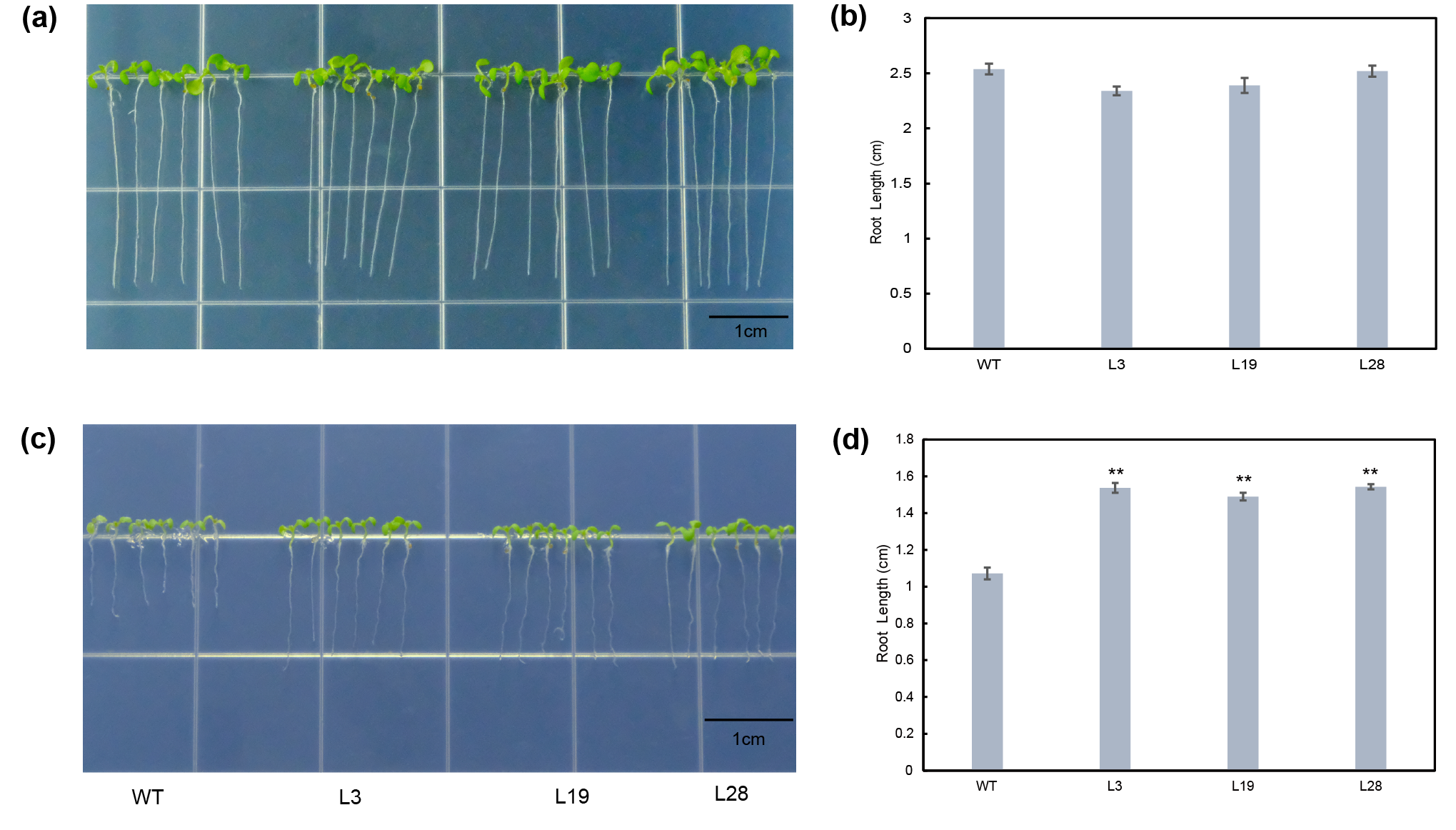


Fig. S18. Effects of *BsUGT74E2* overexpression on *Arabidopsis* seedling root length. (**a, b**) Root length of *BsUGT74E2* overexpressing lines (L3, L19, L28) and WT-type under normal conditions (scale bars = 1 cm). (**c, d**) Root length of WT and *BsUGT74E2* transgenic *Arabidopsis* under cold acclimation (scale bars = 1 cm). Data showed significantly increased root length compared with wild-type plants (**P* ≤ 0.05, ***P* ≤ 0.01 Student’s *t* test). Measurements were made on two independent replicates of 30 seedlings each. Error bars indicate SE.





Fig. S19. Allele frequencies of candidate adaptive SNPs: **(a)** *FAD7*, chr6_12100635, **(b)** *COR413pm2*, chr14_24427933, **(c)** *MYC2*, chr15_22548565, **(d)** *CRF2*, chr14_21366166 associated with BIO3 and BIO4 across the nine populations. Colors mean different alleles. N means missing alleles at leading SNP.
