# Supplementary material for: The chromosome-level genome assembly of an endangered herb Bergenia scopulosa provides insights into local adaptation and genomic vulnerability under climate change
Source: Gigascience. 2024 Nov 28;13:giae091. doi: 10.1093/gigascience/giae091 (PMC11604060; doi:10.1093/gigascience/giae091)
Supplement: giae091_GIGA-D-24-00141_Revision_2 [file giae091_giga-d-24-00141_revision_2.pdf]

GigaScience

The chromosome-level genome assembly of an endangered herb Bergenia scopulosa provides insights into local adaptation and genomic vulnerability under climate change

--Manuscript Draft--

Manuscript Number:	GIGA-D-24-00141R2
Full Title:	The chromosome-level genome assembly of an endangered herb Bergenia scopulosa provides insights into local adaptation and genomic vulnerability under climate change
Article Type:	Research
Funding Information:	National Natural Science Foundation of China (31970359) — Prof. Zhonghu Li<br>Basic Research Project of Shaanxi Academy of Fundamental Science (22JHZ005) — Prof. Zhonghu Li<br>Key Research and Development Projects of Shaanxi Province (2022ZDLSF06–02) — Prof. Zhonghu Li
Abstract:	Background: Global climate change poses severe threats to biodiversity and ecosystem stability. Rapid climate oscillations potentially lead to species geographic range shifts, population declines and even extinctions. The rare and endangered species, being critical components of regional biodiversity, hold the key to understanding local adaptation and evolutionary processes shaping species distributions. Therefore, assessing the evolutionary mechanisms of local adaptation and population vulnerability under climate change is crucial for developing conservation strategies of endangered species.<br>Results: In this study, we assembled a high-quality, chromosome-level genome of the rare and endangered herb Bergenia scopulosa in the Qinling Mountains in East Asia and re-sequenced 37 individual genomes spanning its entire geographic distributional ranges. By integrating population genetics, landscape genomics, and climate datasets, a substantial number of adaptive SNP loci associated with climate variables were identified. The genotype-environment association analysis showed that some cold-tolerant genes have played pivotal roles in cold environmental adaptation of B. scopulosa. These findings are further corroborated through evolutionary analysis of gene family and qPCR validation. Population genomic analysis revealed two distinct genetic lineages in B. scopulosa. The western lineage showed higher genomic vulnerability and more rare cold-tolerance alleles, suggesting its heightened sensitivity to impending climate shifts and should be given priority conservation in the management practices.<br>Conclusions: These findings provide novel insights into local adaptation and genomic vulnerability of B. scopulosa under climate change in the Qinling Mountains in East Asia. Additionally, the study also offers valuable guidance for formulating conservation strategies for the rare and endangered plants.
Corresponding Author:	Zhonghu Li<br>Northwest University<br>Xi’an, Shaanxi Province CHINA
Corresponding Author Secondary Information:	
Corresponding Author's Institution:	Northwest University
Corresponding Author's Secondary Institution:	
First Author:	Yixin Yang
First Author Secondary Information:	
Order of Authors:	Yixin Yang<br>Meng Wang

Powered by Editorial Manager® and ProduXion Manager® from Aries Systems Corporation

	Xuanye Wu
	Yani Zhou
	Jie Qiu
	Xia Cai
	Zhonghu Li
**Order of Authors Secondary Information:**	
**Response to Reviewers:**	Reviewer reports:<br>Reviewer #1: The author assembled a high-quality, chromosome-level genome of the rare and endangered herb Bergenia scopulosa and re-sequenced genomes of 37 individuals that covered the whole geographic distributions of species. By integrating population genetics, landscape genomics, and climate datasets, a substantial number of adaptive SNP loci associated with climate variables were identified. Population genomic analysis revealed two genetic lineages in B. scopulosa. The western lineage was found to exhibit higher genomic vulnerability and greater abundance of rare cold-tolerance alleles. However, additional modification may be needed to improve the manuscript as listed below before acceptance.<br><br>Q: 1. Page 10 line 178, the description "B. scopulosa has undergone two distinct WGD events" required more data support, such as the syntenic depths analysis between B. scopulosa and grape.<br>A: Thank you very much for your thorough review of our manuscript and for the valuable suggestion regarding the need for more data support for the statement "B. scopulosa has undergone two distinct WGD events." We have taken your advice seriously and have conducted additional analyses to strengthen our conclusion.<br>In response to your recommendation, we have included an analysis of the distribution of the four-fold synonymous third-codon transversion rate (4DTv) for paralogous genes within the genomes of B. scopulosa, Tiarella polyphylla, and Vitis vinifera, as well as orthologous genes between B. scopulosa and V. vinifera. These results are presented in Supplementary Fig. S5, which provides further evidence for the two distinct WGD events experienced by B. scopulosa.<br>Specifically, Supplementary Fig S5(a) shows the 4DTv distribution, highlighting the gamma WGD event that is shared by B. scopulosa, V. vinifera, and T. polyphylla, as indicated by the red arrow. These peaks in the 4DTv distribution serves as a clear marker of the WGD event and aligns with our hypothesis. Moreover, Supplementary Fig S5(b) presents the syntenic depths between the genomes of B. scopulosa and V. vinifera. The patterns of syntenic depth and distribution provide additional support for the occurrence of two distinct WGD events in B. scopulosa.<br>We believe that these additional analyses and results presented in Supplementary Fig. S5 significantly strengthen our conclusion regarding the WGD history of B. scopulosa. We are grateful for your insightful comments and suggestions, which have helped us to improve the quality and rigor of our work.<br>Thank you again for your time and effort in reviewing our manuscript. We look forward to any further feedback you may have as we continue to refine and improve our work.<br><br>Q: 2. Page 11 line 201, the description "the expansion and contraction of these genes enhance the adaptability of B. scopulosa in complex environments" is just one possibility in the absence of experimental validation. When referring to different species, these expanded and contracted genes will exhibit significant differences.<br>A: Thank you for your thoughtful comments and suggestions regarding our manuscript. You are absolutely correct in pointing out that the statement "the expansion and contraction of these genes enhance the adaptability of B. scopulosa in complex environments" is indeed a hypothesis that requires further experimental validation. In the absence of comprehensive experimental data for all expanded and contracted genes, such a conclusion can only be made tentatively.<br>To address your concerns, we have revised the relevant section of our manuscript to reflect a more cautious and inferential tone. Specifically, we have emphasized that the observed gene expansions and contractions are intriguing patterns that suggest potential roles in adaptability.<br>Moreover, we would like to highlight that we have taken initial steps towards validating the function of some of these genes. In particular, we have conducted transgenic experiments focusing on an expanded gene, BsUGT74E2 (Bsco_038285), which

Powered by Editorial Manager® and ProduXion Manager® from Aries Systems Corporation

	shows homology to the Arabidopsis UGT74E2. Our preliminary results suggest a role for this gene in cold tolerance, providing a promising starting point for future investigations.<br>We fully agree that the functional validation of these genes is crucial for a comprehensive understanding of their roles in B. scopulosa's adaptability. As such, we have included a discussion of our ongoing and future plans to conduct more extensive experimental validation of the expanded and contracted gene sets. We are committed to pursuing this research direction and hope to contribute to a deeper understanding of the genetic basis of adaptability in this and other species.<br>Thank you again for your valuable feedback. We appreciate your time and effort in reviewing our work, and we look forward to incorporating your suggestions into our future research endeavors.
**Additional Information:**	
**Question**	**Response**
Are you submitting this manuscript to a special series or article collection?	No
**Experimental design and statistics**<br><br>Full details of the experimental design and statistical methods used should be given in the Methods section, as detailed in our Minimum Standards Reporting Checklist. Information essential to interpreting the data presented should be made available in the figure legends.<br><br>Have you included all the information requested in your manuscript?	Yes
**Resources**<br><br>A description of all resources used, including antibodies, cell lines, animals and software tools, with enough information to allow them to be uniquely identified, should be included in the Methods section. Authors are strongly encouraged to cite Research Resource Identifiers (RRIDs) for antibodies, model organisms and tools, where possible.<br><br>Have you included the information requested as detailed in our Minimum Standards Reporting Checklist?	Yes
**Availability of data and materials**	No

Powered by Editorial Manager® and ProduXion Manager® from Aries Systems Corporation

All datasets and code on which the conclusions of the paper rely must be either included in your submission or deposited in publicly available repositories (where available and ethically appropriate), referencing such data using a unique identifier in the references and in the "Availability of Data and Materials" section of your manuscript.<br><br>Have you have met the above requirement as detailed in our Minimum Standards Reporting Checklist?	
If not, please give reasons for any omissions below.<br><br>as follow-up to "**Availability of data and materials**<br><br>All datasets and code on which the conclusions of the paper rely must be either included in your submission or deposited in publicly available repositories (where available and ethically appropriate), referencing such data using a unique identifier in the references and in the "Availability of Data and Materials" section of your manuscript.<br><br>Have you have met the above requirement as detailed in our Minimum Standards Reporting Checklist?<br><br>"	Dear Editor/Reviewer,<br>Thank you for your inquiry regarding the availability of datasets used in our paper. We fully appreciate the importance of transparency and reproducibility in research, and we are committed to sharing our data and materials with the research community.<br><br>Currently, we are preparing the necessary datasets for public deposition in suitable repositories. However, given the complexity and volume of the data involved, we have decided to complete this process after the acceptance of our manuscript. This decision is aimed at ensuring the highest quality and integrity of the data deposition, as well as aligning with our institutional policies and ethical considerations.<br><br>We assure you that once our manuscript is accepted, we will promptly deposit the datasets in publicly available repositories and provide unique identifiers for referencing them in the "Data Availability" section of our manuscript. We will also ensure that the deposition process adheres to all relevant ethical and legal requirements.<br><br>We appreciate your understanding and support in this matter. If you have any further questions or concerns, please feel free to contact us at your earliest convenience.<br>Thank you for considering our submission.

Powered by Editorial Manager® and ProduXion Manager® from Aries Systems Corporation

Manuscript
Click here to access/download;Manuscript;Bergenia_scopulosa_giga-final-

The chromosome-level genome assembly of an endangered herb *Bergenia scopulosa* provides insights into local adaptation and genomic vulnerability under climate change

Yi-Xin Yang [1, 2, †], Meng Wang [1, †], Xuan-Ye Wu [1], Ya-Ni Zhou [1], Jie Qiu [1], Xia Cai [1, *], Zhong-Hu Li [1, *]

[1] Key Laboratory of Resource Biology and Biotechnology in Western China, Ministry of Education, Provincial Key Laboratory of Biotechnology, College of Life Sciences, Northwest University, Xi'an 710069, China

[2] Medical Experiment Center, Shaanxi University of Chinese Medicine, Xianyang 712046, China

[*] For correspondence (e-mail lizhonghu@nwu.edu.cn, caix@nwu.edu.cn)

[†] These authors contributed equally to this article.

Yixin Yang [0009-0001-3619-0892]; Meng Wang [0000-0002-5374-8552]; Xuanye Wu [0009-0009-5902-4086]; Yani Zhou [0009-0007-6811-1064]; Jie Qiu [0009-0002-5734-1183]; Xia Cai [0000-0002-6958-1937]; Zhonghu Li [0000-0001-6763-5586]

Abstract

Background: Global climate change poses severe threats to biodiversity and ecosystem stability. Rapid climate oscillations potentially lead to species geographic range shifts, population declines and even extinctions. The rare and endangered species, being critical components of regional biodiversity, hold the key to understanding local adaptation and evolutionary processes shaping species distributions. Therefore, assessing the evolutionary mechanisms of local adaptation and population vulnerability under climate change is crucial for developing conservation strategies of endangered species.

Results: In this study, we assembled a high-quality, chromosome-level genome of the rare and endangered herb *Bergenia scopulosa* in the Qinling Mountains in East Asia and re-sequenced 37 individual genomes spanning its entire geographic distributional ranges. By integrating population genetics, landscape genomics, and climate datasets, a substantial number of adaptive SNP loci associated with climate variables were identified. The genotype-environment association analysis showed that some cold-tolerant genes have played pivotal roles in cold environmental adaptation of *B. scopulosa*. These findings are further corroborated through evolutionary analysis of gene family and qPCR validation. Population genomic analysis revealed two distinct genetic lineages in *B. scopulosa*. The western lineage showed higher genomic vulnerability and more rare cold-tolerance alleles, suggesting its heightened sensitivity to impending climate shifts and should be given priority conservation in the management practices.

Conclusions: These findings provide novel insights into local adaptation and genomic vulnerability of *B. scopulosa* under climate change in the Qinling Mountains in East Asia. Additionally, the study also offers valuable guidance for formulating conservation strategies for the rare and endangered plants.

Keywords: *Bergenia scopulosa*, genome assembly, local adaptation, genomic vulnerability, conservation.

Introduction

Biodiversity is the material foundation for the survival of all life on Earth. It is a crucial guarantee for maintaining good operation of ecosystems and serves as a source of materials for human life and production, which closely related to human survival and development [1]. However, since the onset of the Industrial Revolution in the 18th century, and particularly during the Anthropocene era, human activities have an ever-increasing impact on the natural world. The ensuing global climate fluctuations have led to the fragmentation of habitats and a decline in the population of most organisms. One of the consequences is a decrease in gene flow and/or genetic exchange between populations, which leads to a reduction in the sharing of adaptive alleles. In extreme cases, this has led to the localized extinction of certain species, which poses a significant threat to both biodiversity and the stability of ecosystems [2-4]. When the rate of climate change surpasses the species' ability to adapt its own environments, it becomes challenging for most plants to adapt to rapidly shifting climates through migration or dispersion [5-7]. Therefore, it is particularly important to evaluate how plant species adapt to complex and ever-changing environments and predict their response mechanisms to future climate changes.

In 2018, Bay et al. proposed the concept of genomic vulnerability as a genotype-environment relationship modeled on contemporary population data to predict the mismatch between current and future genetic variations in the genome of species under changing climate conditions [8]. The concept aims to pinpoint the most vulnerable species to the effects of climate change. A lower degree of match indicates a

population's lesser ability to adapt quickly to future climate change. Thus, genomic vulnerability can be used as an indicator to assess the decline in population size and adaptive capacity. It aids in comprehending and predicting the dynamic changes in population sizes, and has garnered growing interest among researchers [9-11]. However, previous studies have frequently emphasized the ecological adaptability of species' distribution ranges under various climatic scenarios. These analyses primarily rely on species distribution data and environmental variables, neglecting the influence of genetic factors on biological adaptability [12-15]. In recent years, the integration of high-throughput genetic data with environmental factors through landscape genomics analysis methods has gained increasing interest. The evaluation of endangered populations' response capacity to climate change through this approach has become a prominent and widely discussed topic [16-19]. Traditional methods for obtaining adaptive phenotypic data involve "common garden experiments" or "reciprocal transplant experiments". Subsequently, genetic and phenotypic data are correlated through methods such as whole-genome association studies (GWAS) or quantitative trait locus (QTL) mapping, providing insights into the intricate relationship between genotype and phenotype. However, these techniques are not practical for studying non-model species in their natural habitats due to long experimental cycles and the challenges of obtaining adaptive phenotypic traits [5]. As next-generation sequencing (NGS) and whole-genome sequencing (WGS) technologies continue to advance rapidly, the availability of genetic markers for analysis has gradually increased, enabling more comprehensive and accurate genetic studies. Leveraging landscape genomics

approaches, genome-wide scans can now be conducted to identify loci associated with adaptive evolution. By linking these genetic signals with environmental data, genetic loci involved in climate adaptation can be precisely screened out, genetic offset measured, genetic variation integrated with spatial models, and the molecular mechanisms behind local adaptation revealed through genotype-environment associations (GEA) by researchers. This approach offers insights into the genetic basis of species' adaptability to their environmental and addresses the question of how much genetic variation is necessary for populations to cope with environmental changes. Moreover, it aids in determining priority conservation efforts for vulnerable populations and holds significant scientific value in managing and formulating conservation strategies for species facing threats under changing climatic conditions. The integration of landscape genomics with advanced sequencing technologies offers immense potential in understanding the intricate relationship between genetics, environment, and adaptation. By applying this knowledge, informed decisions can be made to protect and conserve species in the face of ongoing climate change [9, 20-24].

Researching the genomic vulnerability of species typically involves conducting whole genome sequencing or resequencing of all individuals within a population to obtain an extensive set of single nucleotide polymorphism (SNPs) markers. By investigating nucleotide variation sites spanning the entire genome, the genetic variation level of the population is comprehensively assessed. Subsequently, association analysis with environmental data is performed to identify specific gene sites under selection and evaluate genetic offset. This comprehensive approach enables the

assessment of genomic vulnerability, offering insights into populations that are particularly susceptible to the effects of climate change [11, 19, 25, 26]. *Bergenia scopulosa* T. P. Wang (NCBI:txid1397840) is an endangered perennial herb belonging to the Saxifragaceae family. The dried rhizomes of *B. scopulosa* have been used as traditional medicine in China, particularly in the Qinling area in East Asia, where it is known as Pan Long Qi [27]. This species is sporadically distributed in the damp undergrowth of forests or in the crevices of cliff faces, exhibiting strong cold resistance. In recent years, the majority of studies have primarily concentrated on the genomic vulnerability of woody plants, while herbaceous plants have received limited attention. Herbaceous plants possess a shorter generation cycle and are highly susceptible to the effects of climate change. Therefore, conducting research on the population history and local adaptation of herbaceous species with relatively narrow habitats can comprehensively understand the interaction between geographical and environmental heterogeneity, and can also address the vulnerability of small and medium-sized populations in biodiversity hotspots, which is helpful for the protection and management of threatened species. In this study, our focus was on the rare and endangered medicinal plant *B. scopulosa*, which is endemic to the Qinling Mountains region in China. Through preliminary research and extensive population sampling, we successfully sequenced the genome of *B. scopulosa* at the chromosome level. Subsequently, we conducted resequencing analysis on 37 individuals from nine populations covered its natural geographic distributions. Using a combination of population genetics, landscape genomics, and environmental modelling methods, we

firstly investigated the genetic structure and population demographic history of this species. Additionally, we assessed the capability of different populations to adapt to climate change based on environmental data. Furthermore, we explored the molecular mechanisms underlying adaptation to diverse climatic conditions. Finally, we evaluated the genomic vulnerability of various geographic populations of *B. scopulosa* in the face of rapid climate change expected in the future.

Results

Genome sequencing, assembly, and annotation

A total of 26,925,281,224 k-mers of length 17 were generated and the peak depth was 36. The genome size of *B. scopulosa* was estimated to be 737.09 Mb with a heterozygosity of 0.87% and a repeat sequence ratio of 63.04% (Supplementary Table S1; Supplementary Fig. S1). After trimming and quality control, 94.92 Gb (~129× coverage) of short reads, 22.73 Gb (~31× coverage) of long reads, and 113.30 Gb (~154× coverage) raw Hi-C data were generated (Supplementary Table S2). The final assembly captured 733.32 Mb of the genome sequence, with contig N50 of 20.84 Mb and a chromosome-size scaffold N50 of 37.96 Mb (Supplementary Table S3). Over 92.86% of the contig sequences (~680.95 Mb) were successfully anchored to 17 pseudo-chromosomes (Fig. 1; Supplementary Table S4; Supplementary Fig. S2). We evaluated the completeness of the *B. scopulosa* genome using Benchmarking Universal Single-Copy Orthologs (BUSCOs). Evaluation against three databases indicated that the genome is between 98.7% to 99.5% complete, highlighting the high quality of our assembled genome (Table 1; Supplementary Table S5; Supplementary Fig. S3).

We predicted 45,222 protein-coding genes in the *B. scopulosa* genome using ab initio, homology-based and transcriptome-based gene prediction methods. To initiate a functional exploration of the *B. scopulosa* genome, we submitted all gene models to the NR, Swissport, EggNOG, COG, TrEMBL, Gene Ontology (GO), and Kyoto Encyclopedia of Genes and Genomes (KEGG) databases. Of all genes, 42,119 (92.18%) were annotated in these databases (Supplementary Table S6). Concurrently, our investigation led to the identification of a set of non-coding RNAs (Supplementary Table S7). Further analysis revealed that 67.36% of the assembled genome is composed of repetitive sequences, with a predominant composition of 33.58% retroelements and 0.96% DNA transposons. Long terminal repeat (LTR) retrotransposons constituted a significant portion, encompassing 25.19% of the genome, with Ty1/copia (8.84%) and gypsy/DIRS1 (10.74%) being notable contributors (Supplementary Table S8; Supplementary Fig. S4).

Evolution and phylogeny of the *B. scopulosa* genome

A comparative genomic analysis of *B. scopulosa* was performed with nine other plant genomes. These ten species shared 31,623 gene families (orthogroups), with 691 gene families comprising 5658 species-specific genes unique to *B. scopulosa*. Phylogenetic inference based on 299 single-copy orthologous genes showed that *B. scopulosa* diverged approximately 108.6 million years ago (Mya) from *Kalanchoe fedtschenkoi* (Fig. 2a-b). The Ks value of *B. scopulosa*- *K. fedtschenkoi* (BsKf) was 1.5403. Using the formula (T = Ks/2r), we determined that the recent whole-genome duplication (WGD) event in *B. scopulosa* (Ks = 0.2531) occurred approximately 17.85 Mya. In the

vicinity of this Ks value, we discerned pronounced peaks indicative of recent whole-genome duplication (WGD) events in *B. scopulosa*. Notably, our analysis revealed that both *Tiarella polyphylla* and *B. scopulosa*, members of the Saxifragaceae family, exhibit a distinct ancient peak at approximately Ks = 1.2. After adding *Vitis vinifera* for analysis, we found that this peak appeared before the divergence peak between *B. scopulosa* and *V. vinifera*, suggesting that *B. scopulosa* experienced the same gamma WGD event as *V. vinifera* and *T. polyphyllac*. Moreover, the microsynteny patterns further support two WGDs on the lineages leading to *B. scopulosa*. Specifically, the microsynteny pattern reflects a 4:1 gene copy ratio between the *B. scopulosa* genome and the *V. vinifera* genome (Fig. 2c; Supplementary Fig. S5). Furthermore, gene family evolution analysis revealed that 5,495 gene families had expanded, constituting 46.20% of all gene families, while 950 gene families had contracted (7.99% of the total). Notably, 329 expanded and 5 contracted gene families exhibited statistical significance ($P < 0.05$) in *B. scopulosa*.

To understand their biological functions, we conducted KEGG and GO analyses. GO analysis highlighted that the significantly expanded gene families were enriched in processes such as response to salicylic acid (GO:0009751), regulation of flavonoid biosynthetic process (GO:0009962), response to gibberellin (GO:0009739), regulation of seed development (GO:0080050), regulation of translation in response to stress (GO:0043555), response to jasmonic acid (GO:0009753), response to light intensity (GO:0009642) (Supplementary Table S9). In KEGG analysis, most of the expanded genes were clustered in oxidative phosphorylation (ko00190), photosynthesis

(ko00195), flavonoid biosynthesis (ko00941), biosynthesis of various plant secondary metabolites (ko00999), plant hormone signal transduction (ko04075) (Supplementary Table S10). The contracted gene families were associated with GO terms linked to hydroxyjasmonate sulfotransferase activity, monocarboxylic acid metabolic process, organic acid metabolic process, response to desiccation, small molecule metabolic process, etc. (Supplementary Table S11). Furthermore, KEGG pathways analysis for the contracted genes indicated their involvement in ubiquitin mediated proteolysis, phenylalanine, tyrosine and tryptophan biosynthesis and biosynthesis of various plant secondary metabolites (Supplementary Table S12). We posited that the expansion and contraction of these genes might enhance the adaptability of *B. scopulosa* in complex environments, allowing it to better cope with stress and regulate its growth, ensuring survival under abiotic stress conditions [28-32].

Population structure, genetic diversity and demographic history

To explore genetic variation in *B. scopulosa*, we re-sequenced 37 individuals from nine wild populations across the entire range of its distribution in the Qinling Mountain areas with an average depth of ~25× (Supplementary Fig. S6; Supplementary Table S13). Based on our high-quality genome as a reference, 13,044,067 SNPs was obtained. In population genetic analysis, although the cross-validation (CV) error value is minimized at $K = 3$ (cv_error = 0.5457) (Fig. 3b), the nine populations of *B. scopulosa* exhibit a clear division into two distinct lineages (West and East) when $K = 2$ (cv_error = 0.5466). These lineages correspond to the geographical locations on the east and west sides of the Qinling Mountains (Fig. 3a,c) in East Asia, which is further reinforced by

the findings obtained from the principal component analysis (PCA) (Fig. 3d) and non-rooted branching maximum-likelihood (ML) phylogenetic tree (Fig. 3e). Notably, when $K = 3$, the eastern lineage underwent further genetic differentiation within, whereas the western lineage remains relatively stable (Fig. 3c). Given this observation, we have decided to select the geographically distinct eastern and western lineages for subsequent in-depth analysis. Analysis of nucleotide diversity (π) revealed that the eastern lineage exhibited higher genetic diversity compared to the western lineage (Supplementary Fig. S7). Notably, regions of the genome with elevated differentiation and reduced diversity were pinpointed between the eastern and western lineages (Supplementary Fig. S8). Further investigation into the functional analysis of genes within these regions revealed a significant correlation with plant stress resistance (Supplementary Fig. S9; Supplementary Table S14). This finding underscores the potential role of these genomic regions and associated genes in shaping the adaptive responses of *B. scopulosa* populations to their environment [33-39].

To investigate the demographic history of this species, we used the Pairwise Sequential Markovian Coalescent (PSMC) analysis over a substantial timescale. It unveiled a decline in the effective population size, plummeting from approximately 3.9×10^4 to 0.75×10^4 individuals around 0.19 Mya (Fig. 3f). For a more precise understanding of recent population changes in the two lineages, the SMC++ was used for population statistics modelling. Our results showed a rapid population growth in two lineages over the last 25-200 years and suggested their divergence occurred ~130,000 years ago (Fig. 3g).

Genome-environment association revealed genetic loci associated with local adaptation

Two genome-environment association methods: latent factor mixed models (LFMM) and redundancy analysis (RDA) were utilized to delve into the genetic mechanisms underlying local adaptation of *B. scopulosa*. Leveraging high-quality variant SNPs identified in the earlier genetic analysis, we conducted machine learning regression analysis using the “gradient Forest” package in R to execute gradient forest analysis. After considering variable importance and correlations, we identified five climate variables most strongly linked to genetic variation (i.e., BIO3: Isothermality, BIO4: Temperature Seasonality, BIO15: Precipitation Seasonality, BIO18: Precipitation of Warmest Quarter, BIO19: Precipitation of Coldest Quarter) (Supplementary Fig. S10). Subsequently, we utilized LFMM to examine the correlation between predictive variables and allele frequencies. A total of 40,529 SNPs colocalized with 1,702 genes were identified across five environmental factors. The terms with the significant enrichment levels were found to be: Plant hormone signal transduction (ko04075, $P = 9.06 \times 10^{-3}$), Circadian rhythm - plant (ko04712, $P = 4.25 \times 10^{-2}$) (Supplementary Fig. S11). Within the realm of plant hormone signal transduction, our investigation has unveiled several key genes that orchestrate the plant's adaptive response to adverse conditions. Specifically, *BsSAUR14* (*Bsco_041147*) was colocalized with one SNP chr14_25686183 ($P = 6.19 \times 10^{-4}$) which was identified using LFMM for BIO4, hinting at its role in stress tolerance [40]. Furthermore, delving into the circadian rhythm - plant pathway revealed a profound connection between diurnal regulation and plant

resilience, with notable genes including *BsCRY1* (*Bsco_034373*, chr4_14735339, $P = 1.79 \times 10^{-5}$) and *BsPhyA* (*Bsco_006033*, chr6_8489173, $P = 4.69 \times 10^{-4}$), both implicated in the plant's ability to withstand stressful environments [41, 42]. Additionally, we conducted a multivariate regression analysis combined with RDA on the allele frequency data of *B. scopulosa* with five climate variables selected, and found that environmental factors can explain 44.11% of the genomic variation. RDA1 and RDA2 accounted for 24.98% and 7.07% of the genetic variation (Supplementary Fig. S12) respectively, indicating that environmental factors play a significant role in the genetic diversity and local adaptation of *B. scopulosa*. Specifically, 11,163 outlier SNPs were detected in six RDAs and 1,440 adaptive genes were identified in the 100 kb interval around these outliers. Remarkably, our study also identified a cohort of genes implicated in plant stress tolerance, despite their failure to reach statistical significance within the KEGG pathway analysis framework. Among these genes, notable mentions include *BsHSP70* (*Bsco_031051*, located at chr16_15031245, with an RDA score of −0.3340 in RDA5) associated with the Endocytosis pathway (ko04144, $P = 0.057$), and *BsPhyA* (*Bsco_006033*, located at chr6_8493265, with an RDA score of 0.2235 in RDA5) in Circadian rhythm – plant pathway (ko04712, $P = 0.064$), which was also selected by the LFMM method, highlighting its potential significance [43] (Supplementary Fig. S13). Collectively, these genes underscored the pivotal roles they play in orchestrating the adaptability and resilience of plants, particularly those belonging to rare and endangered species capable of enduring cold conditions. Their ability to thrive in harsh environments is intimately tied to the intricate regulation of

these crucial genetic factors. In summary, our analysis has pinpointed 931 core SNPs, represented by overlapping markers, that are consistently identified by both the LFMM and RDA methods. Notably, a discrepancy of 272 genes emerges between the two approaches, highlighting the significance of employing diverse algorithms to achieve a more comprehensive and robust identification of adaptive genes.

Freezing - tolerance - related genes involved in the local adaptation of *B. scopulosa*

In this study, cold-related adaptive genes in the 100 kb interval around outlier SNPs were successfully identified using both LFMM and RDA algorithms (Supplementary Table S15), including *GI* (*Bsco_012979*), *CIPK21* (*Bsco_033313*), *COR413pm2* (*Bsco_041002*), *MYC2* (*Bsco_038291*), *MED2* (*Bsco_041019*), *CRF2* (*Bsco_036510*), *BRS1* (*Bsco_031057*) and *FAD7* (*Bsco_006378*). These genes are known to play crucial roles in cold tolerance and adaptation [44-48], highlighting the significance in *B. scopulosa*'s ability to thrive under challenging cold conditions. To validate the expression of these genes, we subjected sterile seedlings of *B. scopulosa* to cold (4 °C) treatment and conducted qPCR analysis at five time periods (0, 6, 12, 24, and 48 hours). Compared to the untreated group (0 h), significant differences in gene expression were observed (Fig. 4a), indicating their involvement in the response to cold stress. By examining their expression patterns at different time points, we have gained a deeper understanding of the regulatory mechanisms underlying cold adaptation in *B. scopulosa*. We further conducted an analysis of the expression patterns of these eight genes across various tissues and found that the *FAD7* (Omega-3 fatty acid desaturase 7, *Bsco_006378*) gene was particularly prominent in all tissues (Fig. 4b). Consequently, we delved into

the evolutionary relationships within the *FAD* gene family of *B. scopulosa*. To this end, a phylogenetic tree was constructed using 27 predicted FAD proteins from *B. scopulosa* and 143 FAD proteins from *Arabidopsis*, wheat, rice, and soybean. The results indicated that the 27 FAD members of *B. scopulosa* can be categorized into 6 distinct groups, including DES/SLD, FAB2, FAD2, FAD4, FAD3/FAD7/FAD8, and FAD6 (Fig. 4c). Notably, *BsFAD7* (*Bsco_006378*) exhibited the highest expression across almost all tissues within the FAD3/FAD7/FAD8 group (Fig. 4d), strongly suggesting its significant role in cold resistance.

Genomic offset prediction for future climate change

To elucidate the population-level vulnerability to climate change, we employed a visualization technique known as "genetic offset" that incorporates different climate scenarios within geographic space. By mapping the genetic offset, we can identify areas where certain populations exhibit greater or lesser resilience to changing climatic conditions. This visualization method helps us understand the extent of vulnerability and informs targeted interventions and mitigation strategies. The GF modeling analysis revealed that the western lineage of *B. scopulosa* in the Taibai Mountains, is projected to have high genetic offset values in response to future climate scenarios (ssp_126 and ssp_585) during the periods 2061-2080. It suggests that the west lineage is comparatively more susceptible to future environmental changes. Given the rich diversity of genetic resources adaptable to various climatic conditions in the west lineage, prioritizing and enhancing conservation efforts, along with targeted protection measures, for the populations in this region are essential (Fig. 5).

Discussion

Global climate change is indeed a significant concern as it poses threats to biodiversity and ecosystem stability [49-52]. Rare and endangered species are crucial for regional biodiversity, especially when the populations are extremely small and at risk of extinction. These species are prone to genetic drift and mutation loads, which can hinder their ability to adapt local environment and ultimately threaten their survival. And such challenges are closely linked to climate fluctuations [53, 54]. Therefore, comprehending local adaptation mechanisms and genomic vulnerabilities of species in the face of climate change is crucial for crafting conservation policies protecting rare and endangered species. *B. scopulosa*, an endangered herb species in the Qinling Mountains in East Asia, offers valuable medicinal properties, and survives even during severe winters (Fig. 1). This species is an excellent candidate for exploring the genetic mechanisms of local adaptation. However, limited high-quality genome data has hindered the present researches. To address this issue, we utilized advanced sequencing and chromosomal mapping techniques to generate a high-quality chromosome-level genome for this endangered species. Further analysis was conducted on population genomics and landscape genomics. The results of this study will deepen our understanding of genomic evolution and population history of in the *Bergenia* and provide valuable insights for studying the local adaptation mechanisms and conservation strategies. Moreover, our assembled high-quality genomes still contain gaps. In recent years, with the development of multi-platform sequencing technology, an increasing number of telomere-to-telomere (T2T) gap-free genomes have been

assembled. T2T genomes play a crucial fundamental role in understanding the structure of new genes, centromeric regions, whole-genome methylation levels, repetitive sequence variations, transposon movement, centromere evolution, and the identification of quantitative trait loci (QTLs) related to important traits [55-58]. Therefore, the quality of our assembled genomes still needs to be further improved in future research to provide more references for exploring the functional and regulatory mechanisms of the genome in this species.

Natural selection is the primary force behind species evolution and differentiation. It acts on genetic variation within populations, favor traits that enhance an individual's fitness in a given environment. As a result, the same species can diverge in different environments through local adaptation, and thus reflecting their potential for responding to environmental changes. Comparative genomics suggests that the recent whole-genome duplication event in this species occurred approximately 17.85 Mya (Fig. 2c), corresponding to the transition from the middle Miocene to the Mid-Miocene Climate Optimum (MMCO, approximately 16.9–14.7 Mya). This epochal period marked a remarkable transition from a "icehouse" to an "hothouse" climate, during which the MMCO made the climate in most parts of the world warmer, about 5-10 °C higher than the current average temperature. Consequently, organisms inhabiting this transitional era faced unprecedented challenges [59, 60]. Furthermore, another older WGD event of this species occurred approximately 85 Mya, marking a crucial turning point from the typical greenhouse climate phase of the Late Cretaceous to a period of global cooling [61]. During this time, the terrestrial climate underwent significant

fluctuations, exerting considerable influence on plant species. So, we postulated that the whole-genome duplication event during this period provided the species with additional genetic material, enhancing its evolvability and adaptability to challenging environments. Furthermore, analysis of the expansion and contraction of gene families within the GO/KEGG pathways suggests that these genetic alterations have strengthened the species' capacity to respond to stress and regulate its growth effectively. Besides, during our initial field investigation, it was intriguing to discover that the wild *B. scopulosa* populations extends from the west to the Taibai Mountains (the highest peak of the Qinling Mountains) and to the east to Chang'an district of Xi'an city. This remarkable distribution is further highlighted by the Qinling Mountains in East Asia, which divide northern and southern China and host diverse habitats, including many rare and endangered plant species. The Qinling Mountains' barrier effect on water vapor creates distinct climates on its northern and southern slopes. While previous studies concentrated on the central region, recent research reveals a temperature pattern over the past 60 years characterized by lower temperatures in the middle, higher temperatures around the periphery, with a predominance of warmer temperatures in the east and south compared to the west and north. As global warming continues, the western and middle sections of the Qinling Mountains have shown the strongest response to rising temperatures [62]. Additionally, researches have demonstrated that during the onset of contemporary warming periods, the impact of altitude on vegetation's response to climate becomes increasingly pronounced [63, 64]. Our population genetics analysis, based on resequencing data, identified two distinct

geographic groups (West & East) (Fig. 3a-e). This suggests that the population in the Taibai Mountains may be more vulnerable to the effects of climate change. SMC++ analysis backs our hypothesis, revealing that roughly 130,000 years ago, the two lineages started to diverge (Fig. 3g), which coincides with the interglacial stage of the late middle Pleistocene epoch, characterized by a frigid climate and widespread glaciation. Notably, the western lineage showed a steeper decline in effective population size than the eastern lineage (Fig. 3g), indicating its greater vulnerability to climatic shifts.

Low temperature, a critical environmental factor, restricts plant growth and distribution [65]. Plants adapt to cold temperatures through a process called cold acclimation, which involves physiological and biochemical adjustments that enhance their tolerance. However, due to the greenhouse effect, global winter temperatures are rising, leading to more frequent temperature fluctuations. This disrupts the cold acclimation process in plants, potentially resulting in earlier deacclimation and increased risk of winter freeze damage [66-69]. As a species resilient to cold temperatures, we have narrowed our research focus to genes linked to cold tolerance. In our study, we utilized two distinct methods, LFMM and RDA, to identify genes involved in genome-environment interactions. Although previous studies have delved into the strengths and limitations of these methods [70, 71], some studies even have suggested that RDA is more effective in detecting adaptive loci compared to LFMM [71, 72]. While our findings indeed align with this conclusion, highlighting the RDA method's ability to pinpoint adaptive loci, we also observed that both methods were

effective in detecting cold adaptation loci in *B. scopulosa* (Supplementary Table S15). Therefore, we recommend utilizing both LFMM and RDA to obtain a more comprehensive understanding of core loci.

To be more specific, the GIGANTEA (*GI*) gene is a key regulator, not only in cold adaptation [73], but also in controlling the timing of plant flowering. The qPCR results demonstrated a significant upregulation of *GI* gene expression as the duration of cold acclimation increases. Research has shown that the *GI* gene upregulates CO transcription, leading to a faster flowering process [74, 75]. Based on these findings, we proposed the following scenario: as winter approaches and temperatures drop, the *GI* gene of *B. scopulosa* is significantly upregulated, enabling the species to continue flowering during the cold winter and facilitating a faster completion of its life cycle. Additionally, the membrane-bound *FAD* genes play a pivotal role in maintaining normal plant growth under low-temperature stress [76, 77]. In rice, the expression of *OsFAD8* is significantly induced by low temperatures [78], while the overexpression of the *FAD7* gene enhances cold resistance in transgenic tobacco materials [79]. Our qPCR results for the *BsFAD7* gene align with these findings, highlighting its crucial role of this gene in the adaptation of *B. scopulosa* to low-temperature environments. Furthermore, the qPCR analysis of the core gene *Bsco_038285* from the UDP-glycosyltransferase (UGT) gene family revealed a significant reduction in its expression level under cold treatment (Supplementary Fig. S14). This gene shares homology with *UGT74E2* in *Arabidopsis thaliana*, which is a glucosyltransferase enzyme involved in the glycosylation modification of the hormone auxin IBA with important role in maintaining the dynamic

balance of plant hormones [80]. For a deeper exploration of its function (Supplementary Fig. S15), it was integrated into the genome of wild-type *Arabidopsis* and three transgenic lines with high expression levels were chosen for further experimental analysis (Supplementary Fig. S16-17). Remarkably, after two weeks of cold acclimation, the transgenic *Arabidopsis* lines exhibited significantly longer roots compared to the wild type. However, it's noteworthy that under normal conditions (22 °C), there were no significant morphological differences between the WT and *BsUGT74E2*-OEs *Arabidopsis* seedlings (Supplementary Fig. S18). This phenomenon aligns with previous studies [81-83], leading us to speculate that the ectopic expression of this gene in *Arabidopsis* might have a limited impact on regulating IBA homeostasis. When plants encounter stressful conditions, their metabolic resources are redistributed among various physiological pathways, often resulting in stress symptoms such as growth retardation and reduced metabolism. Previous studies have demonstrated that auxin plays a pivotal role in this adaptive response [84]. Upon exposure to cold stress, numerous genes are activated, triggering an increase in various metabolites and protein levels, some of which contribute to a certain degree of cold tolerance. Therefore, we hypothesize that this gene may serve as a crucial component in the cold response mechanism and play a significant role in the adaptive evolution of the species. And future research requires more extensive and profound experiments to further explore its underlying mechanisms. Meanwhile, several studies have indicated that the cold-responsive (*COR*) gene plays a crucial role in enhancing plant cold resistance [85-87]. In our study, we observed a significant increase in the expression level of the *COR* gene

as the duration of cold treatment increased, clearly demonstrating its importance in facilitating the adaptation of plants to frigid environments. Furthermore, to gain a deeper understanding of this topic, we opted to utilize several core genes as exemplary representatives for investigating the intricate distribution patterns of allele frequencies within nine populations (Supplementary Fig. S19). Our comprehensive analysis unveiled that a unique set of rare alleles is predominantly present in the Taibai Mountain populations, belonging to the western lineage, where climatic conditions, characterized by notably lower temperatures, contrast sharply with those of the Guanzhong Area in the eastern lineage. This revelation underscores the pivotal role these rare alleles play within the selected genes in facilitating adaptation to the challenging high-altitude environments of the western region. Additionally, in *FAD7*, the varying homozygous and heterozygous states of these differential alleles between the two lineages may also be a factor contributing to the increased vulnerability of the western lineage. In summary, a series of core cold-adaptation genes collectively shape the local adaptation pattern of this species. However, with the frequent occurrence of extreme climate events, this endangered cold-tolerant species is also facing challenges brought about by climate change. Consequently, safeguarding the rare alleles within more core genes and identifying those related to cold tolerance will provide invaluable genetic resources for the future conservation efforts of this species.

As historical climate fluctuations intertwine with human influence on the environment, global climate changes fragment species habitats, reducing gene flow and adaptive genetic variation, potentially leading to local extinctions, especially in small,

endangered populations [88]. Our research not only aims to clarify the connection between local adaptation mechanisms and environmental factors, but also to utilize genomic adaptive information to predict population vulnerability in the face of climate change. Wild *B. scopulosa*, rich in bergenin and highly valued in traditional medicine, is currently overexploited by local farmers, leading to a significant population decline. In our study, we observed a notable increase in the effective population since last 25 years in eastern lineage, possibly due to the increased awareness of the ecological diversity present in the Qinling Mountains. This heightened awareness has prompted the implementation of government-led ecological conservation plans, strengthening the protection of rare and endangered species in the region. Furthermore, our research has revealed that the population of Taibai Mountain in the western lineage exhibits higher genetic offset (Fig. 5), indicating a heightened vulnerability to future environmental shifts. Climate change poses a global challenge, and the Chinese government has taken a series action to conserve biodiversity, including establishing a network of nature reserves, reinforcing species protection, and restoring habitats. Given the increased vulnerability of the Taibai Mountain populations and their valuable genetic resources adapted to cold climates, it is imperative to prioritize and implement targeted conservation strategies for these populations. For the entire population, the primary objective is to expand the population size while maintaining current numbers. Firstly, it is essential to enhance the protection and monitoring of its native habitat and plants. Establishing conservation sites dedicated to this species and preserving its genetic diversity *in situ*. Additionally, based on our previous seed germination experiments, we

have found that this species exhibits higher germination rates in laboratory conditions compared to the lower rates observed in the wild. Therefore, it is advisable to collect seeds of this species and engage in artificial cultivation for *ex situ* conservation. This will help maintain sufficient genetic diversity and maximize the environmental adaptability of this species. Finally, considering the overwhelming demand for traditional Chinese medicine, it is urgent establish a swift propagation system for this rare and endangered species to ensure its sustainable utilization.

Materials and Methods

Plant materials and genome sequencing

The natural plants of *B. scopulosa* were collected from a single plant in the Qinling Mountains, Shaanxi Province, China (N33°57’42”, E109°3’37”) to ensure genetic diversity and representativeness. Total genomic DNA was obtained using a modified method of SDS-CTAB and sequenced on an Illumina NovaSeq 6000 sequencing system (RRID:SCR_016387) for short-read sequencing and the PacBio SEQUEL2 platform (RRID:SCR_017990, Pacific Biosciences, Menlo Park, CA, USA) for long-read sequencing. Fresh young plant was used to create the Hi-C libraries. For each library, the chromatin was fixed with formaldehyde in the nucleus, and the cross-linked DNA was digested using the restriction enzyme DpnII. Hi-C sequencing libraries were amplified by PCR (12-14 cycles) and sequenced on Illumina NovaSeq 6000 sequencing system (RRID:SCR_016387).

Genome size estimate and assembly

To estimate the genome size of *B. scopulosa*, clean Illumina reads were used to calculate

the 17-K-mer distribution using SOAPdenovo (RRID:SCR_010752) [89]. The estimation of genome size was computed using the formula: G = k-mer_number/k-mer_depth [90]. Long clean reads of PacBio were assembled using Hifiasm v0.15.4-r343 (RRID:SCR_021069) [91]. The primary contigs were filtered and error-corrected with the Nextpolish (RRID:SCR_025232) [92] using Illumina short reads. For Hi-C library preparation, we employed the alignment strategy of HiC-Pro (RRID:SCR_017643) [93] and utilized bowtie2 (RRID:SCR_016368) [94] for alignment. We applied the LACHESIS (RRID:SCR_017644) [95] algorithm with a bottom-up hierarchical clustering method to cluster scaffolds into 17 chromosome groups. After Hi-C-assisted assembly, the sketch contigs/scaffolds were anchored into pseudo-chromosomes. Ultimately, we evaluated the integrity of the *B. scopulosa* genome using the BUSCO v5.1.2 (RRID:SCR_015008) [96].

Genome annotation

Homology-based annotation of the *B. scopulosa* genome was performed using a collection of protein-coding genes from seven plant species: *A. thaliana*, *Beta vulgaris*, *Salvia bowleyana*, *K. fedtschenkoi*, *Oryza sativa*, *Rhodiola crenulate* and *Solanum lycopersicum*. Repeat elements were annotated using RepeatModeler v2.0.1 (RRID:SCR_015027) [97] which primarily utilizes two programs, Recon (RRID:SCR_021170) [98] and RepeatScout (RRID:SCR_014653) [99]. For genome structure annotation, we used the Augustus package (RRID:SCR_008417) [100] within the Braker v2.1.5 (RRID:SCR_018964) [101] for de novo gene prediction. Additionally, the MAKER v3.01.03 pipeline (RRID:SCR_005309) [102] was chosen to predict

protein-coding gene models in *B. scopulosa*. For gene function annotation, Blastp (RRID:SCR_001010) was employed with an E-value threshold of ≤ $1e^{-5}$ to align the annotated genes with eggNOG, GO, COG, and KEGG databases [103-105]. For non-coding RNA annotation, tRNAscan-SE v2.0 (RRID:SCR_008637) [106] was used to identify tRNA sequences, while Blastn (RRID:SCR_001598) was employed to retrieve specific rRNA sequences. INFERNAL v1.1.3 (RRID:SCR_011809) [107] based on the Rfam (RRID:SCR_007891) [108] covariance models was used to predict miRNA and snRNA sequences in the genome.

Comparative genomic and evolutionary analyses

Nine high-quality genomic data were selected from *A. thaliana*, *O. sativa*, *Amborella trichopoda*, *K. fedtschenkoi*, *B. vulgari*, *Paeonia ostii*, *T. polyphylla*, *P. ludlowii* and *P. suffruticosa* using OrthoFinder v 2.5.2 (RRID:SCR_017118) [109] to perform gene family clustering. CAFE5 (RRID:SCR_005983) [110] was used to estimate gene family expansion and contraction. To construct the phylogenetic tree, Muscle v3.8.1551 (RRID:SCR_011812) [111] was used to align single-copy genes, followed by RAxML v8.2.12 (RRID:SCR_006086) [112] to build the phylogenetic tree based on the maximum likelihood method. With the assistance of fossil records to determine the evolutionary timescale, we extracted the fourfold degenerate sites (4DTv) of each gene family and calculated the divergence time between species using the MCMCtree module in the PAML v4.9 (RRID:SCR_014932) [113]. Additionally, Ks values between paralogous gene pairs of *B. scopulosa* were calculated using the yn00 model. MCscanX (RRID:SCR_022067) [114] was used to identify collinear regions among

species, and the HKY model was used to estimate the substitution rate between species, aiding in the inference of WGD that may have occurred in the *B. scopulosa* genome.

Genome resequencing and variant calling

A total of 37 individuals were collected from nine natural populations spanning the entire distribution of the species. To ensure high-quality and completeness of sequencing, all samples were extracted with Plant DNA Kit for DNA extraction (Omega Bio-tek, Inc.), and the whole-genome paired-end sequencing was generated using DNBSEQ-T7 platform (RRID:SCR_017981), with an average sequencing depth of at least 20X. To improve data quality, the initial processing involved filtering low-quality bases, adapter sequences, duplicates, and contaminated reads using fastp v0.23.1 (RRID:SCR_016962) [115]. The clean reads were subsequently aligned to the assembled *B. scopulosa* genome via BWA v0.7.17-r1188 (RRID:SCR_010910) [116], and subsequently were sorted and converted to BAM format using SAMtools v1.16 (RRID:SCR_002105) [117]. The duplicate reads were removed with Sambamba v0.8.2 (RRID:SCR_024328) [118]. SNPs were called from the HaplotypeCaller program in GATK v4.1.4.1 (RRID:SCR_001876) [119]. Raw SNPs were filtered using VariantFiltration with filters "QD < 2.0 || MQ < 40.0 || FS > 60.0 || SOR > 3.0 || MQRankSum < -12.5 || ReadPosRankSum < -8.0". The final filtering of SNPs was accomplished using VCFtools v0.1.16 (RRID:SCR_001235) [120], with parameters "maf 0.05 max-alleles 2 min-alleles 2 minGQ 20 min-meanDP 5 max-missing 0.8".

Population genomics analyses

A SNP-based ML phylogenetic tree was constructed using RAxML-NG v1.1

(RRID:SCR_022066) [121] with GTRGAMMA substitution model and visualized by Figtree v1.4.3 (RRID:SCR_008515). The population genetic structure of the *B. scopulosa* was inferred by applying the block relaxation algorithm in ADMIXTURE v1.3.0 (RRID:SCR_001263) [122]. The parameter K ranged from 2 to 10. Principal Component Analysis (PCA) calculations were performed on the bed file generated by PLINK v1.90b6.4 (RRID:SCR_001757) [123], and the results were visualized using the ggplot2 package (RRID:SCR_014601) in R. Following population-based SNP detection in *B. scopulosa*, we employed a sliding-window strategy, utilizing 100-kb windows sliding in 10-kb increments. This allowed us to assess nucleotide diversity (π) and genetic differentiation (F_{ST}) between different lineages using VCFtools. To further investigate the population dynamics and natural selection, Tajima's D was calculated with non-overlapping sliding windows of 10-kb size. Selected candidate regions for the western and eastern groups of *B. scopulosa* were identified by taking the intersection of 5% left and right tails of the empirical $\log10_{\pi\text{-ratio}}$ (π_east/π_west) distribution and the 5% right tail of the empirical F_{ST} distribution. Subsequently, KEGG and GO annotations were performed on the genes located in these regions. Finally, the effective population size was estimated through demographic history inference using PSMC (RRID:SCR_017229) [124] and SMC++ [125], assuming a per-generation mutation rate of 2.5e-8 and a generation time of 5 years.

Identification of environmental-related genetic variations in *B. scopulosa*

To minimize false positive results, we selected 8,813,357 SNPs with a minor allele frequency (MAF) greater than 0.1 for further analysis. To evaluate the influence of

environmental variables on population differentiation of *B. scopulosa* and gain insights into the patterns of allele frequency variation along environmental gradients, we obtained 19 climate variables from the Worldclim in raster files (.asc) with a spatial resolution of 2.5 arcmin using ArcGIS v10.8 (RRID:SCR_011081, ESRI Inc., Redlands, CA, USA). After evaluating the importance ranking of 19 climate variables using the GF function in R package "gradientForest" [126], we selected five variables (BIO3, BIO4, BIO15, BIO18 and BIO19) with correlation coefficients $| r | \leq 0.75$ for further analysis. We then employed two approaches to identify the SNPs associated with climate factors. Initially, we used the "lfmm" function in R package LEA (RRID:SCR_022020) [127] to execute a univariate LFMM [128] for detecting allele frequency associations with major environmental variables. Based on the optimal genetic grouping inferred by ADMIXTURE v1.3.0, we conducted 5 independent Markov Chain Monte Carlo (MCMC) runs using 500 iterations as burn-in followed by 1000 iterations and kept SNPs with a false discovery rate (FDR) correction of $P < 0.05$. Additionally, we used a multivariate landscape genomics method known as RDA [129] to explore the correlation with climate factors. Outlier SNPs, defined as those with at least three times as many putative explanatory variables as examined, were excluded. This helped us pinpoint genetic variations tightly linked to the multivariate environmental axis. The overlapping results from both methods were considered as core "adaptive loci".

Real-time qPCR validation

To investigate the genes associated with the potential core sites for cold adaptation, we

utilized sterile seedlings of *B. scopulosa* and subjected them to a cold acclimation treatment at 4 °C for varying durations of 0, 6, 12, 24, and 48 hours. The total RNA was extracted using a FastPure Universal Plant Total RNA Isolation Kit (Vazyme, Nanjing, China). cDNA was obtained using Hifair® III 1st Strand cDNA Synthesis SuperMix, the qPCR reactions were carried out using a Hieff® qPCR SYBR Green Master Mix (No Rox) (Yeasen Biotechnology (Shanghai) Co., Ltd.) performed on the FQD-96C real-time detection system (Boer Technology). The *Bsco_actin* gene served as an internal reference, and gene-specific primers were utilized for the reactions (Supplementary Table S16). Each reaction was technically repeated three times for accuracy and reproducibility.

Analysis of gene families

To identify candidate *BsFAD* genes in the *B. scopulosa* genome, we used protein sequences of the FAD from *Arabidopsis*, wheat, rice and soybean as queries in BLAST searches (RRID:SCR_004870). The obtained sequences were then analyzed using Pfam (RRID:SCR_004726) and SMART website (RRID:SCR_005026) for structural predictions. The phylogenetic tree was constructed using LG+G model in IQ-TREE (RRID:SCR_017254) [130] through the ML method with 1000 bootstraps for inferring evolutionary relationships.

Transcriptome sequencing and analysis

Total RNA was extracted from the roots, leaves, flowers and bud from eleven fresh samples of *B. scopulosa* from XYX population in the Qinling Mountains, Shaanxi Province, China (N33°57’42”, E109°3’37”). RNA libraries were constructed for each

sample and sequenced on Illumina NovaSeq 6000 sequencing system (RRID:SCR_016387). Clean reads were aligned to the reference genome using HISAT2 v2.2.1 (RRID:SCR_015530) [131]. The R script "FeatureCounts" (RRID:SCR_012919) was used to calculate the reads counts matrix, which was then converted into FPKM and TPM values. The differential gene expression analysis was conducted using run_DE_analysis.pl script in Trinity (RRID:SCR_013048) [132].

Genomic offset of *B. scopulosa*

We used the current genotype-climate relationship and identified climate-associated genetic loci to forecast the vulnerability (genetic offset) of the *B. scopulosa* based on 19 future climatic variables (2061-2080) from WorldClim CMIP6 dataset [133] with a resolution of 2.5 arcmin of four climate models (CMCC-ESM2, EC-Earth3-Veg, GISS-E2-1-G and MIROC6). Each future environmental datasets contains two Shared socio-economic pathways (SSPs): ssp126 and ssp585. In addition, the analysis "gradientForests" [126] in R predicts genetic offset under future climate conditions across the range of *B. scopulosa*. Euclidean distance was calculated between the current and each future climate scenario to represent genetic disparity. We then averaged the values across the four future climate scenarios. A higher value indicates greater genomic vulnerability of *B. scopulosa* [134].

Additional Files

Supplementary Fig. S1. K-mer analysis of the *B. scopulosa* genome based on Illumina clean data.

Supplementary Fig. S2. Hi-C assisted assembly of *B. scopulosa* pseudochromosomes.

Heatmap showing Hi-C interactions under a resolution of 500 kb.

Supplementary Fig. S3. Genome assembly completeness evaluated based on different BUSCO groups.

Supplementary Fig. S4. Kimura distance-based copy divergence analysis of transposable elements in *B. scopulosa* genome.

Supplementary Fig. S5. Genome duplication in *B. scopulosa*.

Supplementary Fig. S6. Samples geographic distribution for *B. scopulosa*.

Supplementary Fig. S7. The distribution of π along the chromosomes among the lineages of east and west, respectively.

Supplementary Fig. S8. The distribution of F_{ST} values **(a)** and the log10π ratios (*π*_east/*π*_west) along the chromosomes in *B. scopulosa* **(b)**. Data points located to the left and right of the left and right vertical dashed lines, respectively (corresponding to the 5% left and right tails of the empirical log10π ratios distribution), and above the horizontal dashed line (the 5% right tail of the empirical F_{ST} distribution) were identified as selected regions for lineages of east (red points) and west (blue points) **(c)**.

Supplementary Fig. S9. KEGG analysis of top 5% genes under selection. Overrepresented gene ontology terms were identified using a *P*value < 0.05.

Supplementary Fig. S10. The graphs show the importance ranking of 19 environmental variables based on gradient forest analysis at SNPs (below the diagonal), and the Pearson correlation coefficient between these variables (above the diagonal). The asterisk (*) represents five highly ranked and unrelated environmental variables (Pearson's $| r | \leq 0.75$).

Supplementary Fig. S11. KEGG enrichment analysis of genes underlying the outliers using latent factor mixed model. Overrepresented gene ontology terms were identified using a *P*value < 0.05.

Supplementary Fig. S12. Redundancy analysis of five selected environmental factors response patterns in genetic variation of *B. scopulosa*.

Supplementary Fig. S13. KEGG enrichment analysis of genes underlying the outliers from RDA. Overrepresented gene ontology terms were identified using a *P*value < 0.05.

Supplementary Fig. S14. The mRNA relative expression levels at 0 h, 6 h, 12 h, 24 h and 48 h under cold treatment in *BsUGT74E2* from sterile seedling of *B. scopulosa*.

Supplementary Fig. S15. Subcellular localization of *BsUGT74E2* (*Bsco_038285*) protein in tobacco epidermal cells.

Supplementary Fig. S16. Identification of transgenic *Arabidopsis* positive seedlings by PCR. 1–10: L1, L3, L7, L9, L11, L12, L19, L20, L24, L28 transgenic *Arabidopsis*.

Supplementary Fig. S17. The relative expression of *BsUGT74E2* in transgenic *A. thaliana* strain.

Supplementary Fig. S18. Effects of *BsUGT74E2* overexpression on *Arabidopsis* seedling root length.

Supplementary Fig. S19. Allele frequencies of candidate adaptive SNPs: (a) *FAD7*, chr6_12100635, (b) *COR413pm2*, chr14_24427933, (c) *MYC2*, chr15_22548565, (d) *CRF2*, chr14_21366166 associated with BIO3 and BIO4 across the nine populations. Colors mean different alleles. N means missing alleles at leading SNP.

Supplementary Table S1. Estimation of genome size of *B. scopulosa*.

Supplementary Table S2. Summary of sequencing data of *B. scopulosa*.

Supplementary Table S3. Statistic of *B. scopulosa* genome assembly.

Supplementary Table S4. Chromosomes length of *B. scopulosa* using HiC reads.

Supplementary Table S5. Validation of genome assembly using BUSCO method with three databases.

Supplementary Table S6. The number of genes annotated for function using various methods.

Supplementary Table S7. Statistical analysis of non-coding RNAs in *B. scopulosa*.

Supplementary Table S8. Repetitive element annotations in the *B. scopulosa*.

Supplementary Table S9. Gene ontology (GO) enrichment analysis of the significant expanded genes.

Supplementary Table S10. KEGG enrichment analysis of the significant expanded genes.

Supplementary Table S11. Gene ontology (GO) enrichment analysis of the significant contracted genes.

Supplementary Table S12. KEGG enrichment analysis of the contracted genes.

Supplementary Table S13. Sample information and genome sequencing characteristics of *B. scopulosa*.

Supplementary Table S14. KEGG analysis of genomic regions exhibited high differentiation and reduced diversity between east lineage and west lineage.

Supplementary Table S15. Candidates under the outliers from genome-environment associations.

Supplementary Table S16. Sequence of primers used for qRT-PCR test under cold acclimation.

Author's Contributions

X.C. and Z.-H.L. conceived and supervised this study. X.-Y.W. collected samples. M.W. performed molecular experiments. Y.-N.Z. and J.Q. analyzed partial data. Y.-X.Y. wrote the manuscript draft. X.C. and Z.-H.L. reviewed the data and revised manuscript. All authors read and approved the final manuscript.

Funding

This work was supported by the National Natural Science Foundation of China (32470392, 31970359), Basic Research Project of Shaanxi Academy of Fundamental Science (23JHZ009, 22JHZ005), and the Key Program of Research and Development of Shaanxi Province (2022ZDLSF06–02).

Data Availability

The raw genomic sequence and RNA-seq data of *B. scopulosa* generated by this study were deposited into the NGDC (National Genomics Data Center) database under the accession number PRJCA025818 and NCBI under BioProject ID PRJNA1110036. The genome assembly, annotations, and other supporting data are available via the *GigaScience* database, GigaDB [135].

Competing Interests

All authors declare no competing interests.

Acknowledgments

We would like to express our gratitude to Assoc. Prof. Li Feng, Dr. Xin Meng, and Dr.

Ting-Ting Zhang for their guidance on data analysis.

References

1. Liang J, Crowther TW, Picard N, et al. Positive biodiversity-productivity relationship predominant in global forests. Science 2016;354. https://doi:10.1126/science.aaf8957.
2. Walther GR, Post E, Convey P, et al. Ecological responses to recent climate change. Nature 2002;416:389-95. https://doi:10.1038/416389a.
3. Derry AM, Fraser DJ, Brady SP, et al. Conservation through the lens of (mal)adaptation: Concepts and meta-analysis. Evol Appl 2019;12:1287-304. https://doi:10.1111/eva.12791.
4. Díaz S, Settele J, Brondízio ES, et al. Pervasive human-driven decline of life on Earth points to the need for transformative change. Science 2019;366:eaax3100. https://doi:10.1126/science.aax3100.
5. Savolainen O, Lascoux M, Merilä J. Ecological genomics of local adaptation. Nat Rev Genet 2013;14:807-20. https://doi:10.1038/nrg3522.
6. Sork VL. Genomic studies of local adaptation in natural plant populations. J Hered. 2017;109:3-15. https://doi:10.1093/jhered/esx091.
7. Delph LF. The study of local adaptation: a thriving field of research. J Hered. 2018;109:1-2. https://doi:10.1093/jhered/esx099.
8. Bay RA, Harrigan RJ, Underwood VL, et al. Genomic signals of selection predict climate-driven population declines in a migratory bird. Science. 2018;359:83-6. https://doi:10.1126/science.aan4380.
9. Ruegg K, Bay RA, Anderson EC, et al. Ecological genomics predicts climate vulnerability in an endangered southwestern songbird. Ecol Lett. 2018;21:1085-96. https://doi:10.1111/ele.12977.
10. Rhoné B, Defrance D, Berthouly-Salazar C, et al. Pearl millet genomic vulnerability to climate change in West Africa highlights the need for regional collaboration. Nat Commun. 2020;11:5274. https://doi:10.1038/s41467-020-19066-4.
11. Tournebize R, Borner L, Manel S, et al. Ecological and genomic vulnerability to climate change across native populations of Robusta coffee (*Coffea canephora*). Glob Chang Biol 2022;28:4124-42. https://doi:10.1111/gcb.16191.
12. Mays HL, Hung CM, Shaner PJ, et al. Genomic analysis of demographic history and ecological niche modeling in the endangered Sumatran rhinoceros *Dicerorhinus sumatrensis*. Curr Biol 2018;28:70-6. https://doi:10.1016/j.cub.2017.11.021.
13. Saunders SP, Michel NL, Bateman BL, et al. Community science validates climate suitability projections from ecological niche modeling. Ecol Appl 2020;30:e02128. https://doi:10.1002/eap.2128.
14. Chiarenza AA, Waterson AM, Schmidt DN, et al. 100 million years of turtle paleoniche dynamics enable the prediction of latitudinal range shifts in a warming world. Curr Biol 2023;33:109-21.e3. https://doi:10.1016/j.cub.2022.11.056.
15. Gandia AC, Bosch RA, Mancina CA, et al. Climatic variation along the distributional range in Cuban *Anolis* lizards: Species and ecomorphs under future scenarios of climate change. Glob Ecol Conserv 2023;42:e02401. https://doi:10.1016/j.gecco.2023.e02401.
16. Cao YN, Zhu SS, Chen J, et al. Genomic insights into historical population dynamics, local adaptation, and climate change vulnerability of the East Asian Tertiary relict *Euptelea*

(Eupteleaceae). Evol Appl 2020;13:2038-55. https://doi:10.1111/eva.12960.

17. Zhao W, Sun YQ, Pan J, et al. Effects of landscapes and range expansion on population structure and local adaptation. New Phytol 2020;228:330-43. https://doi:10.1111/nph.16619.

18. Guo JF, Zhao W, Andersson B, et al. Genomic clines across the species boundary between a hybrid pine and its progenitor in the eastern Tibetan Plateau. Plant Commun 2023;4:100574. https://doi:10.1016/j.xplc.2023.100574.

19. Yuan S, Shi Y, Zhou BF, et al. Genomic vulnerability to climate change in *Quercus acutissima*, a dominant tree species in East Asian deciduous forests. Mol Ecol 2023;7:1639-55. https://doi:10.1111/mec.16843.

20. Rellstab C, Zoller S, Walthert L, et al. Signatures of local adaptation in candidate genes of oaks (*Quercus* spp.) with respect to present and future climatic conditions. Mol Ecol 2016;25:5907-24. https://doi:10.1111/mec.13889.

21. Capblancq T, Fitzpatrick MC, Bay RA, et al. Genomic prediction of (mal)adaptation across current and future climatic landscapes. Annu Rev Ecol Evol Syst 2020;51:245-69. https://doi:10.1146/annurev-ecolsys-020720-042553.

22. Hoffmann AA, Weeks AR, Sgrò CM. Opportunities and challenges in assessing climate change vulnerability through genomics. Cell 2021;184:1420-25. https://doi:10.1016/j.cell.2021.02.006.

23. Feng L, Du FK. Landscape genomics in tree conservation under a changing environment. Front Plant Sci 2022;13:822217. https://doi:10.3389/fpls.2022.822217.

24. Shi T, Zhang X, Hou Y, et al. The super-pangenome of *Populus* unveils genomic facets for its adaptation and diversification in widespread forest trees. Mol Plant 2024;17:725-46. https://doi:10.1016/j.molp.2024.03.009.

25. Sang Y, Long Z, Dan X, et al. Genomic insights into local adaptation and future climate-induced vulnerability of a keystone forest tree in East Asia. Nat Commun 2022;13:6541. https://doi:10.1038/s41467-022-34206-8.

26. Zhang F, Long R, Ma Z, et al. Evolutionary genomics of climatic adaptation and resilience to climate change in alfalfa. Mol Plant 2024;17:867-83. https://doi:10.1016/j.molp.2024.04.013.

27. Pan JT, Soltis DE. BERGENIA Moench. Flora of China. Beijing: Science Press; 2001: p. 278-80.

28. Sánchez-Pujante PJ, Borja-Martínez M, Pedreño MA, et al. Biosynthesis and bioactivity of glucosinolates and their production in plant in vitro cultures. Planta 2017;246:19-32. https://doi:10.1007/s00425-017-2705-9.

29. Gong Z, Xiong L, Shi H, et al. Plant abiotic stress response and nutrient use efficiency. Sci China Life Sci 2020;63:635-74. https://doi:10.1007/s11427-020-1683-x.

30. Zhang H, Zhao Y, Zhu JK. Thriving under stress: how plants balance growth and the stress response. Dev Cell 2020;55:529-43. https://doi:10.1016/j.devcel.2020.10.012.

31. Dong NQ, Lin HX. Contribution of phenylpropanoid metabolism to plant development and plant-environment interactions. J Integr Plant Biol 2021;63:180-209. https://doi:10.1111/jipb.13054.

32. Wang P, Jin S, Chen X, et al. Chromatin accessibility and translational landscapes of tea plants under chilling stress. Hortic Res 2021;8:96. https://doi:10.1038/s41438-021-00529-8.

33. Dedyukhina EG, Kamzolova SV, Vainshtein MB. Arachidonic acid as an elicitor of the plant defense response to phytopathogens. Chem Biol Technol Agric 2014;1:18. https://doi:10.1186/s40538-014-0018-9.

34. Deng B, Jin X, Yang Y, et al. The regulatory role of riboflavin in the drought tolerance of tobacco plants depends on ROS production. Plant Growth Regul 2014;72:269-77.

https://doi:10.1007/s10725-013-9858-8.
35. Guhr A, Horn MA, Weig AR. Vitamin B_2 (riboflavin) increases drought tolerance of *Agaricus bisporus*. Mycologia 2017;109:860-73. https://doi:10.1080/00275514.2017.1414544.
36. Jamar NH, Kritsiligkou P, Grant CM. The non-stop decay mRNA surveillance pathway is required for oxidative stress tolerance. Nucleic Acids Res 2017;45:6881-93. https://doi:10.1093/nar/gkx306.
37. Muthuramalingam P, Krishnan SR, Pandian S, et al. Global analysis of threonine metabolism genes unravel key players in rice to improve the abiotic stress tolerance. Sci Rep 2018;8:9270. https://doi:10.1038/s41598-018-27703-8.
38. Zhao H, Ke H, Zhang L, et al. Integrated analysis about the effects of heat stress on physiological responses and energy metabolism in *Gymnocypris chilianensis*. Sci Total Environ 2022;806:151252. https://doi:10.1016/j.scitotenv.2021.151252.
39. Zhu F, Cao MY, Zhang QP, et al. Join the green team: Inducers of plant immunity in the plant disease sustainable control toolbox. J Adv Res 2024;57:15-42. https://doi:10.1016/j.jare.2023.04.016.
40. Ren H, Gray WM. SAUR proteins as effectors of hormonal and environmental signals in plant growth. Mol Plant 2015;8:1153-64. https://doi:10.1016/j.molp.2015.05.003.
41. Gao J, Zhang R, Zheng L, et al. Blue light receptor CRY1 regulates HSFA1d nuclear localization to promote plant thermotolerance. Cell Rep 2023;42:113117. https://doi:10.1016/j.celrep.2023.113117.
42. Wang F, Guo Z, Li H, et al. Phytochrome A and B function antagonistically to regulate cold tolerance via abscisic acid-dependent jasmonate signaling. Plant Physiol 2016;170:459-71. https://doi.org/10.1104/pp.15.01171.
43. Aghaie P, Tafreshi SAH. Central role of 70-kDa heat shock protein in adaptation of plants to drought stress. Cell Stress Chaperones 2020;25:1071-81. https://doi:10.1007/s12192-020-01144-7.
44. Hemsley PA, Hurst CH, Kaliyadasa E, et al. The *Arabidopsis* mediator complex subunits MED16, MED14, and MED2 regulate mediator and RNA polymerase II recruitment to CBF-responsive cold-regulated genes. Plant Cell 2014;26:465-84. https://doi:10.1105/tpc.113.117796.
45. Ming R, Zhang Y, Wang Y, et al. The JA-responsive MYC2-*BADH-like* transcriptional regulatory module in *Poncirus trifoliata* contributes to cold tolerance by modulation of glycine betaine biosynthesis. New Phytol 2021;229:2730-50. https://doi:10.1111/nph.17063.
46. Wang R, Yu M, Xia J, et al. Overexpression of *TaMYC2* confers freeze tolerance by ICE-CBF-COR module in *Arabidopsis thaliana*. Front Plant Sci 2022;13:1042889. https://doi:10.3389/fpls.2022.1042889.
47. Zhang D, Zhao Y, Wang J, et al. BRS1 mediates plant redox regulation and cold responses. BMC Plant Biol 2021;21:268. https://doi:10.1186/s12870-021-03045-y.
48. Jeon J, Cho C, Lee MR, et al. *CYTOKININ RESPONSE FACTOR2* (*CRF2*) and *CRF3* regulate lateral root development in response to cold stress in Arabidopsis. Plant Cell 2016;28:1828-43. https://doi:10.1105/tpc.15.00909.
49. Pacifici M, Foden WB, Visconti P, et al. Assessing species vulnerability to climate change. Nature Clim Change 2015;5:215-24. https://doi:10.1038/nclimate2448.
50. Foden WB, Young BE, Akçakaya HR, et al. Climate change vulnerability assessment of species. Wiley Interdiscip Rev Clim Change 2019;10:e551. https://doi:10.1002/wcc.551.
51. Schlaepfer MA, Lawler JJ. Conserving biodiversity in the face of rapid climate change requires a shift in priorities. Wiley Interdiscip Rev Clim Change 2023;14:e798.

https://doi.org/10.1002/wcc.798.

52. Zhu G, Giam X, Armsworth PR, et al. Biodiversity conservation adaptation to climate change: protecting the actors or the stage. Ecol Appl 2023;33:e2765. https://doi:10.1002/eap.2765.

53. Bonebrake TC, Guo F, Dingle C, et al. Integrating proximal and horizon threats to biodiversity for conservation. Trends Ecol Evol 2019;34:781-8. https://doi:10.1016/j.tree.2019.04.001.

54. Crane P. Conserving our global botanical heritage: The PSESP plant conservation program. Plant Divers 2020;42:319-22. https://doi:10.1016/j.pld.2020.06.007.

55. Shi X, Cao S, Wang X, et al. The complete reference genome for grapevine (*Vitis vinifera* L.) genetics and breeding. Hortic Res 2023;10:uhad061. https://doi:10.1093/hr/uhad061.

56. Huang HR, Liu X, Arshad R, et al. Telomere-to-telomere haplotype-resolved reference genome reveals subgenome divergence and disease resistance in triploid Cavendish banana. Hortic Res 2023;10:uhad153. https://doi:10.1093/hr/uhad153.

57. Song Y, Peng Y, Liu L, et al. Phased gap-free genome assembly of octoploid cultivated strawberry illustrates the genetic and epigenetic divergence among subgenomes. Hortic Res 2024;11:uhad252. https://doi:10.1093/hr/uhad252.

58. Su Y, Yang X, Wang Y, et al. Phased telomere-to-telomere reference genome and pangenome reveal an expansion of resistance genes during apple domestication. Plant Physiol 2024;195:2799-814. https://doi:10.1093/plphys/kiae258.

59. Zachos J, Pagani M, Sloan L, et al. Trends, rhythms, and aberrations in global climate 65 Ma to present. Science 2001;292:686-93. https://doi:10.1126/science.1059412.

60. Wei J, Liu H, Zhao Y, et al. Simulation of the climate and ocean circulations in the Middle Miocene Climate Optimum by a coupled model FGOALS-g3. Palaeogeogr Palaeocl 2023;617:111509. https://doi.org/10.1016/j.palaeo.2023.111509.

61. Ji L, Zhang M, Song Z. The palynological record from Coniacian to lower Campanian continental sequences in the Songliao Basin, northeastern China and its implications for palaeoclimate. Cretac Res 2015;56:226-36. https://doi.org/10.1016/j.cretres.2015.04.006.

62. Qi G, Song J, Li Q, et al. Response of vegetation to multi-timescales drought in the Qinling Mountains of China. Ecol Indic 2022;135:108539. https://doi:10.1016/j.ecolind.2022.108539.

63. Kim E, Donohue K. Local adaptation and plasticity of *Erysimum capitatum* to altitude: its implications for responses to climate change. J Ecol 2013;101:796-805. https://doi:10.1111/1365-2745.12077.

64. Liang Q, Xu X, Mao K, et al. Shifts in plant distributions in response to climate warming in a biodiversity hotspot, the Hengduan Mountains. J Biogeogr 2018;45:1334-44. https://doi:10.1111/jbi.13229.

65. Theocharis A, Clément C, Barka EA. Physiological and molecular changes in plants grown at low temperatures. Planta 2012;235:1091-105. https://doi:10.1007/s00425-012-1641-y.

66. Richardson AD, Hufkens K, Milliman T, et al. Ecosystem warming extends vegetation activity but heightens vulnerability to cold temperatures. Nature 2018;560:368-71. https://doi:10.1038/s41586-018-0399-1.

67. Vyse K, Pagter M, Zuther E, et al. Deacclimation after cold acclimation-a crucial, but widely neglected part of plant winter survival. J Exp Bot 2019;70:4595-604. https://doi:10.1093/jxb/erz229.

68. Liu B, Wang XY, Cao Y, et al. Factors affecting freezing tolerance: a comparative transcriptomics study between field and artificial cold acclimations in overwintering evergreens. Plant J 2020;103:2279-300. https://doi:10.1111/tpj.14899.

69. Liu B, Zhao FM, Cao Y, et al. Photoprotection contributes to freezing tolerance as revealed by RNA-seq profiling of *Rhododendron* leaves during cold acclimation and deacclimation over time. Hortic Res 2022;9:uhab025. https://doi:10.1093/hr/uhab025.
70. Rellstab C, Gugerli F, Eckert AJ, et al. A practical guide to environmental association analysis in landscape genomics. Mol Ecol 2015;24:4348-70. https://doi:10.1111/mec.13322.
71. Forester BR, Lasky JR, Wagner HH, et al. Comparing methods for detecting multilocus adaptation with multivariate genotype-environment associations. Mol Ecol 2018;27:2215-33. https://doi:10.1111/mec.14584.
72. Capblancq T, Luu K, Blum MG, et al. Evaluation of redundancy analysis to identify signatures of local adaptation. Mol Ecol Resour 2018;18:1223-33. https://doi:10.1111/1755-0998.12906.
73. Cao S, Ye M, Jiang S. Involvement of *GIGANTEA* gene in the regulation of the cold stress response in *Arabidopsis*. Plant Cell Rep 2005;24:683-90. https://doi:10.1007/s00299-005-0061-x.
74. Kurepa J, Smalle J, Van Montagu M, et al. Oxidative stress tolerance and longevity in *Arabidopsis*: the late-flowering mutant *gigantea* is tolerant to paraquat. Plant J 1998;14:759-64. https://doi:10.1046/j.1365-313x.1998.00168.x.
75. Mishra P, Panigrahi KC. GIGANTEA - an emerging story. Front Plant Sci 2015;6:8. https://doi:10.3389/fpls.2015.00008.
76. Iba K. Acclimative response to temperature stress in higher plants: approaches of gene engineering for temperature tolerance. Annu Rev Plant Biol 2002;53:225-45. https://doi:10.1146/annurev.arplant.53.100201.160729.
77. Soria-García Á, Rubio MC, Lagunas B, et al. Tissue distribution and specific contribution of *Arabidopsis* FAD7 and FAD8 plastid desaturases to the JA- and ABA-mediated cold stress or defense responses. Plant cell physiology 2019;60:1025-40. https://doi.org/10.1093/pcp/pcz017.
78. Gopalakrishnan Nair PM, Kang I, Moon B, et al. Effects of low temperature stress on rice (*Oryza sativa* L.) plastid ω-3 desaturase gene, *OsFAD8* and its functional analysis using T-DNA mutants. Plant Cell Tiss Organ Cult 2009;98:87-96. https://doi:10.1007/s11240-009-9541-y.
79. Khodakovskaya M, Mcavoy R, Peters J, et al. Enhanced cold tolerance in transgenic tobacco expressing a chloroplast ω-3 fatty acid desaturase gene under the control of a cold-inducible promoter. Planta 2006;223:1090-100. https://doi:10.1007/s00425-005-0161-4.
80. Tognetti VB, Van Aken O, Morreel K, et al. Perturbation of indole-3-butyric acid homeostasis by the UDP-glucosyltransferase *UGT74E2* modulates *Arabidopsis* architecture and water stress tolerance. Plant Cell 2010;22:2660-79. https://doi:10.1105/tpc.109.071316.
81. Gao H, Lü X, Ren W, et al. *HaASR1* gene cloned from a desert shrub, *Haloxylon ammodendron*, confers drought tolerance in transgenic *Arabidopsis thaliana*. Environ Exp Bot 2020;180:104251. https://doi:10.1016/j.envexpbot.2020.104251.
82. Wang T, Li P, Mu T, et al. Overexpression of *UGT74E2*, an Arabidopsis IBA Glycosyltransferase, enhances seed germination and modulates stress tolerance via ABA signaling in Rice. Int J Mol Sci 2020;21:7239. https://doi:10.3390/ijms21197239.
83. Jiang M, Ma LL, Huang HA, et al. Overexpression of *SgGH3.1* from fine-stem stylo (*Stylosanthes guianensis* var. *intermedia*) enhances chilling and cold tolerance in *Arabidopsis thaliana*. Genes (Basel) 2021;12:1367. https://doi:10.3390/genes12091367.
84. Korver RA, Koevoets IT, Testerink C. Out of shape during stress: a key role for auxin. Trends Plant Sci 2018;23:783-93. https://doi:10.1016/j.tplants.2018.05.011.
85. Jaglo-Ottosen KR, Gilmour SJ, Zarka DG, et al. *Arabidopsis CBF1* overexpression induces *COR*

genes and enhances freezing tolerance. Science 1998;280:104-6. https://doi:10.1126/science.280.5360.104.

86. Ding Y, Shi Y, Yang S. Advances and challenges in uncovering cold tolerance regulatory mechanisms in plants. New Phytol 2019;222:1690-704. https://doi:10.1111/nph.15696.

87. Liu Y, Dang P, Liu L, et al. Cold acclimation by the CBF–*COR* pathway in a changing climate: lessons from *Arabidopsis thaliana*. Plant Cell Rep 2019;38:511-9. https://doi:10.1007/s00299-019-02376-3.

88. Ma H, Liu Y, Liu D, et al. Chromosome-level genome assembly and population genetic analysis of a critically endangered rhododendron provide insights into its conservation. Plant J 2021;107:1533-45. https://doi:10.1111/tpj.15399.

89. Li R, Li Y, Kristiansen K, et al. SOAP: short oligonucleotide alignment program. Bioinformatics 2008;24:713-4. https://doi:10.1093/bioinformatics/btn025.

90. Li R, Zhu H, Ruan J, et al. De novo assembly of human genomes with massively parallel short read sequencing. Genome Res 2010;20:265-72. https://doi:10.1101/gr.097261.109.

91. Cheng H, Concepcion GT, Feng X, et al. Haplotype-resolved de novo assembly using phased assembly graphs with hifiasm. Nat Methods 2021;18:170-5. https://doi:10.1038/s41592-020-01056-5.

92. Hu J, Fan J, Sun Z, et al. NextPolish: a fast and efficient genome polishing tool for long-read assembly. Bioinformatics 2020;36:2253-5. https://doi:10.1093/bioinformatics/btz891.

93. Servant N, Varoquaux N, Lajoie BR, et al. HiC-Pro: an optimized and flexible pipeline for Hi-C data processing. Genome Biol 2015;16:259. https://doi:10.1186/s13059-015-0831-x.

94. Langmead B, Salzberg SL. Fast gapped-read alignment with Bowtie 2. Nat Methods 2012;9:357-9. https://doi:10.1038/nmeth.1923.

95. Burton JN, Adey A, Patwardhan RP, et al. Chromosome-scale scaffolding of *de novo* genome assemblies based on chromatin interactions. Nat Biotechnol 2013;31:1119-25. https://doi:10.1038/nbt.2727.

96. Simão FA, Waterhouse RM, Ioannidis P, et al. BUSCO: assessing genome assembly and annotation completeness with single-copy orthologs. Bioinformatics 2015;31:3210-2. https://doi:10.1093/bioinformatics/btv351.

97. Zhi D, Raphael BJ, Price AL, et al. Identifying repeat domains in large genomes. Genome Biol 2006;7:R7. https://doi:10.1186/gb-2006-7-1-r7.

98. Levitsky VG. RECON: a program for prediction of nucleosome formation potential. Nucleic Acids Res 2004;32:W346-9. https://doi:10.1093/nar/gkh482.

99. Price AL, Jones NC, Pevzner PA. *De novo* identification of repeat families in large genomes. Bioinformatics 2005;21:i351-8. https://doi:10.1093/bioinformatics/bti1018.

100. Stanke M, Keller O, Gunduz I, et al. AUGUSTUS: ab initio prediction of alternative transcripts. Nucleic Acids Res 2006;34:W435-9. https://doi:10.1093/nar/gkl200.

101. Hoff KJ, Lomsadze A, Borodovsky M, et al. Whole-Genome Annotation with BRAKER. Methods Mol Biol 2019;1962:65-95. https://doi:10.1007/978-1-4939-9173-0_5.

102. Cantarel BL, Korf I, Robb SM, et al. MAKER: An easy-to-use annotation pipeline designed for emerging model organism genomes. Genome Res 2008;18:188-96. https://doi:10.1101/gr.6743907.

103. Kanehisa M, Goto S. KEGG: kyoto encyclopedia of genes and genomes. Nucleic Acids Res 2000;28:27-30. https://doi:10.1093/nar/28.1.27.

104. Conesa A, Gotz S, Garcia-Gomez JM, et al. Blast2GO: a universal tool for annotation, visualization

and analysis in functional genomics research. Bioinformatics 2005;21:3674-6. https://doi:10.1093/bioinformatics/bti610.

105. Huerta-Cepas J, Szklarczyk D, Heller D, et al. eggNOG 5.0: a hierarchical, functionally and phylogenetically annotated orthology resource based on 5090 organisms and 2502 viruses. Nucleic Acids Res 2019;47:D309-14. https://doi:10.1093/nar/gky1085.

106. Chan PP, Lin BY, Mak AJ, et al. tRNAscan-SE 2.0: improved detection and functional classification of transfer RNA genes. Nucleic Acids Res 2021;49:9077-96. https://doi:10.1093/nar/gkab688.

107. Nawrocki EP, Eddy SR. Infernal 1.1: 100-fold faster RNA homology searches. Bioinformatics 2013;29:2933-5. https://doi:10.1093/bioinformatics/btt509.

108. Griffiths-Jones S, Moxon S, Marshall M, et al. Rfam: annotating non-coding RNAs in complete genomes. Nucleic Acids Res 2005;33:D121-4. https://doi:10.1093/nar/gki081.

109. Emms DM, Kelly S. OrthoFinder: phylogenetic orthology inference for comparative genomics. Genome Biol 2019;20:238. https://doi:10.1186/s13059-019-1832-y.

110. Mendes FK, Vanderpool D, Fulton B, et al. CAFE 5 models variation in evolutionary rates among gene families. Bioinformatics 2021;36:5516-8. https://doi:10.1093/bioinformatics/btaa1022.

111. Edgar RC. MUSCLE: multiple sequence alignment with high accuracy and high throughput. Nucleic Acids Res 2004;32:1792-7. https://doi:10.1093/nar/gkh340.

112. Stamatakis A. RAxML version 8: a tool for phylogenetic analysis and post-analysis of large phylogenies. Bioinformatics 2014;30:1312-3. https://doi:10.1093/bioinformatics/btu033.

113. Yang Z. PAML 4: phylogenetic analysis by maximum likelihood. Mol Biol Evol 2007;24:1586-91. https://doi:10.1093/molbev/msm088.

114. Wang Y, Tang H, Debarry JD, et al. MCScanX: a toolkit for detection and evolutionary analysis of gene synteny and collinearity. Nucleic Acids Res 2012;40:e49. https://doi:10.1093/nar/gkr1293.

115. Chen S, Zhou Y, Chen Y, et al. fastp: an ultra-fast all-in-one FASTQ preprocessor. Bioinformatics 2018;34:i884-90. https://doi:10.1093/bioinformatics/bty560.

116. Li H. Aligning sequence reads, clone sequences and assembly contigs with BWA-MEM. ArXiv 2013. https://doi:10.48550/arXiv.1303.3997.

117. Danecek P, Bonfield JK, Liddle J, et al. Twelve years of SAMtools and BCFtools. Gigascience 2021;10:giab008. https://doi:10.1093/gigascience/giab008.

118. Tarasov A, Vilella AJ, Cuppen E, et al. Sambamba: fast processing of NGS alignment formats. Bioinformatics 2015;31:2032-4. https://doi:10.1093/bioinformatics/btv098.

119. Mckenna A, Hanna M, Banks E, et al. The Genome Analysis Toolkit: a MapReduce framework for analyzing next-generation DNA sequencing data. Genome Res 2010;20:1297-303. https://doi:10.1101/gr.107524.110.

120. Danecek P, Auton A, Abecasis G, et al. The variant call format and VCFtools. Bioinformatics 2011;27:2156-8. https://doi:10.1093/bioinformatics/btr330.

121. Kozlov AM, Darriba D, Flouri T, et al. RAxML-NG: a fast, scalable and user-friendly tool for maximum likelihood phylogenetic inference. Bioinformatics 2019;35:4453-5. https://doi:10.1093/bioinformatics/btz305.

122. Alexander DH, Novembre J, Lange K. Fast model-based estimation of ancestry in unrelated individuals. Genome Res 2009;19:1655-64. https://doi:10.1101/gr.094052.109.

123. Purcell S, Neale B, Todd-Brown K, et al. PLINK: a tool set for whole-genome association and population-based linkage analyses. Am J Hum Genet 2007;81:559-75. https://doi:10.1086/519795.

124. Li H, Durbin R. Inference of human population history from individual whole-genome sequences.

Nature 2011;475:493-6. https://doi:10.1038/nature10231.

125. Terhorst J, Kamm JA, Song YS. Robust and scalable inference of population history from hundreds of unphased whole genomes. Nat Genet 2017;49:303-9. https://doi:10.1038/ng.3748.

126. Ellis N, Smith SJ, Pitcher CR. Gradient forests: calculating importance gradients on physical predictors. Ecology 2012;93:156-68. https://doi:10.1890/11-0252.1.

127. Frichot E, François O. LEA: An R package for landscape and ecological association studies. Methods Ecol Evol 2015;6:925-9. https://doi:10.1111/2041-210X.12382.

128. Frichot E, Schoville SD, Bouchard G, et al. Testing for associations between loci and environmental gradients using latent factor mixed models. Mol Biol Evol 2013;30:1687-99. https://doi:10.1093/molbev/mst063.

129. Capblancq T, Forester BR. Redundancy analysis: a swiss army knife for landscape genomics. Methods Ecol Evol 2021;12:2298-309. https://doi.org/10.1111/2041-210X.13722.

130. Minh BQ, Schmidt HA, Chernomor O, et al. IQ-TREE 2: new models and efficient methods for phylogenetic inference in the genomic era. Mol Biol Evol 2020;37:1530-4. https://doi:10.1093/molbev/msaa015.

131. Kim D, Paggi JM, Park C, et al. Graph-based genome alignment and genotyping with HISAT2 and HISAT-genotype. Nat Biotechnol 2019;37:907-15. https://doi:10.1038/s41587-019-0201-4.

132. Haas BJ, Papanicolaou A, Yassour M, et al. De novo transcript sequence reconstruction from RNA-seq using the Trinity platform for reference generation and analysis. Nat Protoc 2013;8:1494-512. https://doi:10.1038/nprot.2013.084.

133. Eyring V, Bony S, Meehl GA, et al. Overview of the Coupled Model Intercomparison Project Phase 6 (CMIP6) experimental design and organization. Geosci Model Dev 2016;9:1937-58. https://doi:10.5194/gmd-9-1937-2016.

134. Fitzpatrick MC, Keller SR. Ecological genomics meets community-level modelling of biodiversity: mapping the genomic landscape of current and future environmental adaptation. Ecol Lett 2015;18:1-16. https://doi:10.1111/ele.12376.

135. Yang Y, Wang M, Wu X, et al. Supporting data for "The chromosome-level genome assembly of an endangered herb Bergenia scopulosa provides insights into local adaptation and genomic vulnerability under climate change" GigaScience Database. 2024. https://doi.org/10.5524/102589.

Table 1. Statistic of *B. scopulosa* genome assembly and annotation

Feature	Statistic
Assembled genome size (bp)	733,357,027
GC content (%)	36
Contig Number	1083
Contig N50 (bp)	20,841,689
Scaffold Number	757
Scaffold N50 (bp)	37,958,852
Minimum Len (bp)	17547
Maximum Len (bp)	73,384,106
Mean Len (bp)	968,768
Median Len (bp)	36,832
Number of annotated genes	45,222
Repeats in genome (%)	67.36
Average BUSCO (complete) (%)	99.1

Figure legends

Fig. 1. Habitat, morphological and genomic characteristics of *B. scopulosa*.

(a) Typical habitat of *B. scopulosa* growing in the crevices of cliffs and rocks in the TYP population.

(b) Individual wild *B. scopulosa* in TBS population.

(c) *Flowers of B. scopulosa*.

(d) Overview of the *B. scopulosa* draft genome assembly. **(i)** The 17 assembled *B. scopulosa* chromosomes; **(ii)** The Gene count along the genome; **(iii)** The repetitive sequences density along the genome; **(iv)** The GC content along the genome; **(v)** Syntenic relationships among different chromosomes of *B. scopulosa*.

Fig. 2. Comparative genomic analysis of *B. scopulosa* and its related species.

(a) Phylogenetic tree representing the number of gene families that have expanded or contracted among ten species. The pie charts show the percentage of expanded (purple), contracted (red) and conserved (blue) gene families across all gene families. The estimated divergence time (in millions of years) is shown beside the branch nodes in blue, the numbers enclosed in parentheses indicate the confidence interval for the estimated divergence time. The scale on the x axis shows the estimated divergence time for nodes. "+" indicates that gene families expanded, "–" indicates that gene families contracted.

(b) Number of paralogous gene families among ten species.

(c) Kernel-density estimates of Ks distributions for one-to-one orthologs (reciprocal best-hits) among *B. scopulosa*, *K. fedtschenkoi* and *T. polyphylla*. Bsco stands for *B. scopulosa*, BsKf stands for *B. scopulosa*-*K. fedtschenkoi*, BsTp stands for *B. scopulosa*-*T. polyphylla*, Kfed stands for *K. fedtschenkoi* and Tpol stands for *T. polyphylla*.

Fig. 3. Population structure, genetic diversity, and demographic history of *B. scopulosa*.

(a) Admixture analysis with individual ancestry coefficients $K = 2,3$.

(b) ADMIXTURE cross validation errors for each K value (2-10). The $K = 3$ is the optimal.

(c) Spatial genetic structure of *B. scopulosa* based on nine populations within the natural species range in China. The pie chart colors indicate the probability of sample assignment based on SNPs analyzed using ADMIXTURE at K= 2 and 3.
(d) Principal component analysis (PCA) of single nucleotide polymorphisms (SNPs) identified from the re-sequenced individuals. The first two principal components (PC1, 12.33% vs. PC2, 9.03%) are shown.
(e) Non-rooted maximum-likelihood (ML) phylogenetic tree based on SNPs in 37 individuals. Color coding of the branches reflects the structure of genetic groups at K = 2.
(f) Past effective population size history of *B. scopulosa* assessed by PSMC.
(g Demographic changes on recent timescales established for west and east lineages by using SMC++.

Fig. 4. Genome-wide screening of the freezing-tolerance-related loci with local adaptation.
(a) The mRNA relative expression levels at 0 h, 6 h, 12 h, 24 h, 48 h under cold treatment in eight selected-genes from sterile seedling of *B. scopulosa*. Data are presented as mean ± SE (n = 3). "*" and "**" indicate a significant difference from that of 0 h at $P \leq 0.05$ and $P \leq 0.01$, respectively, by student's t-test.
(b) The $\log_{10}$ (FPKM) expression values of eight candidate genes are represented by a color heatmap ranging from blue to red across four tissues: bud, flower, leaf, and root. The color gradient ranging from blue to red indicates low to high expression levels.
(c) Phylogenetic relationships of *FAD* genes from *B. scopulosa* (Bs), *Arabidopsis* (At), wheat (Ta), rice (Os) and soybean (Gm). The colored branch shows a different subfamily. The tree was constructed using IQ-tree software by the maximum-likelihood (ML) method with 1000 bootstraps and the pale red dots in the figure indicate the bootstrap value.
(d) Phylogenetic relationships and expression heatmap in different tissues of 27 *FAD* genes from *B. scopulosa*. The *BsFAD7* (*Bsco_006378*) gene, highlighted in red, has been validated in this study.

Fig. 5. Predicted genetic offset of SNPs across *B. scopulosa* distribution in the future. **(a, c**) ssp_126 and ssp_585 scenarios in 2061-2080, lighter hues indicate higher genetic offset (higher expected vulnerability to climate change). The circle signifies each population, and the hue denotes whether they pertain to the chilly western lineage (depicted in blue) or the warm eastern lineage (depicted in red). The mapped area is the Qinling Mountains region within Shaanxi Province, China. **(b, d)** Estimated genetic offsets combined across all populations in two lineages.

Figure 1

Click here to access/download;Figure;Fig1.pdf

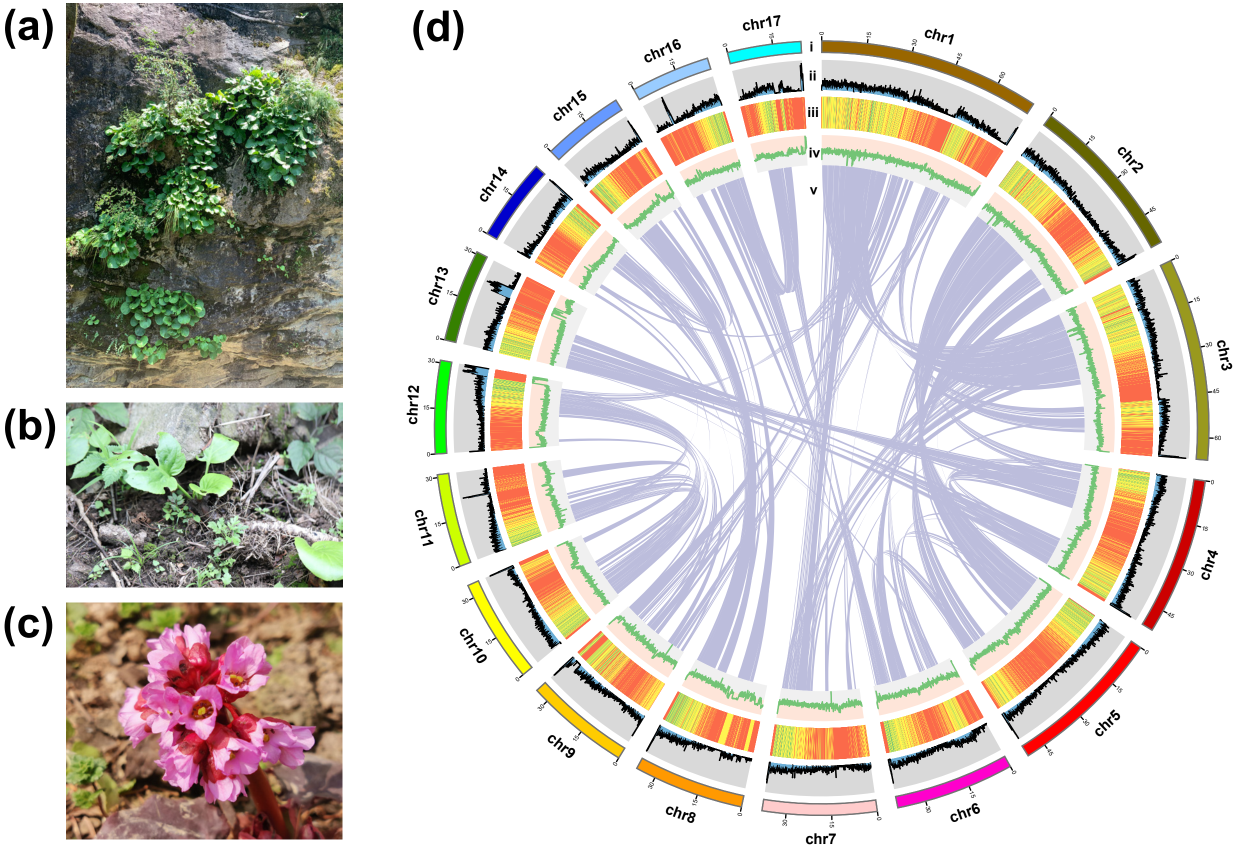

Figure 2

Click here to access/download;Figure;Fig2.pdf

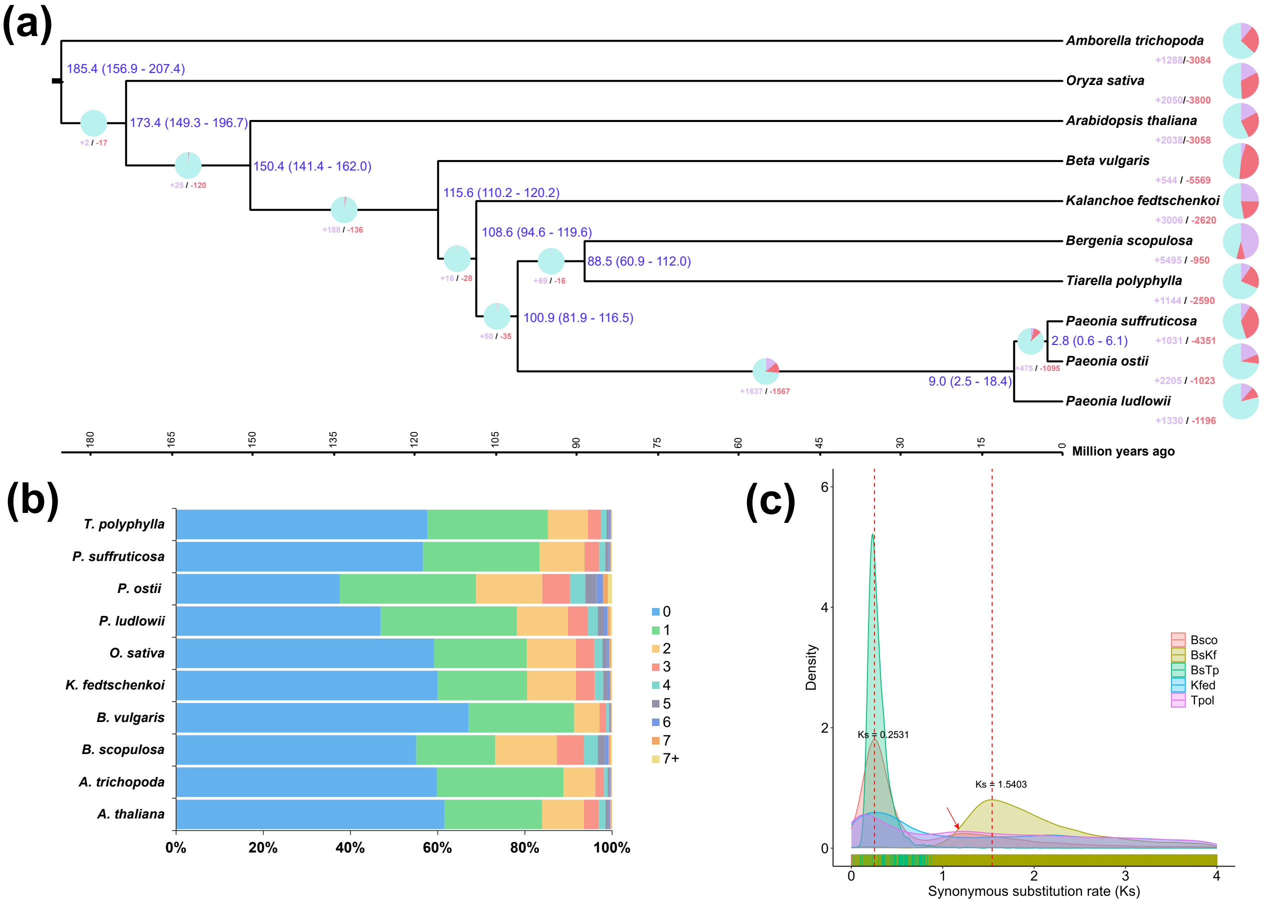

Figure 3

Click here to access/download;Figure;Fig.3.pdf

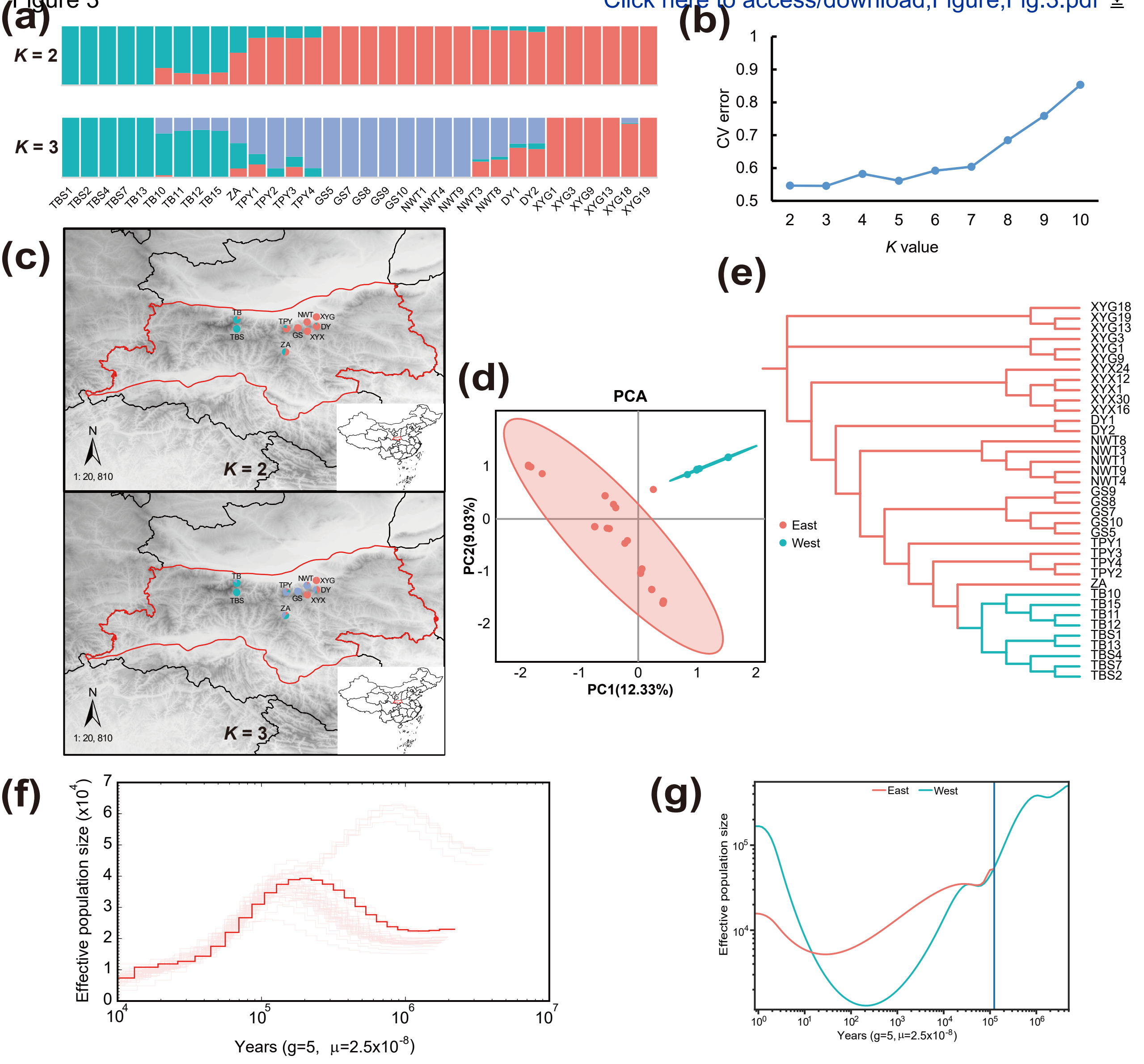

Figure 4

Click here to access/download;Figure;Fig4.pdf

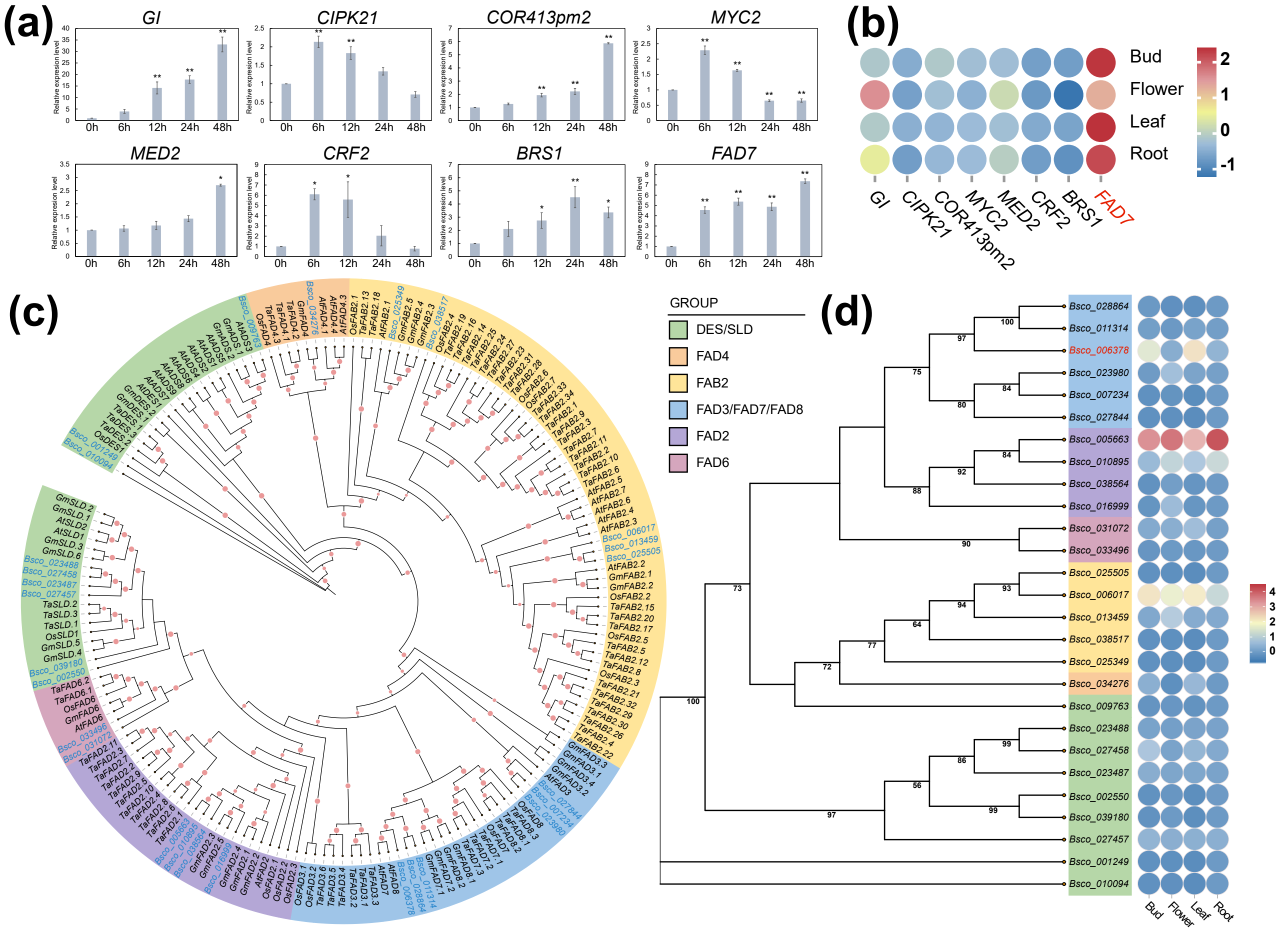

Click here to access/download;Figure;Fig5.pdf

Figure 5

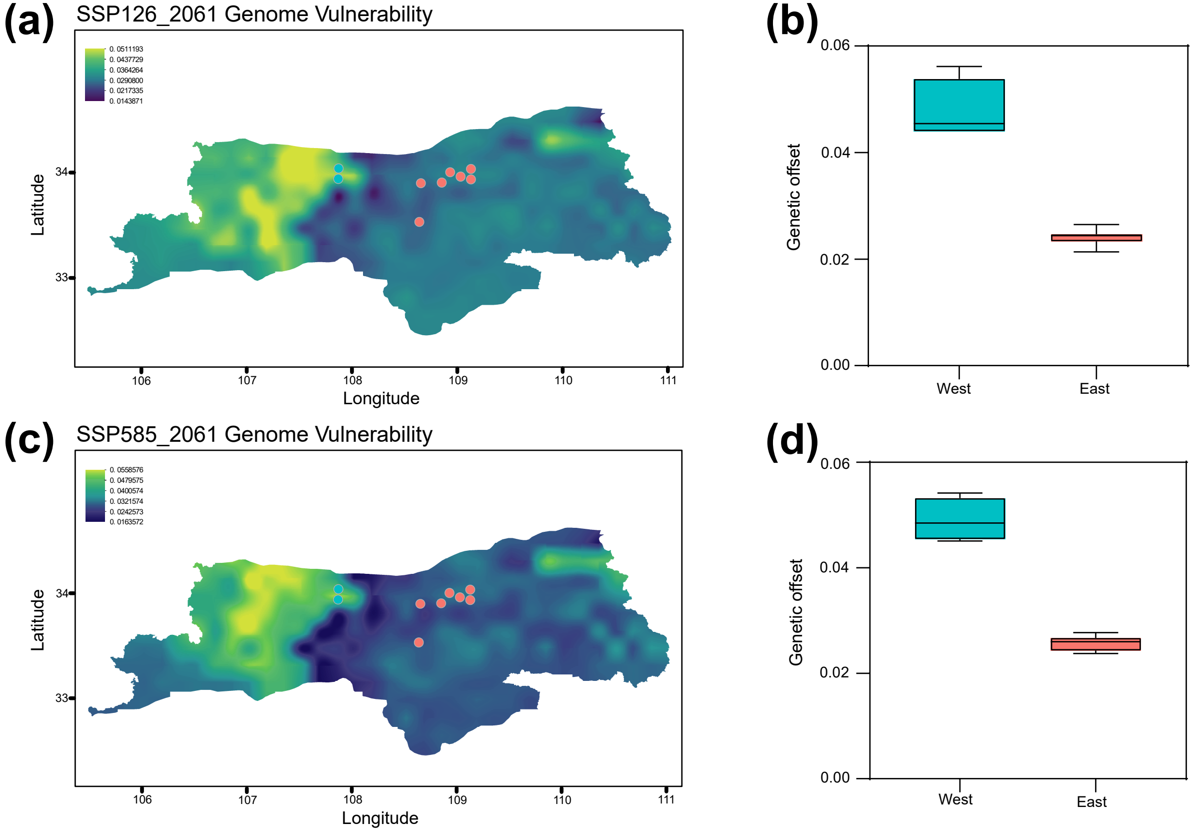

Supplementary figures

Click here to access/download
Supplementary Material
supplementary_material_figures_GigaScience-revised2.docx

Supplementary tables

Click here to access/download
Supplementary Material
supplementary_material_tables_GigaScience-revised.xlsx
