# Supplementary material for: The chromosome-level genome assembly of an endangered herb Bergenia scopulosa provides insights into local adaptation and genomic vulnerability under climate change
Source: Gigascience. 2024 Nov 28;13:giae091. doi: 10.1093/gigascience/giae091 (PMC11604060; doi:10.1093/gigascience/giae091)
Supplement: giae091_Response_to_Reviewer_Comments_Original_Submission [file giae091_response_to_reviewer_comments_original_submission.pdf]

Author's Response To Reviewer Comments

Reviewer #1
Major point
Q: 1. Figure 2a, why selected these ten species in the phylogenetic tree? Bergenia scopulosa (Saxifragaceae family) and Kalanchoe fedtschenkoi (Crassulaceae family) were closely related species in the constructed phylogenetic tree. B. scopulosa and K. fedtschenkoi diverged approximately 103.6 million years ago. B. scopulosa and Vitis vinifera diverged approximately 108.0 million years ago. Actually, V. vinifera and K. fedtschenkoi were distantly related to B. scopulosa. Thus, it is unreasonable that B. scopulosa expanded and contracted gene families were analyzed based on this constructed phylogenetic tree (line 176 and 183). Tiarella polyphylla and B. scopulosa belongs to the same Saxifragaceae family, and the T. polyphylla genome has been public. This species should be analyzed in the phylogenetic tree. Additionally, the genome of Hamamelis virginiana, Paeonia ostia, Paeonia ludlowii, Paeonia suffruticosa, Cercidiphyllum japonicum and Penthorum chinense, which belong to Saxifragales, have been sequenced. These species are better for analysis in the phylogenetic tree.
A: Thank you very much for your detailed and insightful comments on our manuscript. We appreciate the time and effort you have put into reviewing our work. We fully agree with your suggestions regarding the selection of species in our phylogenetic tree. As you pointed out, the inclusion of additional species, particularly those belonging to the same family or order as our focal species, would provide a more comprehensive and robust analysis of gene family evolution. In response to your feedback, we have revised our phylogenetic analysis by incorporating T. polyphylla, which belongs to the Saxifragaceae family and has a publicly available genome. Additionally, we are actively exploring the possibility of including other species from the Saxifragales order, such as, Paeonia ostii, P. ludlowii, P. suffruticosa pending the availability of suitable genome sequences. We believe that these changes will significantly strengthen our conclusions and improve the overall quality of our manuscript. We are grateful for your guidance and support in refining our work. Once again, thank you for your valuable contributions to our research. We look forward to your continued feedback as we move forward with this study.

Q: 2. Line 200 and Fig S5, it is unreasonable for a single population to represent the West lineage. Thus, these West lineage results based on a single population were unreliable (line 290). Additional populations are needed to establish the West lineage.
A: Thank you for bringing up the important concern regarding the representativeness of the West lineage in our study. We fully acknowledge that relying solely on a single population to characterize the West lineage might limit the robustness and generalizability of our conclusions. In response to your valuable feedback, we have undertaken a renewed field investigation and, as a result, successfully identified and included an additional population from the vicinity of Taibai Mountains. This newly discovered population not only enriches our sampling of the West lineage but also presents a valuable opportunity to further expand and strengthen our analytical framework. By incorporating these fresh samples, we aim to gain a more comprehensive understanding of the species' distribution, genetic diversity, and potentially, its evolutionary history. Specifically, we have conducted resequencing of individuals from this new population and integrated their data with the previous 32 individuals in our study. The expanded dataset now includes 5 additional individuals from the new population, allowing us to perform a more comprehensive analysis of the West lineage. We have revised the relevant sections of the manuscript, including Line 200 and Figure S5, to reflect these updates. Our revised results show that the inclusion of the new population has not only increased the sample size but also improved the statistical support for the West lineage, making our conclusions more reliable and robust. We are grateful for your constructive criticism, which has led to the improvement of our study. We believe that the inclusion of this additional population strengthens our analysis and enhances the overall quality of our work.

Minor point
Q: 1. Line 150, provide detailed species information used for homology-based gene prediction.
A: Thank you for incorporating the suggested detail into your manuscript. Within the Genome annotation in Materials and Methods section, we have enhanced our methodology by providing detailed information on the homology-based gene prediction approach. Specifically, we utilized Arabidopsis thaliana, Beta vulgaris, Salvia bowleyana, K. fedtschenkoi, Oryza sativa, Rhodiola crenulate and Solanum lycopersicum as

reference species, chosen for their phylogenetic proximity and well-annotated genomes.

Q: 2. Line 272, provide the full name of the FAD.
A: Thank you for pointing out the need to clarify the full name of the FAD. In Line 301, the abbreviation FAD refers to Omega-3 fatty acid desaturase 7. We have revised the manuscript to include this full name for clarity.

Q: 3. Line 459, provide detailed plant material information used for total genomic DNA extraction, such as whether the sequenced plant material is from a population or a single plant, and the source of the RNA-Seq samples.
A: Thank you for your inquiry about the plant material used for total genomic DNA extraction and the source of the RNA-Seq samples. We have clarified in the revised manuscript that the genomic DNA was extracted from a single plant in the Qinling Mountains, Shaanxi Province, China (N33°57'42", E109°3'37") and the RNA-Seq samples were derived from the same population. This ensures consistency and reliability of our results. The updated information is now included in the Materials and Methods section.

We have prominently highlighted the modified manuscript to ensure clarity. Once again, we extend our sincere gratitude for your invaluable and constructive feedback.

Reviewer #2
Major suggestions:
Q: 1. The authors said there are two distinct lineages. However, it seems three group (red, green, and mixed color) from K=2 of Fig. 3a. More evidence was needed to support the conclusion.
A: Thank you for your thoughtful review and valuable feedback on our manuscript. We appreciate your attention to the detail regarding the genetic lineage distinction within our species, B. scopulosa. In response to your comment, we have re-examined the population genomic analysis with a focus on the newly sampled five individuals, which has led us to several important insights.
Firstly, regarding the observation that Figure 3a at K=2 seems to suggest three groups (red, green, and mixed color), we acknowledge the initial ambiguity and have conducted a more rigorous analysis to clarify this point. Our detailed analysis of the cross-validation error (cv error) across different K values indeed shows that the difference in cv error between K=2 and K=3 is minimal, indicating that both models provide statistically comparable fits to the data. However, the interpretation of these models in the context of geographical and ecological factors becomes crucial.
Specifically, upon examining the geographic distribution of the genetic clusters at K=2, we find a clear demarcation between two distinct lineages that correspond to the western and eastern regions of the Qinling Mountains. This geographical correspondence is highly consistent with known ecological and environmental factors influencing the species, reinforcing the biological relevance of the K=2 model. Furthermore, while K=3 (best K) does reveal an additional level of genetic differentiation within the eastern lineage, this further subdivision does not alter the fundamental distinction between the eastern and western lineages. The western lineage remains relatively stable, suggesting a more uniform genetic background, whereas the eastern lineage exhibits internal heterogeneity, likely due to local adaptations or historical demographic events.
Given these considerations, we have chosen to focus our subsequent analysis on the two geographically distinct lineages (K=2) as they represent the primary and biologically meaningful division within B. scopulosa. This approach allows us to investigate key evolutionary questions related to the species' adaptation, divergence, and potential conservation implications.
We have updated the manuscript to include a more detailed discussion of these findings, including the analysis of cv error, the geographical correlation of the genetic clusters, and the biological implications of our choice to focus on the two primary lineages. We hope this clarifies our rationale and strengthens the conclusions presented in our work.
Thank you again for your constructive comments, which have significantly improved the quality and clarity of our manuscript.

Q: 2. There are one population of west lineages from Figure 5a,c. Does it affect the estimation of offset and vulnerability?
A: Thank you for your continued attention to our manuscript and for bringing up the important observation regarding the newly discovered population in the western lineages from Figure 5a and 5c. We appreciate

your careful review and the opportunity to clarify this point.
Regarding your question about whether the inclusion of this additional population from the western lineages affects the estimation of offset and vulnerability, we have carefully re-examined our data and analyses. The inclusion of this new population does not alter the overall conclusion that the western lineages exhibit higher genomic vulnerability.
Our re-sequencing analysis of this additional population was integrated seamlessly with the existing 32 individual datasets, allowing for a more comprehensive understanding of the genetic diversity and vulnerability within the species. The analysis confirmed that the western lineages maintain a higher level of genomic vulnerability, likely due to a combination of factors including population size, genetic drift, and potential environmental pressures.
We acknowledge that the species, B. scopulosa, is listed as a vulnerable species under the first batch of local key protected plants in Shaanxi Province, China. This classification reflects its limited distribution and potential threats to its survival in the wild. Our analysis, including the newly discovered population, provides further evidence of the need for conservation efforts to protect this species and its unique genetic diversity.
We are encouraged by your interest in our work and hope that the expanded dataset and re-analysis have addressed your concerns. We believe that our findings, including the updated analysis incorporating the new population, provide valuable insights into the evolutionary history and conservation status of B. scopulosa. We look forward to your continued support and approval of our manuscript.

Q: 3. Many t2t and/haplotype-resolved genomes have been published: https://academic.oup.com/hr/pages/t2t-reference-genomes-for-horticultural-crops-si the authors should compare your assemblies with the published ones.
A: Thank you for your insightful comment. We have indeed discussed the limitations of our genome assemblies in comparison to T2T and haplotype-resolved genomes, citing relevant literature to support our discussion. Your suggestion highlights the importance of this comparison and provides valuable insights for our future research directions. We appreciate your guidance and will continue to explore ways to improve our assemblies.

Minor suggestions:
Q: Figures: the text on the figure is too small. It is hard to read.
A: Thank you for bringing this to my attention. I apologize for the inconvenience caused by the small text size in the figures. I understand that it may have made it difficult for you to read the details clearly.
To address this issue, I will revise the figures by increasing the font size of the text, ensuring that it is legible and easy to read. I will also carefully check the overall layout of the figures to ensure that the revised text does not overlap or compromise the clarity of other elements within the figures.
I appreciate your feedback, and I am confident that the revised figures will enhance the readability and overall quality of the manuscript. Thank you again for your time and valuable suggestions.

Q: Line 221, the results of LFMM and RDA should show some gene examples, not only GO term.
A: Thank you for your insightful comment regarding the presentation of results at line 221. In response, I have revised the manuscript to include specific gene examples, in addition to GO terms, from the LFMM and RDA analyses. This revision has been completed to provide a more comprehensive and detailed understanding of the outcomes and allow readers to gain a deeper insight into the biological processes and pathways that are being modulated.
I carefully selected the gene examples based on their statistical significance and relevance to the overall findings of the study, ensuring that the revised text is clear and concise while highlighting the key takeaways from the analysis.
Thank you again for your valuable suggestion. I believe that the inclusion of gene examples has significantly enhanced the quality and impact of the manuscript.

Q: Line243-244, how many overlapped SNP markers between LFMM and RDA?
A: Thank you for your query regarding the number of overlapped SNP markers between LFMM and RDA. In our analysis, we identified a total of 931 overlapped SNP markers that were significant in both LFMM and RDA.
This overlap suggests that these particular SNP markers are consistently associated with the factors being investigated across both analytical approaches. We believe that these markers may play important roles in

the underlying biological processes and pathways being modulated by these factors.
We have included this information in the revised manuscript, specifically at line 281 to provide clarity and transparency to the reader. Thank you for bringing this to our attention, and we hope that this clarification enhances the overall quality and understandability of the manuscript.

Q: Line 272, how about the allele frequency of SNP markers of FAD7 gene in different Bergenia scopulosa populations?
A: Thank you for your inquiry regarding the allele frequency of SNP markers within the FAD7 gene across different B. scopulosa populations. In our analysis, we have examined the allelic frequency distributions of several key genes, including FAD7, to gain insights into the genetic variation and potential adaptive mechanisms within the species.
Specifically, concerning the FAD7 gene, our results indicate an interesting pattern in the allelic frequency distribution between the western and eastern populations of B. scopulosa. While some genes in the western lineage exhibited rare alleles, the FAD7 gene stands out with a notable pattern: it shows a predominance of homozygous alleles in the western populations and a higher frequency of heterozygous alleles in the eastern populations.
This observation is intriguing and suggests that the allelic homozygosity versus heterozygosity of core genes, such as FAD7, might contribute to the differential vulnerability or adaptability of these populations. The homozygosity of FAD7 alleles in the western populations could potentially indicate a more uniform or stable genetic background, while the higher heterozygosity in the eastern populations may suggest increased genetic diversity and potential for adaptation.
We hypothesize that this variation in allelic frequency distributions, particularly for genes involved in important physiological processes like fatty acid desaturation (as in the case of FAD7), could be linked to differential environmental pressures or selection pressures acting on these populations. For example, the western populations may have undergone stronger purifying selection, leading to the fixation of advantageous alleles, while the eastern populations may have experienced more relaxed selection or introgression of alleles from other populations, promoting genetic diversity and heterozygosity.

Q: Reference: please consider cite for climate adaptation related papers, such as (Shi et al, 2024, Molecular plant) and (Zhang et al, 2024, Molecular plant).
A: Thank you for your thoughtful review and the valuable suggestions regarding the inclusion of additional references. In response to your recommendation, I have carefully considered the papers you mentioned: (Shi et al, 2024, Molecular Plant) and (Zhang et al, 2024, Molecular Plant), both of which focus on climate adaptation in plants.
I have now incorporated these references into the manuscript to strengthen the discussion on climate adaptation aspects relevant to our study. Specifically, I have cited (Shi et al, 2024) in the context of understanding the molecular mechanisms underlying plant responses to changing climatic conditions, particularly highlighting their pioneering study on the Populus super-pangenome, which reveals the pivotal role of private genes and their evolutionary links to regulatory and epigenetic landscapes, facilitating environmental adaptation, while (Zhang et al, 2024) has been referenced to highlight the evolutionary dynamics of climatic adaptation and uncovering climate-adapted variation can empower the breeding of climate-resilient crops to face future climate change.

We have clearly emphasized the revised manuscript through highlighting, enhancing readability. We reiterate our heartfelt appreciation for your extremely helpful and insightful comments.
