# Supplementary material for: The chromosome-level genome assembly of an endangered herb Bergenia scopulosa provides insights into local adaptation and genomic vulnerability under climate change
Source: Gigascience. 2024 Nov 28;13:giae091. doi: 10.1093/gigascience/giae091 (PMC11604060; doi:10.1093/gigascience/giae091)
Supplement: giae091_Response_to_Reviewer_Comments_Revision_1 [file giae091_response_to_reviewer_comments_revision_1.pdf]

Author's Response To Reviewer Comments

Reviewer reports:
Reviewer #1: The author assembled a high-quality, chromosome-level genome of the rare and endangered herb Bergenia scopulosa and re-sequenced genomes of 37 individuals that covered the whole geographic distributions of species. By integrating population genetics, landscape genomics, and climate datasets, a substantial number of adaptive SNP loci associated with climate variables were identified. Population genomic analysis revealed two genetic lineages in B. scopulosa. The western lineage was found to exhibit higher genomic vulnerability and greater abundance of rare cold-tolerance alleles. However, additional modification may be needed to improve the manuscript as listed below before acceptance.

Q: 1. Page 10 line 178, the description "B. scopulosa has undergone two distinct WGD events" required more data support, such as the syntenic depths analysis between B. scopulosa and grape.
A: Thank you very much for your thorough review of our manuscript and for the valuable suggestion regarding the need for more data support for the statement "B. scopulosa has undergone two distinct WGD events." We have taken your advice seriously and have conducted additional analyses to strengthen our conclusion.
In response to your recommendation, we have included an analysis of the distribution of the four-fold synonymous third-codon transversion rate (4DTv) for paralogous genes within the genomes of B. scopulosa, Tiarella polyphylla, and Vitis vinifera, as well as orthologous genes between B. scopulosa and V. vinifera. These results are presented in Supplementary Fig. S5, which provides further evidence for the two distinct WGD events experienced by B. scopulosa.
Specifically, Supplementary Fig S5(a) shows the 4DTv distribution, highlighting the gamma WGD event that is shared by B. scopulosa, V. vinifera, and T. polyphylla, as indicated by the red arrow. These peaks in the 4DTv distribution serves as a clear marker of the WGD event and aligns with our hypothesis. Moreover, Supplementary Fig S5(b) presents the syntenic depths between the genomes of B. scopulosa and V. vinifera. The patterns of syntenic depth and distribution provide additional support for the occurrence of two distinct WGD events in B. scopulosa.
We believe that these additional analyses and results presented in Supplementary Fig. S5 significantly strengthen our conclusion regarding the WGD history of B. scopulosa. We are grateful for your insightful comments and suggestions, which have helped us to improve the quality and rigor of our work.
Thank you again for your time and effort in reviewing our manuscript. We look forward to any further feedback you may have as we continue to refine and improve our work.

Q: 2. Page 11 line 201, the description "the expansion and contraction of these genes enhance the adaptability of B. scopulosa in complex environments" is just one possibility in the absence of experimental validation. When referring to different species, these expanded and contracted genes will exhibit significant differences.
A: Thank you for your thoughtful comments and suggestions regarding our manuscript. You are absolutely correct in pointing out that the statement "the expansion and contraction of these genes enhance the adaptability of B. scopulosa in complex environments" is indeed a hypothesis that requires further experimental validation. In the absence of comprehensive experimental data for all expanded and contracted genes, such a conclusion can only be made tentatively.
To address your concerns, we have revised the relevant section of our manuscript to reflect a more cautious and inferential tone. Specifically, we have emphasized that the observed gene expansions and contractions are intriguing patterns that suggest potential roles in adaptability.
Moreover, we would like to highlight that we have taken initial steps towards validating the function of some of these genes. In particular, we have conducted transgenic experiments focusing on an expanded gene, BsUGT74E2 (Bsco_038285), which shows homology to the Arabidopsis UGT74E2. Our preliminary results suggest a role for this gene in cold tolerance, providing a promising starting point for future investigations.
We fully agree that the functional validation of these genes is crucial for a comprehensive understanding of their roles in B. scopulosa's adaptability. As such, we have included a discussion of our ongoing and future plans to conduct more extensive experimental validation of the expanded and contracted gene sets. We are committed to pursuing this research direction and hope to contribute to a deeper understanding of the

genetic basis of adaptability in this and other species.
Thank you again for your valuable feedback. We appreciate your time and effort in reviewing our work, and we look forward to incorporating your suggestions into our future research endeavors.
