# Supplementary material for: The chromosome-level genome assembly of an endangered herb Bergenia scopulosa provides insights into local adaptation and genomic vulnerability under climate change
Source: Gigascience. 2024 Nov 28;13:giae091. doi: 10.1093/gigascience/giae091 (PMC11604060; doi:10.1093/gigascience/giae091)
Supplement: giae091_Reviewer_2_Report_Original_Submission — Yongfeng Zhou -- 6/11/2024 [file giae091_reviewer_2_report_original_submission.pdf]

Reviewer Report

Title: The chromosome-level genome assembly of an endangered herb Bergenia scopulosa provides insights into local adaptation and genomic vulnerability under climate change

Version: Original Submission **Date:** 6/11/2024

Reviewer name: Yongfeng Zhou

Reviewer Comments to Author:

This study has successfully constructed high quality Bergenia scopulosa genome, with population genetic analysis of Bergenia scopulosa in climate adaptation. The authors had identified a group of environmental adaptive SNPs and candidate genes. The authors also conducted gene family and qPCR analysis to verify the candidate genes. Furthermore, the genomic vulnerability of all related population was estimated, and provide guidance for the conservation of Bergenia scopulosa. Overall, this study has great design, well organized and the analyses performed are thoughtful. I only have few inquiries that I believe should be addressed to enhance the manuscript:

Major suggestions:

1. The authors said there are two distinct lineages. However, it seems three group (red, green, and mixed color) from K=2 of Fig3a. More evidence was needed to support the conclusion.
2. There are one population of west lineages from Figure 5a,c. Does it affect the estimation of offset and vulnerability?
3. Many t2t and/haplotype-resolved genomes have been published: https://academic.oup.com/hr/pages/t2t-reference-genomes-for-horticultural-crops-si the authors should compare your assemblies with the published ones.

Minor suggestions:

Figures: the text on the figure is too small. It is hard to read.

Line 221, the results of LFMM and RDA should show some gene examples, not only GO term.

Line243-244, how many overlapped SNP markers between LFMM and RDA?

Line 272, how about the allele frequency of SNP markers of FAD7 gene in different Bergenia scopulosa populations?

Reference: please consider cite for climate adaptation related papers, such as (Shi et al, 2024, Molecular plant) and (Zhang et al, 2024, Molecular plant).

Methods

Are the methods appropriate to the aims of the study, are they well described, and are necessary controls included? Choose an item.

Conclusions

Are the conclusions adequately supported by the data shown? Choose an item.

Reporting Standards

Does the manuscript adhere to the journal's guidelines on minimum standards of reporting? Choose an item.

Choose an item.

Statistics

Are you able to assess all statistics in the manuscript, including the appropriateness of statistical tests used? Choose an item.

Quality of Written English

Please indicate the quality of language in the manuscript: Choose an item.

Declaration of Competing Interests

Please complete a declaration of competing interests, considering the following questions:

- Have you in the past five years received reimbursements, fees, funding, or salary from an organisation that may in any way gain or lose financially from the publication of this manuscript, either now or in the future?
- Do you hold any stocks or shares in an organisation that may in any way gain or lose financially from the publication of this manuscript, either now or in the future?
- Do you hold or are you currently applying for any patents relating to the content of the manuscript?
- Have you received reimbursements, fees, funding, or salary from an organization that holds or has applied for patents relating to the content of the manuscript?
- Do you have any other financial competing interests?
- Do you have any non-financial competing interests in relation to this paper?

If you can answer no to all of the above, write 'I declare that I have no competing interests' below. If your reply is yes to any, please give details below.

no

I agree to the open peer review policy of the journal. I understand that my name will be included on my report to the authors and, if the manuscript is accepted for publication, my named report including any attachments I upload will be posted on the website along with the authors' responses. I agree for my report to be made available under an Open Access Creative Commons CC-BY license (http://creativecommons.org/licenses/by/4.0/). I understand that any comments which I do not wish to be included in my named report can be included as confidential comments to the editors, which will not be published.

Choose an item.

To further support our reviewers, we have joined with Publons, where you can gain additional credit to further highlight your hard work (see: https://publons.com/journal/530/gigascience). On publication of this paper, your review will be automatically added to Publons, you can then choose whether or not to claim your Publons credit. I understand this statement.

Yes Choose an item.
